# FAM172A promotes epithelial ovarian cancer progression and induces platinum resistance via the PI3K/AKT pathway

**DOI:** 10.1038/s41598-025-26676-9

**Published:** 2025-12-03

**Authors:** Yuanyuan Wu, Jie Ma, Bo Wu, Zhixiang Li, Yan Li, Chenglei Zhang, Lijuan Wang, Jiarui Li, Yanru Ren, Yi Yang

**Affiliations:** 1 School of Basic Medical Sciences, Ningxia Medical University, 1160 Shengli Street, Yinchuan750000 China, Yinchuan, 750000 China; 2 Department of Oncology, Cancer Hospital, General Hospital of Ningxia Medical University, Yinchuan, 750000 China; 3 Department of oncological surgery, Cancer Hospital, General Hospital of Ningxia Medical University, Yinchuan, 750000 China; 4 Clinical Medical College of Ningxia Medical University, Yinchuan, 750000 China; 5 Medical Laboratory, General Hospital of Ningxia Medical University, Yinchuan, 750000 China; 6 Department of Endocrinology, General Hospital of Ningxia Medical University, Yinchuan, 750000 China

**Keywords:** FAM172A, Ovarian cancer, Prognosis, PI3K-Akt signaling pathway, Cisplatin sensitivity, Diseases, Oncology

## Abstract

This study aimed to investigate the role of FAM172A in epithelial ovarian cancer (EOC), a highly lethal gynecological malignancy often diagnosed at late stages with limited treatment options. FAM172A expression was evaluated in EOC and normal ovarian tissues using western blotting and immunohistochemistry, and its association with patient prognosis, treatment response, and CA125 levels was assessed by multivariate regression analysis. Functional assays were performed to examine the effects of FAM172A on EOC cell proliferation, migration, and invasion. In vivo models were used to evaluate the influence of FAM172A on tumor growth, metastasis, and chemosensitivity. The underlying mechanism was explored by modulating the PI3K-Akt pathway with pharmacological inhibitors and activators. FAM172A was significantly upregulated in EOC tissues, and its elevated expression correlated with poor prognosis, chemotherapy resistance, and increased CA125 levels. Multivariate analysis identified FAM172A expression, platinum sensitivity, and CA125 as independent prognostic factors. In vitro, FAM172A promoted malignant behavior and conferred resistance to cisplatin. In vivo, knockdown of FAM172A suppressed tumor progression and enhanced the efficacy of cisplatin. Mechanistically, FAM172A exerted its effects through regulation of the PI3K-Akt pathway, and modulation of PI3K signaling rescued FAM172A-induced phenotypic changes. These findings highlight FAM172A as a critical promoter of EOC progression, associated with aggressive tumor characteristics and treatment failure. By activating the PI3K-Akt pathway, FAM172A represents a promising therapeutic target for EOC, potentially offering new strategies to improve patient outcomes, particularly in overcoming chemoresistance.

**Supplementary Information:**

The online version contains supplementary material available at 10.1038/s41598-025-26676-9.

## Introduction

Ovarian cancer (OC) is a leading cause of cancer-related mortality among women worldwide^1^. According to World Health Organization estimates for 2020, there were approximately 55,342 new OC cases in China (ranking tenth among all female malignancies) and 37,519 deaths (ranking ninth among all female malignancies)^2^. Epithelial ovarian cancer (EOC), which arises from the ovarian surface epithelium, is the most prevalent histological subtype, accounting for about 90% of OC cases^3^. The lack of specific early symptoms often leads to diagnosis at advanced stages, contributing to a high recurrence rate^4^. EOC remains one of the gynecological malignancies with the poorest prognosis, exhibiting an overall five-year survival rate below 50%^5^. Although treatment strategies, including surgery and chemotherapy, have advanced, the overall survival for advanced EOC remains low. Platinum-based chemotherapeutic agents, such as cisplatin and carboplatin, have served as first-line treatments for EOC for decades. However, the emergence of platinum resistance and associated side effects presents a major therapeutic challenge^6,7^. Thus, identifying specific biomarkers for EOC is critical to enable early intervention and improve patient survival.

Family with sequence similarity 172 member A (FAM172A) is a functionally poorly characterized gene that is widely expressed in human tissues^8^. It has been implicated in the regulation of alternative splicing and in the pathogenesis of CHARGE syndrome^9^. Several studies suggest that FAM172A is involved in a range of human diseases, including cancer^10,11^, neurodegenerative disorders^12^, and psychiatric conditions^13,14^. Additionally, FAM172A appears to regulate cell proliferation, apoptosis, and cell cycle progression^15^. Previous work has linked FAM172A to poor prognosis in colorectal carcinoma^8^; however, its specific role in EOC tumorigenesis and chemotherapy resistance has not been clearly defined.

In this study, we sought to determine the functional role of FAM172A in EOC and its contribution to cisplatin resistance. Our results provide new insights into the clinical relevance of FAM172A in EOC progression and patient prognosis.

## Methods and materials

### Data collection

The GDC TCGA-OV dataset of 378 EOC tumor samples was downloaded from the UCSC XENA database, and 88 normal ovarian tissue samples were obtained from the GTEx dataset. Based on FAM172A expression levels, the tumor samples were divided into high and low expression groups, and survival analysis was performed using the corresponding clinical survival data. The correlation between drug sensitivity and the FAM172A gene was analyzed using the tumor expression profile data from the EOC dataset. The R package pRRophetic was used to predict the IC₅₀ values for cisplatin. Spearman’s correlation analysis was then applied to evaluate the relationship between FAM172A expression levels and the predicted drug IC₅₀ values.

The clinical specimens consisted of EOC tissues and normal ovarian tissues obtained from the General Hospital of Ningxia Medical University. The inclusion criteria for EOC tissues were as follows: (1) primary EOC cases diagnosed and treated at the hospital between 2012 and 2021, with no neoadjuvant chemotherapy prior to surgery; (2) pathological confirmation of EOC by the Department of Pathology; and (3) no history of malignant tumors in other organs or systems. The normal ovarian tissues were collected from patients who underwent oophorectomy for benign conditions and were confirmed as normal by senior pathologists. Based on these criteria, eight fresh EOC tissue samples and their adjacent non-cancerous ovarian tissues were collected and stored at −80 °C. Additionally, 150 paraffin-embedded EOC tissue blocks and 67 paraffin-embedded normal ovarian tissue blocks were collected. This study was approved by the Ethics Committee of the General Hospital of Ningxia Medical University (Approval No: 2021112), and all patients provided written informed consent for specimen use before surgery.

### Clinical specimen baseline data

The study included 150 patients with EOC, aged from 33 to 79 years (median age 56 years). Of these, 42 patients were aged 50 or younger, and 108 were over 50 years old. Pathological types consisted of 105 cases of serous adenocarcinoma, 28 cases of clear cell carcinoma, 6 cases of mucinous adenocarcinoma, 8 cases of endometrioid carcinoma, and 3 unclassified cases. Pathological grading included 3 cases of grade 3, 10 cases of grade 1, and 5 cases of grade 2. According to the International Federation of Gynecology and Obstetrics (FIGO) staging system, the majority of cases were stage III (99 cases), followed by stage IV (23 cases), stage II (21 cases), and stage I (7 cases) (Table 1). Patient data were obtained by reviewing outpatient and inpatient medical records, supplemented by follow-up conducted via telephone, WeChat, and other communication methods. The follow-up endpoint for this study was June 1, 2023.

### Cell lines and animal models

The human EOC cell line OVCAR-3 was obtained from Wuhan Promega Life Technology Co., Ltd., and the human ovarian surface epithelial cell line IOSE-80 was acquired from Cyagen (Shanghai) Biotechnology Co., Ltd. The human EOC cell lines A2780 and SKOV3 were preserved in the laboratory of the School of Basic Medical Sciences, Ningxia Medical University. A2780 cells were cultured in DMEM medium supplemented with 10% fetal bovine serum (FBS) and 1% penicillin/streptomycin (P/S). OVCAR-3 cells were maintained in RPMI-1640 medium containing 20% FBS, 10 µg/mL insulin, and 1% P/S. SKOV3 cells and IOSE-80 cells were cultured in RPMI-1640 medium with 10% FBS and 1% P/S. Cells in the logarithmic growth phase were routinely digested, centrifuged, resuspended, and seeded into six-well plates. After 24 h, a 1 mg/mL cisplatin solution was prepared and diluted with complete medium to the appropriate concentration, added to the plates, and incubated for 48 h in a cell culture incubator prior to cell collection. Female BALB/c nude mice aged 4–6 weeks were provided by the Animal Center of Ningxia Medical University.

### Immunohistochemistry (IHC) staining

Tissue blocks were mounted on a microtome, and consecutive 4-µm sections were prepared. Sections were placed in a 60 °C oven for 1 h, deparaffinized in xylene, and hydrated through a graded ethanol series (100%, 95%, 80%, and 70%). Antigen retrieval was performed using a high-pressure cooker with EDTA antigen retrieval buffer (pH 8.0; ZSGB-BIO, ZLI-9067). Endogenous peroxidase activity was quenched by incubating sections in 3% H₂O₂ for 10 min. Nonspecific binding was blocked with 10% bovine serum albumin (BSA) for 40 min. Sections were then incubated with 50–80 µL of appropriately diluted primary antibody against FAM172A (ab121364, Abcam; 1:200 dilution) in a humidified chamber at 37 °C for 1 h. After washing with PBS, sections were incubated with 50–80 µL of diluted HRP-conjugated secondary antibody (Goat anti-Rabbit IgG-HRP, ab205718, Abcam; 1:5000) at 37 °C for 30 min. Following additional PBS washes, color development was carried out using freshly prepared DAB substrate (ZSGB-BIO, ZLI-9017/9018/9019) at room temperature. Staining was monitored under a microscope, with positive signal defined as clear and specific staining with minimal background. Sections were counterstained with hematoxylin for 40 s, rinsed in tap water for 1 min, dehydrated through a graded ethanol series (50%, 70%, 90%, 95%, and 100%), cleared in xylene, and mounted with neutral gum.

### Western blot analysis

Approximately 100 mg of solid tissue was placed in a clean, pre-cooled culture dish and homogenized on ice with 300–500 µL of lysis buffer. The homogenate was centrifuged at 4 °C for 5 min, and the supernatant protein concentration was determined using a BCA assay kit (Jiangsu KeyGEN BioTECH Corp., Ltd.). Protein samples (30 µg per lane) were separated by SDS–PAGE and transferred to a pre-cut PVDF membrane. The membrane was blocked with 10% skim milk prepared in TBST for 2 h at room temperature, followed by incubation with primary antibody overnight at 4 °C. The following primary antibodies were used: FAM172A (ab121364, Abcam) and GAPDH (AF7021, Affinity Biosciences). The next day, the membrane was warmed to room temperature and washed three times with TBST under gentle agitation. It was then incubated with HRP-conjugated goat anti-rabbit IgG secondary antibody (ZSGB-BIO, ZB-2301) for 1 h at room temperature. After washing, the membrane was covered with a chemiluminescent substrate and imaged using a digital imaging system. Original western blot images are provided in the Supplementary Information.

### Plasmid extraction and cell transfection

Bacterial strains harboring the target plasmid were inoculated into LB medium (10 g/L NaCl, 10 g/L tryptone, 5 g/L yeast extract) containing appropriate antibiotics and cultured in a shaking incubator at 37 °C for 16 h. The bacterial culture was centrifuged at 10,000 × g for 1 min to pellet the cells. After resuspension and lysis, the sample was centrifuged at 10,000 × g for 10 min, and the supernatant was applied to a HiBind DNA binding column. HB buffer and DNA wash buffer were sequentially added to the column, followed by centrifugation. Plasmid DNA was eluted with 50 µL of elution buffer. The concentration and purity of the extracted plasmid DNA were assessed by UV spectrophotometry or agarose gel electrophoresis. Purified plasmid DNA was stored at − 20 °C. For transfection, nucleic acid and transfection reagent (Zeta Life Advanced Series High-Efficiency DNA/RNA Transfection Reagent, Zeta Life Inc., USA) were mixed and incubated at room temperature for 10–15 min. The resulting transfection complexes were added dropwise to the cell culture dish, which was then returned to the incubator for 24 h. The medium was subsequently replaced with complete culture medium. Transfection efficiency was evaluated 48 h post-transfection.

### Construction of stable cell lines with FAM172A overexpression and knockdown

Stable cell lines with either overexpression or knockdown of FAM172A were generated using lentiviral vector systems. The human FAM172A (h-FAM172A) overexpression lentiviral vector was constructed and packaged by Hanheng Biotechnology (Shanghai) Co., Ltd., and the FAM172A shRNA lentivirus was obtained from Jikai Gene Company. Prior to infection, polybrene was added to the culture medium at a final concentration of 8 µg/mL. Approximately 2 × 10⁵ cells in logarithmic growth phase were seeded into 3.5 cm culture dishes and cultured for 24 h until 70–80% confluency was reached. The diluted lentiviral supernatant was then added to the cells, which were incubated at 37 °C with 5% CO₂ for 24 h. After infection, the medium was replaced with fresh complete medium. GFP expression was assessed using fluorescence microscopy to estimate infection efficiency. Selection was performed using complete medium containing 2 µg/mL puromycin, with the medium changed every two days until stable polyclonal populations were established.

### CCK-8 assay

Cells in the logarithmic growth phase were digested, centrifuged, and counted. A 100 µL cell suspension containing 5,000–7,000 cells was seeded into each well of a 96-well plate and incubated for 24 h. The following day, cell density and morphology were examined under a microscope. Each experimental condition was performed in six replicate wells. According to the experimental design, gradient concentrations of cisplatin (100 µL per well) were added, and the plates were incubated for an additional 24 h. Then, 10 µL of CCK-8 reagent was added to each well, and incubation continued for 1 h. Absorbance was measured at 450 nm using a microplate reader. Cell viability was calculated as follows: Viability (%) = (Treatment group OD - Blank control OD)/(Negative control OD - Blank control OD) × 100%. The IC_50_ value, defined as the drug concentration that reduced cell viability by 50%, was determined by plotting viability against cisplatin concentration.

### Clone formation assay

Cells were cultured to the logarithmic growth phase, diluted to an appropriate density, and seeded evenly into culture dishes at a density of 500 cells per dish. The dishes were incubated in a constant-temperature incubator, and the complete culture medium was refreshed every three days. After visible colonies had formed, each dish was gently washed with phosphate-buffered saline (PBS). Cells were fixed by adding 3 mL of 4% paraformaldehyde solution and incubating at room temperature for 30 min, followed by another PBS wash. Then, 2 mL of 1% crystal violet staining solution was added to each dish, and staining was carried out at room temperature for 5–10 min. After thorough washing with PBS, the dishes were air-dried. The entire dish was photographed with a digital camera to obtain clear images of colony morphology. Colony formation was quantified using ImageJ software (version 1.4.3.67, National Institutes of Health, USA; https://imagej.nih.gov/ij/) with the ColonyArea plugin.

### Wound healing assay

Cells were seeded into six-well plates and cultured in a 5% CO₂ incubator at 37 °C until they reached 90–100% confluence, typically within 24–48 h. A single straight scratch was then introduced into the cell monolayer using a sterile 10 µL pipette tip. The well was gently rinsed with pre-warmed PBS to remove dislodged cells and debris. Images of the scratch were captured at the same location at regular intervals to monitor cell migration and wound closure. The scratch width or area was measured using ImageJ software (version 1.4.3.67, National Institutes of Health, USA; https://imagej.nih.gov/ij/). The percentage of scratch closure and the migration distance were calculated based on the initial and final scratch dimensions.

### Transwell assay

Transwell chambers were placed in a 24-well plate, and 500 µL of complete culture medium containing 20% serum was added to the lower chamber. For the migration assay, 200 µL of cell suspension containing 1 × 10⁴ cells was added to the upper chamber. For the invasion assay, 50–100 µL of diluted extracellular matrix gel was applied to the porous membrane of the Transwell insert and allowed to solidify. Then, 200 µL of cell suspension containing 2 × 10⁵ cells was carefully added on top of the gel. The plate was incubated at 37 °C with 5% CO₂ for 24 h. After incubation, the medium was removed, and non-migrating or non-invading cells on the upper surface of the membrane were gently wiped away with a cotton swab. The membrane was rinsed with PBS, fixed with 4% paraformaldehyde at room temperature for 30 min, and stained with 1% crystal violet for 10 min. Following a PBS wash, the cells on the lower surface of the membrane were imaged under a microscope. Cell counting was performed using image analysis software.

### Apoptosis detection by flow cytometry

Cells were digested using trypsin without EDTA, centrifuged at 1000 × g for 5 min, and resuspended in 100 µL of 1× Annexin V binding buffer. Then, 5 µL of Annexin V reagent was added, followed by 5 µL of propidium iodide (PI) staining solution, and the cells were incubated on ice in the dark for 30–40 min. Negative control cells were left unstained with Annexin V or PI, while single-stained controls (Annexin V only or PI only) were used for fluorescence compensation. After incubation, 400 µL of 1× Annexin V binding buffer was added, and samples were immediately analyzed by flow cytometry using the YF^®^647 A-Annexin V detection kit. Fluorescent signals were collected at 647 nm (APC channel) and approximately 617 nm (PI channel). In the bivariate dot plot, viable cells appeared in the lower left quadrant (Annexin V-negative/PI-negative), early apoptotic cells in the lower right quadrant (Annexin V-positive/PI-negative), late apoptotic and necrotic cells in the upper right quadrant (Annexin V-positive/PI-positive), and necrotic cells in the upper left quadrant (Annexin V-negative/PI-positive).

### Cell cycle analysis by flow cytometry

Cells were trypsinized, counted, and adjusted to a density of 1 × 10⁶ to 1 × 10⁷ cells/mL. The single-cell suspension was centrifuged at 1000 × g for 5 min, and the pellet was resuspended in 500 µL of ice-cold 70% ethanol for fixation overnight at 4 °C. After fixation, the cells were centrifuged at 1000 × g for 3 min, the ethanol was discarded, and the cell pellet was washed with PBS. Then, 500 µL of propidium iodide (PI)/RNase A staining solution (prepared in a 1:9 ratio) was added, and the cells were incubated in the dark at room temperature for 30–60 min. Prior to analysis, the cell suspension was passed through a 200-mesh nylon sieve. Flow cytometry was performed using a 488 nm excitation wavelength, and red fluorescence emission was recorded.

### Animal experiments

All animal procedures were conducted in accordance with relevant guidelines, and all methods received approval from the Animal Ethics Committee of the Ningxia Medical University Experimental Animal Center (Approval No. IACUC-NYLAC-2023-124). The methods are reported following the ARRIVE guidelines.

### Subcutaneous tumor model in nude mice

Six-week-old female nude mice were anesthetized via inhalation of isoflurane (3–5% for induction, 1–2% for maintenance). A 100 µL suspension containing 1 × 10⁶ SKOV3 cells with stable FAM172A knockdown or control cells was slowly injected subcutaneously into the left flank of each mouse. Tumor size and body weight were measured periodically using a caliper. Tumor volume was calculated as length × width² × 0.5. When the longest tumor diameter reached approximately 5 mm, mice were randomly assigned to four groups: control, FAM172A knockdown alone, cisplatin alone, and FAM172A knockdown plus cisplatin. Cisplatin was administered daily via intraperitoneal injection at 2 mg/kg. Mice were monitored daily, with body weight and tumor size recorded regularly. After 4 weeks of treatment, mice were euthanized by cervical dislocation. Tumor tissues were excised, photographed, measured, and weighed to evaluate treatment effects.

### Establishment of EOC peritoneal metastasis model in nude mice

A 1 mL cell suspension containing 5 × 10⁶ cells was injected into the lower left abdomen of 6-week-old female nude mice using a sterile syringe. The mice were then returned to the animal facility for routine maintenance. Body weight was monitored and recorded regularly. After 28 days of tumor growth, the mice were euthanized by cervical dislocation. The abdominal cavity was opened, and tumor tissues were carefully excised. Tumors were photographed and weighed. A portion of each tumor was fixed in 4% paraformaldehyde for histological analysis, and the remainder was stored at − 80 °C for further study.

## Results

### Elevated FAM172A expression is associated with poor prognosis in EOC patients

**Fig. 1:**
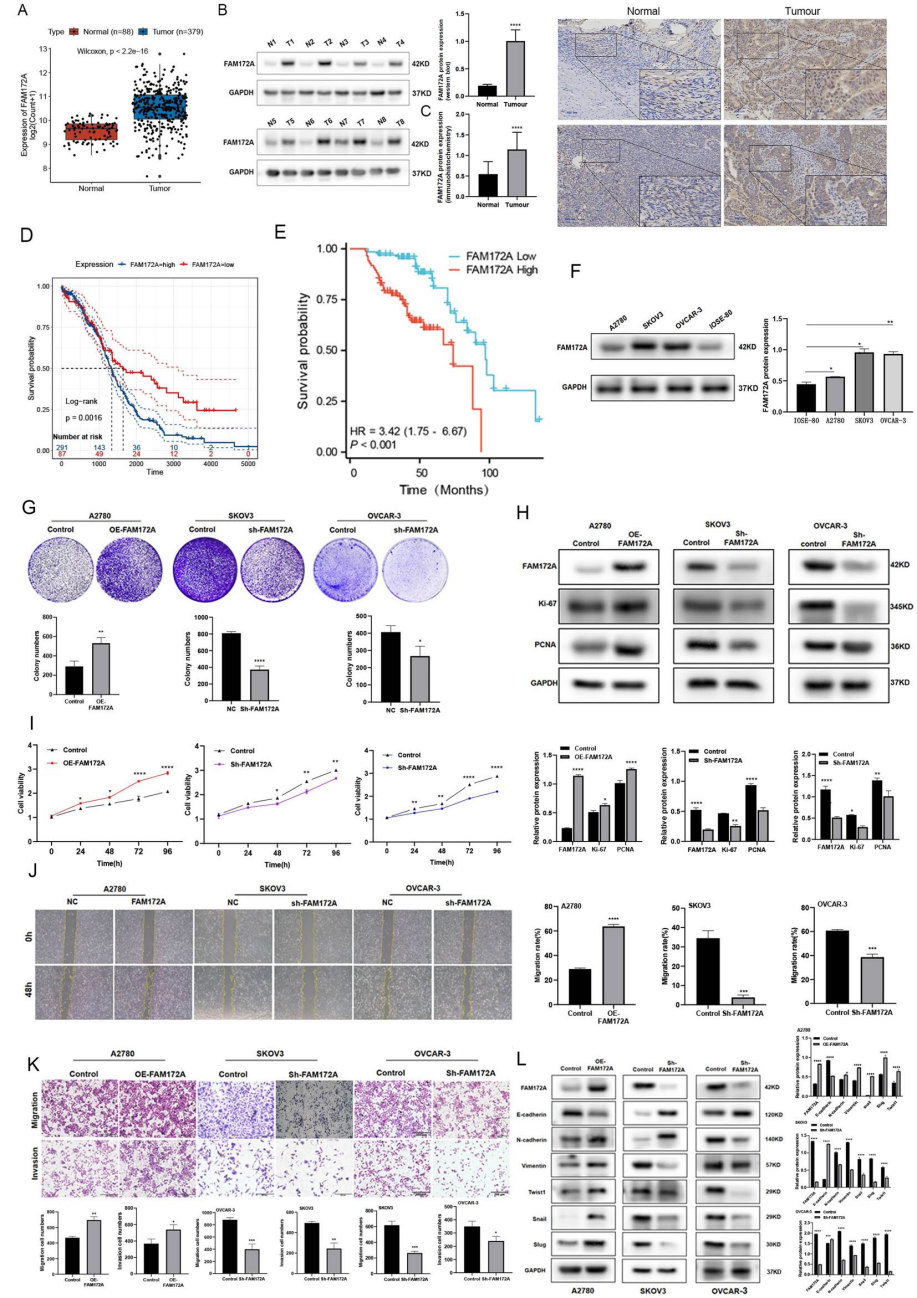
FAM172A is upregulated in EOC and promotes cell migration, invasion, and proliferation. (**A**) Analysis of FAM172A expression in EOC versus normal ovarian tissues using the UCSC Xena database. (**B**) FAM172A protein levels in EOC tissues compared to adjacent normal tissues. (**C**) Immunohistochemical staining of FAM172A in EOC and matched normal ovarian tissues. (**D**) Kaplan–Meier survival curves for high (blue) and low (red) FAM172A expression groups; dashed lines indicate 95% confidence intervals. (**E**) Poor prognosis associated with high FAM172A expression. (F) FAM172A protein expression in EOC cell lines and a normal ovarian epithelial cell line. (**G**) Colony formation assay in A2780 and SKOV3 cells after FAM172A knockdown or overexpression; the bar graph shows the number of qualified colonies. (H) Western blot analysis of proliferation-related proteins (KI67, PCNA) following FAM172A modulation. (**I**) CCK-8 assay assessing the effect of FAM172A on EOC cell proliferation. (**J**) Wound healing assay evaluating migration ability after FAM172A overexpression or silencing. (**K**) Transwell assay examining migration and invasion capacities. (**L**) Western blot analysis of epithelial–mesenchymal transition (EMT)-related proteins after FAM172A overexpression or knockdown. *P < 0.05, **P < 0.01, ***P < 0.001, ****P < 0.0001.

To investigate the role of FAM172A in epithelial ovarian cancer (EOC), we first analyzed its expression in EOC tissues and cell lines. Analysis of the TCGA database showed that FAM172A expression was significantly higher in EOC tissues than in normal ovarian tissues (Fig. 1A). Consistent with this finding, western blot analysis confirmed the upregulation of FAM172A protein in EOC samples relative to normal tissues (Fig. 1B). Immunohistochemistry (IHC) staining further validated these results (Fig. 1C). Additionally, FAM172A expression was higher in serous carcinoma than in non-serous carcinoma subtypes (Table S1). Elevated FAM172A expression was significantly associated with reduced sensitivity to platinum‐based chemotherapy and higher CA125 levels in patients (Tables S1–S3). Most importantly, high FAM172A expression was significantly correlated with poor prognosis in EOC patients (Fig. 1D–E), suggesting its potential utility as a prognostic biomarker. Using Cox regression analysis, we developed a nomogram to predict EOC prognosis. The model incorporated three independent risk factors: platinum exposure, FAM172A expression level, and CA125 level (U/mL) (Fig. S1A). The calibration curve demonstrated that the nomogram had satisfactory predictive accuracy for 1‐, 3‐, and 5‐year survival probabilities (Fig. S1B). We also assessed FAM172A expression in EOC cell lines (A2780, SKOV3, and OVCAR‐3) and the human ovarian surface epithelial cell line IOSE‐80. Western blot analysis revealed significantly higher FAM172A protein levels in EOC cells than in normal ovarian epithelial cells (Fig. 1F). Together, these findings support the significance of FAM172A in EOC progression and suggest its potential value as a prognostic indicator.

### FAM172A promotes cell Proliferation, Migration, and invasion in EOC

To further investigate the functional role of FAM172A in EOC, we overexpressed FAM172A in A2780 cells and knocked it down in SKOV3 and OVCAR-3 cells. Western blot analysis confirmed successful lentiviral infection and the corresponding changes in FAM172A protein levels (Fig. S2). FAM172A overexpression increased colony formation ability, while its knockdown reduced colony formation (Fig. 1G). Western blot analysis of proliferation markers showed that FAM172A overexpression upregulates KI67 and PCNA expression, whereas FAM172A knockdown downregulates their expression (Fig. 1H). Consistent with these findings, CCK-8 assays demonstrated that FAM172A overexpression enhanced cell proliferation in A2780 cells, while FAM172A suppression impaired proliferation in SKOV3 and OVCAR-3 cells (Fig. 1I). Wound healing and Transwell assays were performed to evaluate the role of FAM172A in migration and invasion. FAM172A overexpression significantly promoted wound closure in A2780 cells, while its silencing inhibited wound healing in SKOV3 and OVCAR-3 cells (Fig. 1J). Similarly, FAM172A upregulation enhanced migration and invasion capabilities, whereas its downregulation suppressed these abilities compared to controls (Fig. 1K). Western blot analysis further revealed that FAM172A overexpression increased the expression of N-cadherin, vimentin, Twist1, Snail, and Slug, and decreased E-cadherin expression. FAM172A knockdown produced the opposite effect on these epithelial–mesenchymal transition (EMT)-related markers (Fig. 1L). Taken together, these results indicate that FAM172A facilitates malignant behavior in EOC by promoting cell proliferation, migration, and invasion.

### FAM172A modulates cell cycle distribution in EOC cells

We next investigated the effect of FAM172A on cell cycle progression. Flow cytometry analysis revealed that FAM172A overexpression decreased the proportion of cells in G1 phase, whereas FAM172A knockdown increased the G1 population in SKOV3 and OVCAR-3 cells (Fig. S3A). To explore the mechanism of FAM172A-mediated cell cycle regulation, we underlying the expression of key cell cycle proteins. In A2780 cells, FAM172A overexpression elevated the levels of CDK6, Cyclin D1, Cyclin E1, and CDK4, but reduced the expression of P21, P27, and P53 expression (Fig. S3B). Conversely, FAM172A knockdown in SKOV3 and OVCAR-3 cells produced opposite effects on these proteins. These results suggest that FAM172A overexpression promotes the G1 to S phase transition, while its silencing induces G1 phase arrest, indicating that FAM172A plays a critical role in regulating cell cycle distribution in EOC cells.

### FAM172A inhibits apoptosis in EOC cells

**Fig. 2:**
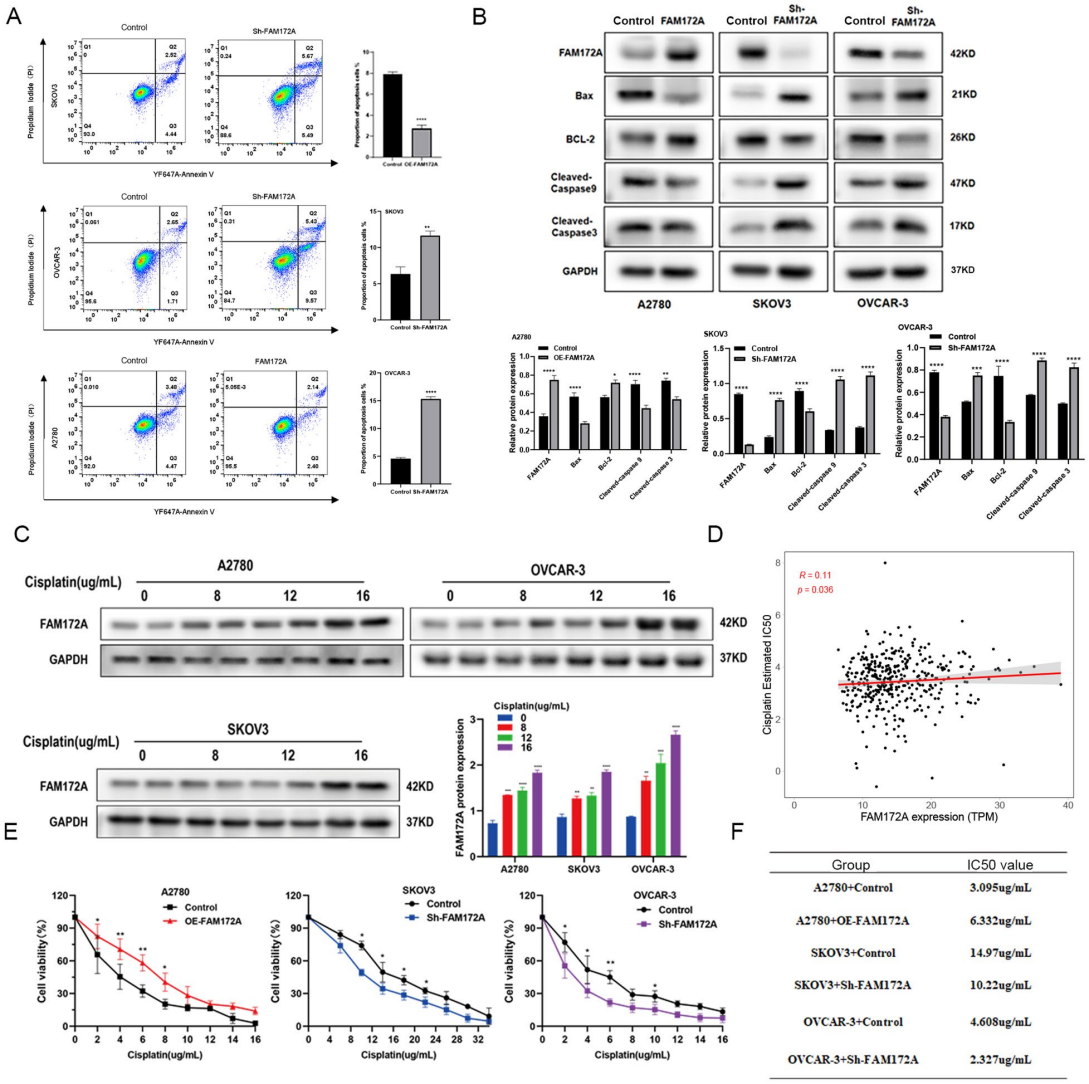
Role of FAM172A in apoptosis and cisplatin resistance of EOC cells. (**A**) Flow cytometry analysis of apoptosis in EOC cells after FAM172A upregulation or knockdown. (**B**) Western blot analysis of apoptosis-related proteins. (**C**) Correlation between FAM172A expression and cisplatin IC₅₀ values. (**D**) Western blot analysis of FAM172A expression in A2780, SKOV3, and OVCAR-3 cells treated with increasing concentrations of cisplatin. (**E**) Cell viability measured by CCK-8 assay in EOC cells with modulated FAM172A expression under cisplatin treatment. (**F**) IC₅₀ values of cisplatin across different treatment groups. *P < 0.05, **P < 0.01, ***P < 0.001, ****P < 0.0001.

Overexpression of FAM172A in A2780 cells reduced apoptosis, while knockdown of FAM172A in SKOV3 and OVCAR-3 cells increased apoptosis (Fig. 2A). In agreement with these findings, overexpression of FAM172A in A2780 cells decreased the levels of Bax, cleaved caspase-9, and cleaved caspase-3, while increasing the expression of Bcl-2. In contrast, FAM172A knockdown in SKOV3 and OVCAR-3 cell upregulated Bax, cleaved caspase-9, and cleaved caspase-3, while downregulated Bcl-2 (Fig. 2B). These results indicate that FAM172A suppresses apoptosis in EOC cells by regulating key apoptotic proteins.

### FAM172A modulates cisplatin sensitivity in EOC cells

To evaluate the clinical relevance of FAM172A in chemoresistance, we analyzed its correlation with cisplatin sensitivity using transcriptomic data from the TCGA-OV dataset. The half-maximal inhibitory concentration (IC₅₀) of cisplatin was predicted via the pRRophetic R package. Spearman correlation analysis indicated a positive relationship between FAM172A expression and cisplatin IC₅₀, implying that patients with lower FAM172A levels might experience better drug efficacy (Fig. 2C). Experimentally, treating A2780, SKOV3, and OVCAR-3 cells with escalating doses of cisplatin resulted in a concurrent increase in FAM172A protein levels (Fig. 2D). Functional studies confirmed that FAM172A overexpression in A2780 cells significantly raised the IC₅₀ value, denoting reduced cisplatin sensitivity. Conversely, FAM172A knockdown in SKOV3 and OVCAR-3 cells substantially lowered the IC₅₀ value, indicating heightened drug sensitivity (Fig. 2E–F). These data collectively demonstrate that FAM172A negatively regulates cisplatin sensitivity in EOC cells.

### FAM172A knockdown suppresses EOC tumor growth in vivo

**Fig. 3:**
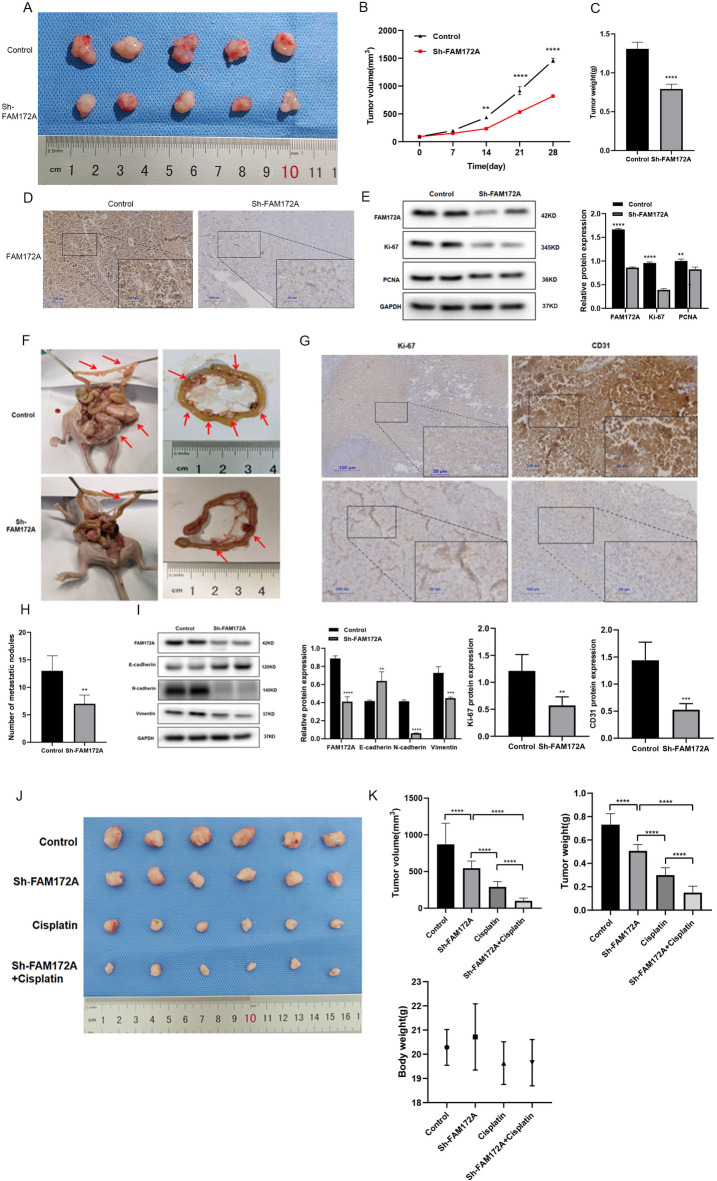
FAM172A knockdown inhibits EOC tumor growth in vivo. (**A**) Representative images of excised tumors. (**B**, **C**) Tumor volume (**B**) and weight (**C**) in BALB/c nude mice. (**D**) Immunohistochemical (IHC) staining of FAM172A expression in xenograft tissues. (**E**) Western blot analysis of Ki-67 and PCNA levels in subcutaneous tumors. (**F**) Gross morphology of peritoneal metastases in nude mice. (**G**) H&E staining of metastatic nodules. (**H**) Number of peritoneal metastatic nodules. (**I**) Western blot analysis of epithelial–mesenchymal transition (EMT)-related proteins after FAM172A silencing. (**J**) Representative tumor images from each experimental group. (**K**) Tumor growth curves, final tumor weight, and mouse body weight per group. *P < 0.05, **P < 0.01, ***P < 0.001, ****P < 0.0001.

Mouse xenograft models demonstrated that FAM172A knockdown significantly suppresses tumor growth in vivo. Tumors derived from FAM172A-knockdown cells were markedly smaller size (Fig. 3A). exhibited a slower growth rate, and had lower final weights compared to controls (Fig. 3B-C). Immunohistochemistry confirmed reduced FAM172A expression in the shFAM172A group (Fig. 3D). Furthermore, staining for the proliferation markers Ki-67 and PCNA revealed substantially decreased proliferative activity in knockdown tumors relative to the shCtrl group (Fig. 3E).

### FAM172A knockdown suppresses EOC cell metastasis in vivo

Peritoneal metastasis mouse models were established to investigate the role of FAM172A in EOC metastasis. Knockdown of FAM172A significantly reduced the number of mesenteric metastatic nodules in the intestinal wall of nude mice (Fig. 3F, H). Immunohistochemical staining showed that FAM172A knockdown also decreased the expression of Ki‑67 and CD31, markers of cell proliferation and angiogenesis respectively (Fig. 3G), indicating suppressed tumor growth and angiogenesis in vivo. Furthermore, FAM172A knockdown reduced the expression of N‑cadherin and vimentin while increasing E‑cadherin (Fig. 3I). These results demonstrate that FAM172A promotes peritoneal metastasis in EOC by enhancing proliferation, angiogenesis, and epithelial–mesenchymal transition.

### Combined cisplatin treatment and FAM172A knockdown suppress EOC tumor growth

Tumor size was reduced in the shFAM172A group and the cisplatin-treated group compared to the control group. The combination of cisplatin and FAM172A knockdown resulted in significantly greater tumor growth inhibition than either treatment alone (Fig. 3J). Body weights of nude mice showed no significant differences across the four groups during the experiment (Fig. 3K), indicating that the cisplatin dosage was safe and well tolerated. These findings suggest that silencing FAM172A enhances the sensitivity of EOC cells to cisplatin in vivo without observable systemic toxicity.

### FAM172A regulates the PI3K–Akt signaling pathway in EOC

**Fig. 4:**
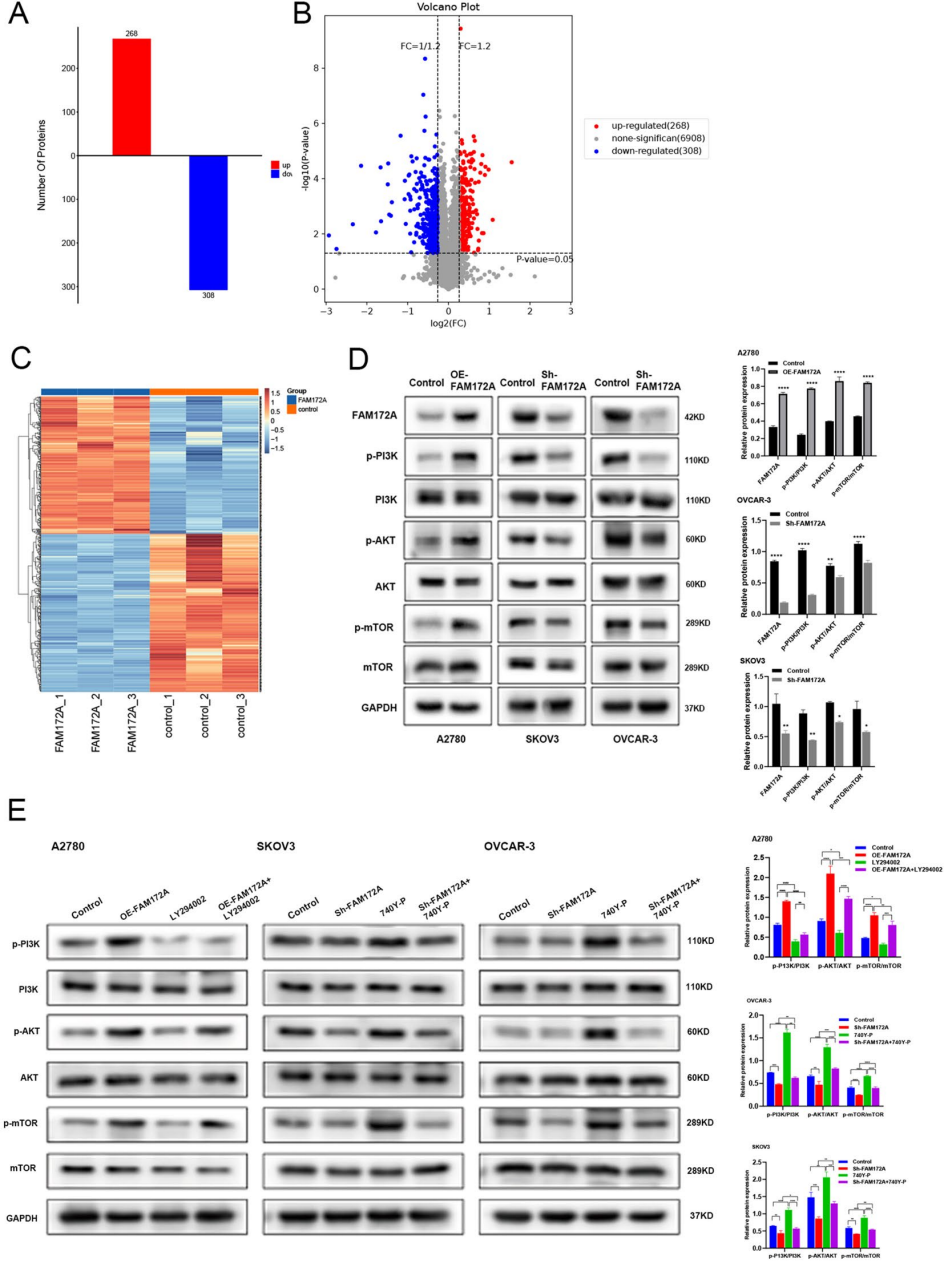
FAM172A modulates biological functions via activation of the PI3K/Akt signaling pathway. (**A**) Proteomic analysis of differentially expressed proteins following FAM172A modulation. (**B**, **C**) Volcano plot (**B**) and cluster analysis (**C**) of differentially expressed proteins. (**D**, **E**) Western blot analysis of key proteins in the PI3K/Akt pathway under different FAM172A expression conditions. *P < 0.05, **P < 0.01, ***P < 0.001, ****P < 0.0001.

To further investigate the role of FAM172A in EOC, we performed proteomic analysis comparing FAM172A-overexpressing and control A2780 cells. A total of 576 differentially expressed proteins (DEPs) were identified, including 308 downregulated and 268 upregulated DEPs (Fig. 4A–C). Classical pathway analysis suggested FAM172A involvement in the PI3K–Akt signaling pathway (Fig. S4), which was confirmed by western blot after FAM172A knockdown and overexpression. FAM172A overexpression increased phosphorylation of PI3K, Akt, and mTOR, while knockdown decreased their phosphorylation (Fig. 4D). Additional experiments using the PI3K inhibitor LY294002 and activator 740Y‑P verified that FAM172A acts through activation of the PI3K/Akt pathway (Fig. 4E). These results demonstrate that FAM172A regulates the PI3K–Akt signaling pathway in EOC.

### FAM172A promotes EOC cell proliferation via the PI3K/AKT signaling pathway

**Fig. 5:**
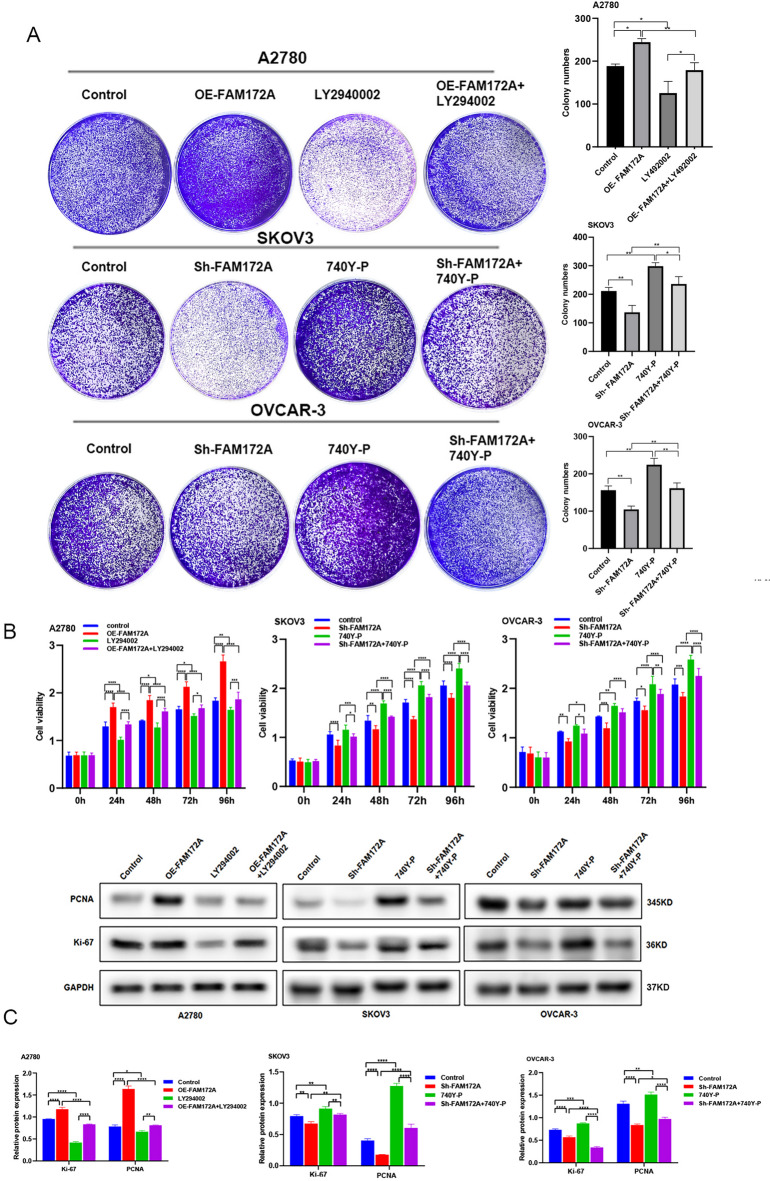
FAM172A regulates EOC cell proliferation through the PI3K/AKT signaling pathway. (**A**, **B**) Colony formation assay (**A**) and CCK-8 assay (**B**) evaluating the effect of PI3K activator (740Y-P) and inhibitor (LY294002) on FAM172A-induced proliferation. (**C**) Western blot analysis of proliferation-related proteins (PCNA, KI67). *P < 0.05, **P < 0.01, ***P < 0.001, ****P < 0.0001.

We examined the effects of the PI3K inhibitor LY294002 and activator 740Y‑P on FAM172A‑mediated EOC cell proliferation. LY294002 significantly suppressed cell proliferation, while 740Y‑P strongly enhanced it. Notably, LY294002 effectively reversed the pro‑proliferative effect of FAM172A overexpression, and 740Y‑P largely rescued the anti‑proliferative effect of FAM172A knockdown (Fig. 5A‑B). Furthermore, LY294002 markedly reduced PCNA and Ki‑67 expression, whereas 740Y‑P increased their levels (Fig. 5C). Both LY294002 and 740Y‑P attenuated the respective effects of FAM172A overexpression or knockdown on PCNA and Ki‑67 expression (Fig. 5C). Together, these results demonstrate that the PI3K/AKT-pathway is essential for FAM172A‑induced proliferation in EOC cells.

### FAM172A promotes EOC cell migration and invasion through the PI3K/AKT pathway

**Fig. 6:**
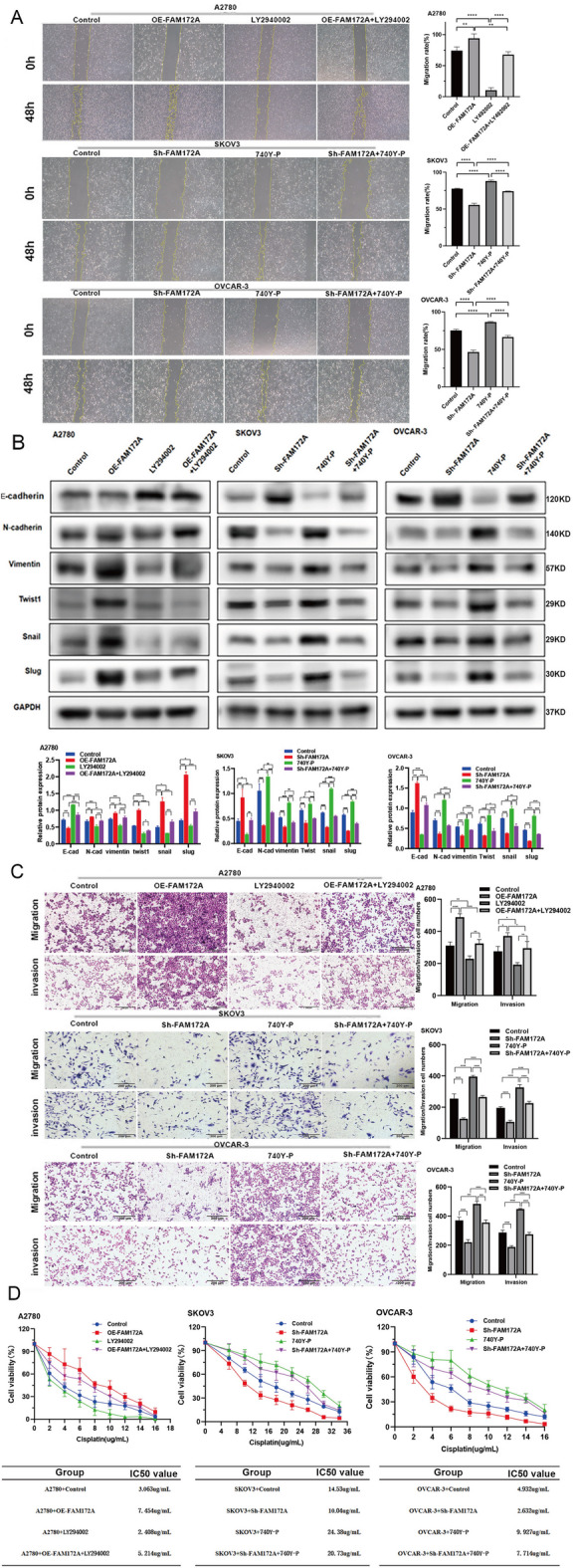
The PI3K/AKT-pathway mediates FAM172A effects on migration, invasion, and cisplatin resistance in EOC cells. (**A**, **C**) Wound healing assay (**A**) and Transwell assay. (**B**) Western blot analysis of epithelial–mesenchymal transition (EMT)-related proteins. (**C**) assessing the influence of PI3K activator and inhibitor on FAM172A-induced migration and invasion. (**D**) Effect of PI3K modulator treatment on FAM172A-mediated cisplatin resistance (IC₅₀). *P* < 0.05, **P < 0.01, ***P < 0.001, ****P < 0.0001.

We next examined the effects of the PI3K inhibitor LY294002 and activator 740Y‑P on FAM172A‑mediated migration and invasion in EOC cells. LY294002 significantly reduced cell migration and invasion, while 740Y‑P strongly enhanced these abilities. Notably, LY294002 effectively reversed the pro‑migratory and pro‑invasive effects of FAM172A overexpression, and 740Y‑P counteracted the suppressive effects of FAM172A knockdown (Fig. 6A, C). Furthermore, LY294002 or 740Y‑P attenuated the respective regulatory effects of FAM172A overexpression or knockdown on the expression of N‑cadherin, vimentin, Twist1, Snail, Slug, and E‑cadherin (Fig. 6B). These results indicate that PI3K/AKT-pathway activation is critical for FAM172A‑induced migration and invasion in EOC cells.

### FAM172A modulates cisplatin resistance in EOC cells via the PI3K/AKT-pathway

To determine whether FAM172A regulates cisplatin resistance through the PI3K/AKT-pathway, we treated EOC cells with the PI3K inhibitor LY294002 or activator 740Y‑P and measured cisplatin sensitivity by CCK‑8 assay. LY294002 significantly decreased the IC₅₀ value, indicating enhanced cisplatin sensitivity, while 740Y‑P increased the IC₅₀, indicating reduced sensitivity (Fig. 6D). Furthermore, LY294002 counteracted the increase in IC₅₀ induced by FAM172A overexpression, and 740Y‑P reversed the decrease in IC₅₀ caused by FAM172A knockdown (Fig. 6D). These results demonstrate that FAM172A regulates cisplatin resistance in EOC cells through the PI3K/AKT signaling pathway.

## Discussion

This study identified elevated FAM172A expression in EOC tissues and cell lines. Functional experiments demonstrated that FAM172A overexpression enhanced EOC cell proliferation and metastasis in vitro and in vivo, whereas its silencing suppressed these malignant phenotypes. FAM172A also contributed to cisplatin resistance in EOC cells. Mechanistically, FAM172A promoted EOC progression by activating the PI3K/AKT signaling pathway. These findings collectively underscore the significance of FAM172A in driving EOC progression and chemoresistance via PI3K/AKT activation.

The functional role of FAM172A appears context-dependent across cancer types. While it acts as an oncogene in certain malignancies, it may serve a tumor-suppressive function in others. Previous studies reported high FAM172A expression in follicular thyroid carcinoma^16^ and colorectal cancer^15^. In colorectal cancer, FAM172A promotes cell proliferation and invasion, suggesting potential as a therapeutic target^17^. In contrast, FAM172A inhibits epithelial–mesenchymal transition (EMT) through the ERK–MAPK pathway in pancreatic cancer, indicating a tumor-suppressor role in this context^18^.

Consistent with findings by Chen et al. that FAM172A overexpression enhances proliferation and migration in Movas cells^19^, our results showed that FAM172A upregulation promotes proliferation, migration, invasion, cell cycle progression, and apoptosis resistance in EOC, supported by corresponding protein expression changes. Conversely, FAM172A knockdown suppressed malignant behavior. Prior in vivo studies also indicated that FAM172A downregulation inhibits tumor growth in follicular thyroid cancer models^10^. Similarly, we observed that FAM172A knockdown reduced tumor growth, metastatic nodule formation, and modulated proliferation and invasion-related protein expression in EOC, supporting its potential as a therapeutic target to suppress EOC progression and metastasis.

Cisplatin, a platinum-based chemotherapeutic agent, is widely used in ovarian cancer treatment due to its efficacy^20,21^. However, the development of cisplatin resistance remains a major clinical challenge^22^. Combination therapies targeting DNA repair pathways, modulating apoptosis, or counteracting tumor microenvironment-mediated resistance offer promising strategies to enhance cisplatin effectiveness^23,24^. Our study indicates that FAM172A contributes to cisplatin sensitivity, as its overexpression correlates with reduced treatment response. Reported IC₅₀ values for cisplatin vary across studies: approximately 3.7 µM and 13.8 µM in OVCAR-3 and SKOV-3, respectively^25^; 17.4 µM (OVCAR-3) and 25.7 µM (SKOV-3) in Smith et al.^26^; 3.12 µM (A2780) and 12.53 µM (SKOV-3) in Gong et al.^27^; and Han et al. showed that 16 µM cisplatin allowed > 40% survival in SKOV-3 after 48 h, supporting the concept of relative resistance^28^. Importantly, combining cisplatin with FAM172A knockdown synergistically suppressed EOC tumor proliferation, suggesting a promising therapeutic strategy for improving patient outcomes.

The PI3K-Akt signaling pathway is a critical intracellular cascade implicated in the pathogenesis of multiple cancers and is a well-established regulator of cell growth and cell cycle progression^29^. In this study, FAM172A overexpression accelerated G1/S phase transition, while its knockdown induced G1 arrest, accompanied by increased cyclin D1, CDK4, and CDK6 expression and decreased p21, p27, and p53 levels—key regulators of the G1/S checkpoint. Previous work showed that FAM172A isoforms can activate the PI3K/AKT-pathway in HepG2 cells^30^. Our results further revealed that FAM172A activates PI3K/AKT signaling, increasing AKT phosphorylation, and that PI3K inhibition with LY294002 suppressed FAM172A-driven proliferation. Thus, FAM172A appears to promote EOC cell proliferation at least partly through PI3K/AKT-mediated acceleration of G1/S transition. The PI3K/AKT-pathway also regulates cancer progression and drug resistance in ovarian cancer^31,32^, where its aberrant activation promotes tumor growth, metastasis, and chemoresistance^33,34^. Importantly, modulation of PI3K signaling, through either inhibition or activation, mediated the effects of FAM172A on EOC cell proliferation, migration, invasion, and cisplatin resistance. These findings underscore the significance of FAM172A-driven oncogenesis in EOC via the PI3K-Akt pathway.

Angiogenesis, the formation of new blood vessels, is critical for tumor growth and metastasis and is frequently driven by upregulation of pro-angiogenic factors such as vascular endothelial growth factor (VEGF)^35^. VEGF promotes endothelial cell proliferation, migration, and tube formation, thereby supporting tumor vascularization^36^, and its overexpression is strongly associated with poor prognosis and chemoresistance in EOC^37^. Our in vivo experiments showed that FAM172A knockdown significantly reduced CD31-positive microvessel density in xenograft tumors, indicating impaired angiogenesis and suggesting that FAM172A contributes to EOC progression partly by facilitating tumor vascularization. Since the PI3K/AKT pathway can regulate VEGFA and influence tumor angiogenesis^38^, and VEGF promotes ovarian cancer progression and platinum resistance^39^, FAM172A-mediated PI3K/AKT activation may indirectly affect angiogenesis and treatment response. However, VEGF expression and endothelial cell function were not directly assessed here, and further studies are needed to validate whether FAM172A regulates VEGF via PI3K/AKT.

Our findings suggest FAM172A expression in tumor tissues may serve as a prognostic biomarker in EOC, though the current study did not evaluate FAM172A levels in blood or perform longitudinal monitoring. The potential for measuring FAM172A in blood or serially during treatment represents a prospective application requiring future investigation. If validated, such approaches could aid in risk stratification, early progression detection, or treatment response assessment, though optimal timing and detection methods for liquid biopsy-based FAM172A measurement remain to be established in clinical cohorts.

In conclusion, this study elucidates the multifaceted roles of FAM172A in EOC progression and highlights its potential as a prognostic marker and therapeutic target. These findings underscore the importance of targeting the PI3K-Akt pathway in FAM172A-mediated oncogenesis and offer new insights for developing effective EOC treatment strategies.

## Supplementary Information

Below is the link to the electronic supplementary material.

Supplementary Material 1

Supplementary Material 2

Supplementary Material 3

Supplementary Material 4

Supplementary Material 5

Supplementary Material 6

Supplementary Material 7

## Data Availability

All data generated or analyzed during this study are included in this article.

## References

[1] Chiappa, M. et al. Brca1Combinations of ATR, Chk1 and Wee1 Inhibitors with Olaparib Are Active in Olaparib Resistant Proficient and Deficient Murine Ovarian Cells. *Cancers*. **14** (2022). 10.3390/cancers14071807 PMC8997432 35406579

[2] He, M., Lai, Y., Peng, H. & Tong, C. Role of lymphadenectomy during interval debulking surgery performed after neoadjuvant chemotherapy in patients with advanced ovarian cancer. *Front. Oncol.* **11**, 646135 (2021). 33842358 10.3389/fonc.2021.646135 PMC8034395

[3] Rabelo-Fernández, R. J. et al. Reduced RBPMS levels promote cell proliferation and decrease cisplatin sensitivity in ovarian cancer cells. *Int J. Mol. Sci* **23**, 535 (2022). 10.3390/ijms23010535 PMC8745614 35008958

[4] Hwangbo, S. et al. Development of machine learning models to predict platinum sensitivity of High-Grade serous ovarian carcinoma. *Cancers (Basel).* **13**, 1875 (2021). 10.3390/cancers13081875 PMC8070756 33919797

[5] Fumet, J. D. et al. Genomic instability is defined by specific tumor microenvironment in ovarian cancer: A subgroup analysis of AGO OVAR 12 trial. *Cancers (Basel).* **14**, 1189 (2022). 10.3390/cancers14051189 PMC8909387 35267497

[6] Miyakawa, R. et al. SPON1 is an independent prognostic biomarker for ovarian cancer. *J. Ovarian Res.* **16**, 95 (2023). 37179355 10.1186/s13048-023-01180-8 PMC10182672

[7] Rocconi, R. P. et al. Gemogenovatucel-T (Vigil) immunotherapy as maintenance in frontline stage III/IV ovarian cancer (VITAL): a randomised, double-blind, placebo-controlled, phase 2b trial. *Lancet Oncol.* **21**, 1661–1672 (2020). 33271095 10.1016/S1470-2045(20)30533-7

[8] Liu, W. et al. Expression of family with sequence similarity 172 member A and nucleotide-binding protein 1 is associated with the poor prognosis of colorectal carcinoma. *Oncol. Lett.* **14**, 3587–3593 (2017). 28927116 10.3892/ol.2017.6585 PMC5588006

[9] Bélanger, C. et al. Dysregulation of cotranscriptional alternative splicing underlies CHARGE syndrome. *Proc. Natl. Acad. Sci. U.S.A.* **115**, E620–E629 (2018). 29311329 10.1073/pnas.1715378115 PMC5789929

[10] Xu, P. et al. FAM172A promotes follicular thyroid carcinogenesis and May be a marker of FTC. *Endocr. Relat. Cancer*. **27**, 657–669 (2020). 33095186 10.1530/ERC-20-0181 PMC7707803

[11] Li, M. et al. FAM172A protein promotes the proliferation of human papillary thyroid carcinoma cells via the p38 mitogen-activated protein kinase pathway. *Mol. Med. Rep.* **13**, 353–358 (2016). 26573560 10.3892/mmr.2015.4548

[12] Xu, W. et al. FAM171A2 The gene is a key regulator of progranulin expression and modifies the risk of multiple neurodegenerative diseases. *Science Advances.* **6**, eabb3063 (2020). 10.1126/sciadv.abb3063 PMC7577723 33087363

[13] Larsen, M. et al. Objective and subjective measures of sleep initiation are differentially associated with DNA methylation in adolescents. *Clin. Epigenetics*. **15**, 136 (2023). 37634000 10.1186/s13148-023-01553-2 PMC10464279

[14] Guo, L. et al. Epigenome-wide DNA methylation analysis of whole blood cells derived from patients with GAD and OCD in the Chinese Han population. *Translational Psychiatry*. **12**, 465 (2022). 36344488 10.1038/s41398-022-02236-x PMC9640561

[15] Xu, A. M., He, C. J., Tuerxun, Z. & Anikezi, A. FAM172A affects cell proliferation and apoptosis not by targeting β-tubulin in HepG2 cells. *Transl Cancer Res.* **9**, 5637–5644 (2020). 35117927 10.21037/tcr-20-2868 PMC8797783

[16] Xu, P. P. et al. FAM172A promotes follicular thyroid carcinogenesis and May be a marker of FTC. *Endocr. Relat. Cancer*. **27**, 657–669 (2020). 33095186 10.1530/ERC-20-0181 PMC7707803

[17] Liu, W. et al. [Corrigendum] miR–27a promotes proliferation, migration, and invasion of colorectal cancer by targeting FAM172A and acts as a diagnostic and prognostic biomarker. *Oncol Rep.* **51**, 83 (2024). 10.3892/or.2024.8742 PMC11063750 38666532

[18] Chen, Y. et al. FAM172A inhibits EMT in pancreatic cancer via ERK-MAPK signaling. *Biol Open.* **9**, bio048462 (2020). 10.1242/bio.048462 PMC7044457 31988090

[19] Chen, M. et al. Deletion of Fam172a accelerates advanced atherosclerosis and induces plaque instability. *Atherosclerosis* **333**, 39–47 (2021). 34425526 10.1016/j.atherosclerosis.2021.08.023

[20] Zha, M. et al. The circadian clock gene Bmal1 facilitates cisplatin-induced renal injury and hepatization. *Cell. Death Dis.* **11**, 446 (2020). 32522976 10.1038/s41419-020-2655-1 PMC7287064

[21] Salatino, A. et al. H-Ferritin affects Cisplatin-Induced cytotoxicity in ovarian cancer cells through the modulation of ROS. *Oxid. Med. Cell. Longev.* **2019**, 3461251 (2019). 31781333 10.1155/2019/3461251 PMC6875340

[22] Cheng, M. et al. The mitochondrial PHB2/OMA1/DELE1 pathway cooperates with Endoplasmic reticulum stress to facilitate the response to chemotherapeutics in ovarian cancer. *Int J. Mol. Sci.* **23**, 1320 (2022). 10.3390/ijms23031320 PMC8835964 35163244

[23] Kulshrestha, A. et al. Selective Inhibition of tumor cell associated Vacuolar-ATPase ‘a2’ isoform overcomes cisplatin resistance in ovarian cancer cells. *Mol. Oncol.* **10**, 789–805 (2016). 26899534 10.1016/j.molonc.2016.01.003 PMC5423172

[24] Zhang, F. et al. Simultaneous targeting of ATM and Mcl-1 increases cisplatin sensitivity of cisplatin-resistant non-small cell lung cancer. *Cancer Biol. Ther.* **18**, 606–615 (2017). 28686074 10.1080/15384047.2017.1345391 PMC5653185

[25] Fuster, V., Cohen, M. & Chesebro, J. H. Usefulness of aspirin for coronary artery disease. *Am. J. Cardiol.* **61**, 637–640 (1988). 3278582 10.1016/0002-9149(88)90780-1

[26] Smith, J. A., Ngo, H., Martin, M. C. & Wolf, J. K. An evaluation of cytotoxicity of the taxane and platinum agents combination treatment in a panel of human ovarian carcinoma cell lines. *Gynecol. Oncol.* **98**, 141–145 (2005). 15963813 10.1016/j.ygyno.2005.02.006

[27] Hall, J. P. J. Is the bacterial chromosome a mobile genetic element? *Nat. Commun.* **12**, 6400 (2021). 34737310 10.1038/s41467-021-26758-y PMC8568949

[28] Aydemirli, M. D. et al. Targeting EML4-ALK gene fusion variant 3 in thyroid cancer. *Endocr. Relat. Cancer*. **28**, 377–389 (2021). 33878728 10.1530/ERC-20-0436 PMC8183637

[29] Antonino G, et al. PI3K/AKT/mTOR signaling transduction pathway and targeted therapies in cancer. *Mol Cancer*. **22**, 138 (2023). 10.1186/s12943-023-01827-6 PMC10436543 37596643

[30] Zhao, H. et al. The Effect of Protein FAM172A on Proliferation in HepG2 Cells and Investigation of the Possible Molecular Mechanism. *Analytical cellular pathology (Amsterdam)*. 5901083 (2019). 10.1155/2019/5901083 PMC6930761 31915594

[31] Ma, S. et al. HIF-2α-dependent TGFBI promotes ovarian cancer chemoresistance by activating PI3K/Akt pathway to inhibit apoptosis and facilitate DNA repair process. *Sci. Rep.* **14**, 3870 (2024). 38365849 10.1038/s41598-024-53854-y PMC10873328

[32] Wang, H. et al. β-Sitosterol targets ASS1 for Nrf2 ubiquitin-dependent degradation, inducing ROS-mediated apoptosis via the PTEN/PI3K/AKT signaling pathway in ovarian cancer. *Free Radic. Biol. Med.* **214**, 137–157 (2024). 38364944 10.1016/j.freeradbiomed.2024.02.004

[33] Parashar, D. et al. Patient-Derived ovarian cancer spheroids rely on PI3K-AKT signaling addiction for cancer stemness and chemoresistance. *Cancers (Basel).* **14**, 958 (2022). 10.3390/cancers14040958 PMC8870411 35205706

[34] Zhang, J. et al. Current landscape of personalized clinical treatments for triple-negative breast cancer. *Front. Pharmacol.* **13**, 977660 (2022). 36188535 10.3389/fphar.2022.977660 PMC9523914

[35] Siveen, K. S. et al. Vascular endothelial growth factor (VEGF) signaling in tumour vascularization: potential and challenges. *Curr. Vasc Pharmacol.* **15**, 339–351 (2017). 28056756 10.2174/1570161115666170105124038

[36] Niu, H., Gao, N., Dang, Y., Guan, Y. & Guan, J. Delivery of VEGF and delta-like 4 to synergistically regenerate capillaries and arterioles in ischemic limbs. *Acta Biomater.* **143**, 295–309 (2022). 35301145 10.1016/j.actbio.2022.03.021 PMC9926495

[37] Ma, M. & Yu, N. Over-Expression of TBL1XR1 indicates poor prognosis of serous epithelial ovarian cancer. *Tohoku J. Exp. Med.* **241**, 239–247 (2017). 28344213 10.1620/tjem.241.239

[38] Yuwei S, et al. Exosomal miR-301a-3p from esophageal squamous cell carcinoma cells promotes angiogenesis by inducing M2 polarization of macrophages via the PTEN/PI3K/AKT signaling pathway. *Cancer Cell Int*. **22**, 153 (2022). 10.1186/s12935-022-02570-6 PMC9014619 35436935

[39] Danxue H, Liyuan K, Hongxia C, Su L, Feilong SJBWH. Efficacy and safety of VEGF/VEGFR inhibitors for platinum-resistant ovarian cancer: a systematic review and meta-analysis of randomized controlled trials. *BMC Womens Health*. **24**, 34 (2024). 10.1186/s12905-023-02879-y PMC10788010 38218775

