# Supplementary figures and images for: FAM172A promotes epithelial ovarian cancer progression and induces platinum resistance via the PI3K/AKT pathway
Source: Sci Rep. 2025 Dec 3;15:43128. doi: 10.1038/s41598-025-26676-9 (PMC12678607; doi:10.1038/s41598-025-26676-9)

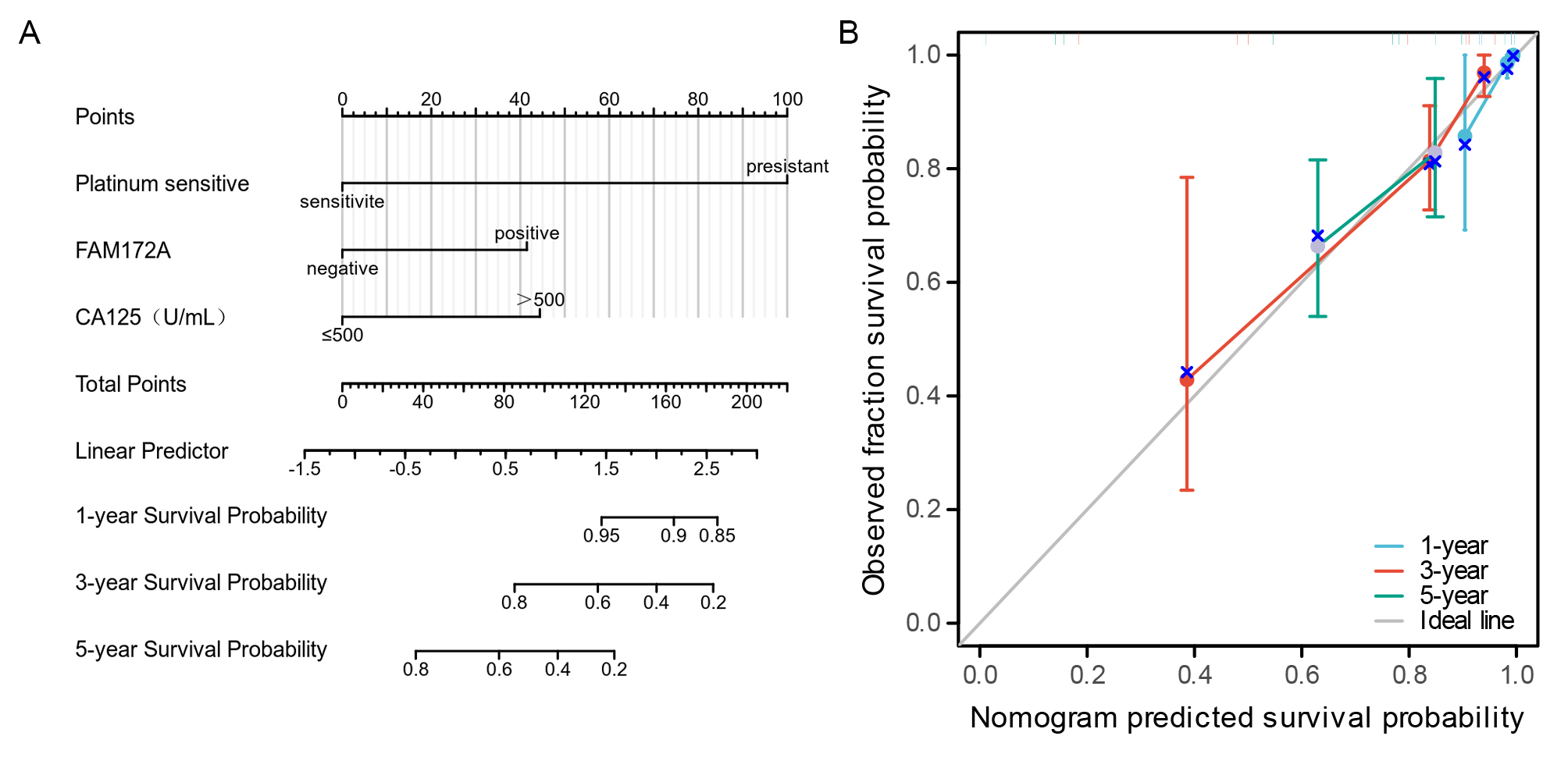

Supplement: Supplementary file 1 — Supplementary Material 1 [file 41598_2025_26676_MOESM1_ESM.jpg]

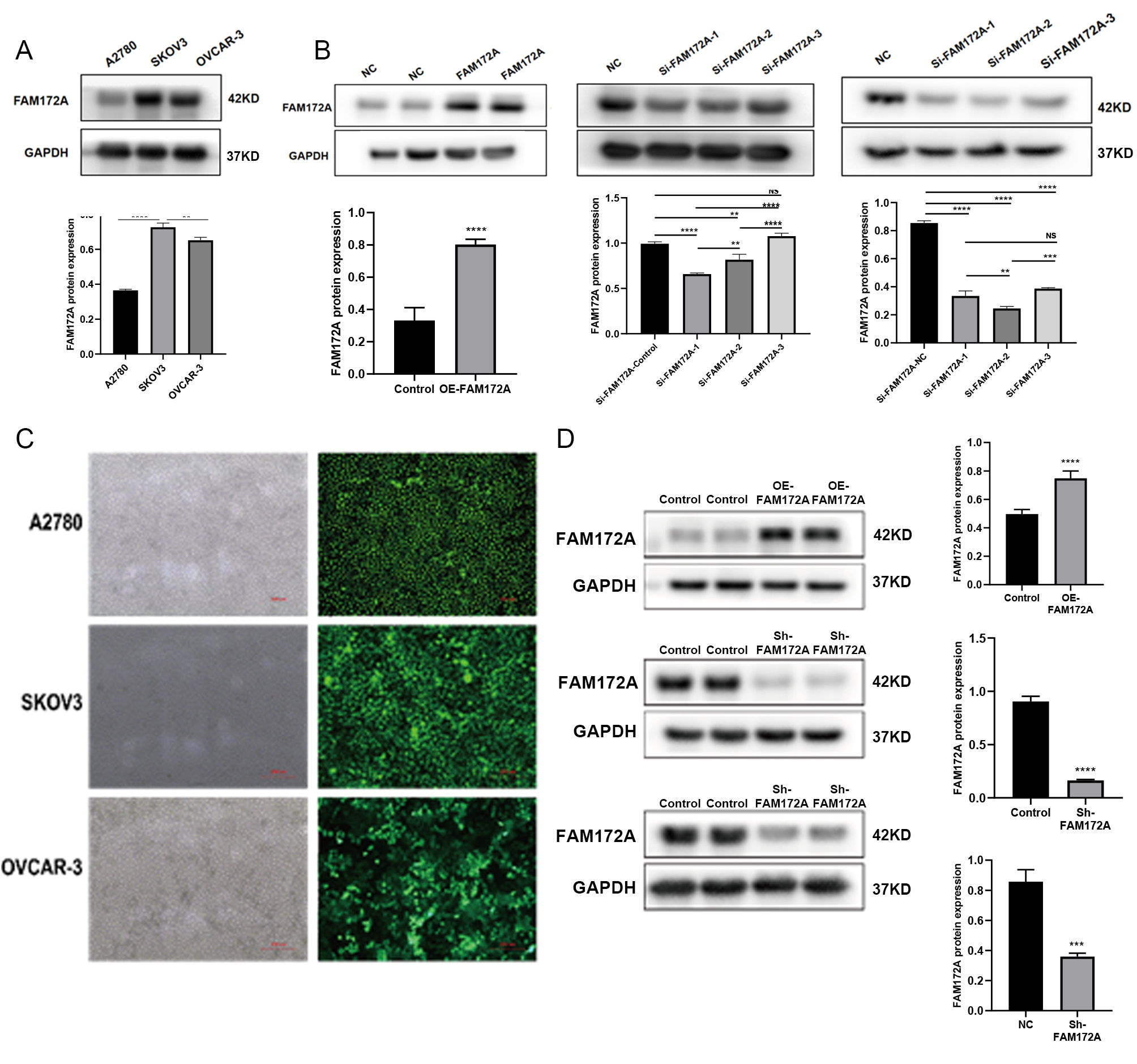

Supplement: Supplementary file 2 — Supplementary Material 2 [file 41598_2025_26676_MOESM2_ESM.jpg]

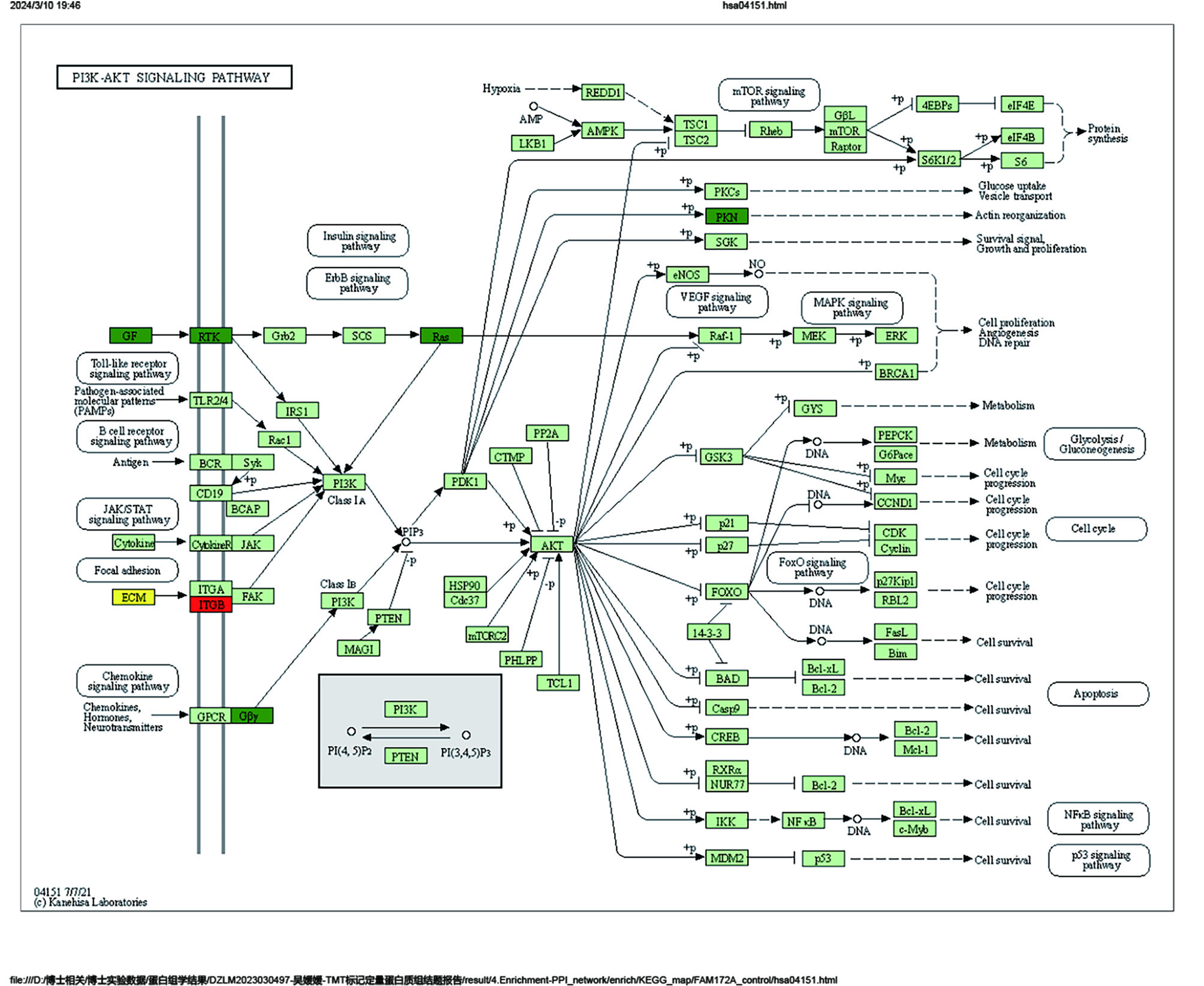

Supplement: Supplementary file 4 — Supplementary Material 4 [file 41598_2025_26676_MOESM4_ESM.tif]

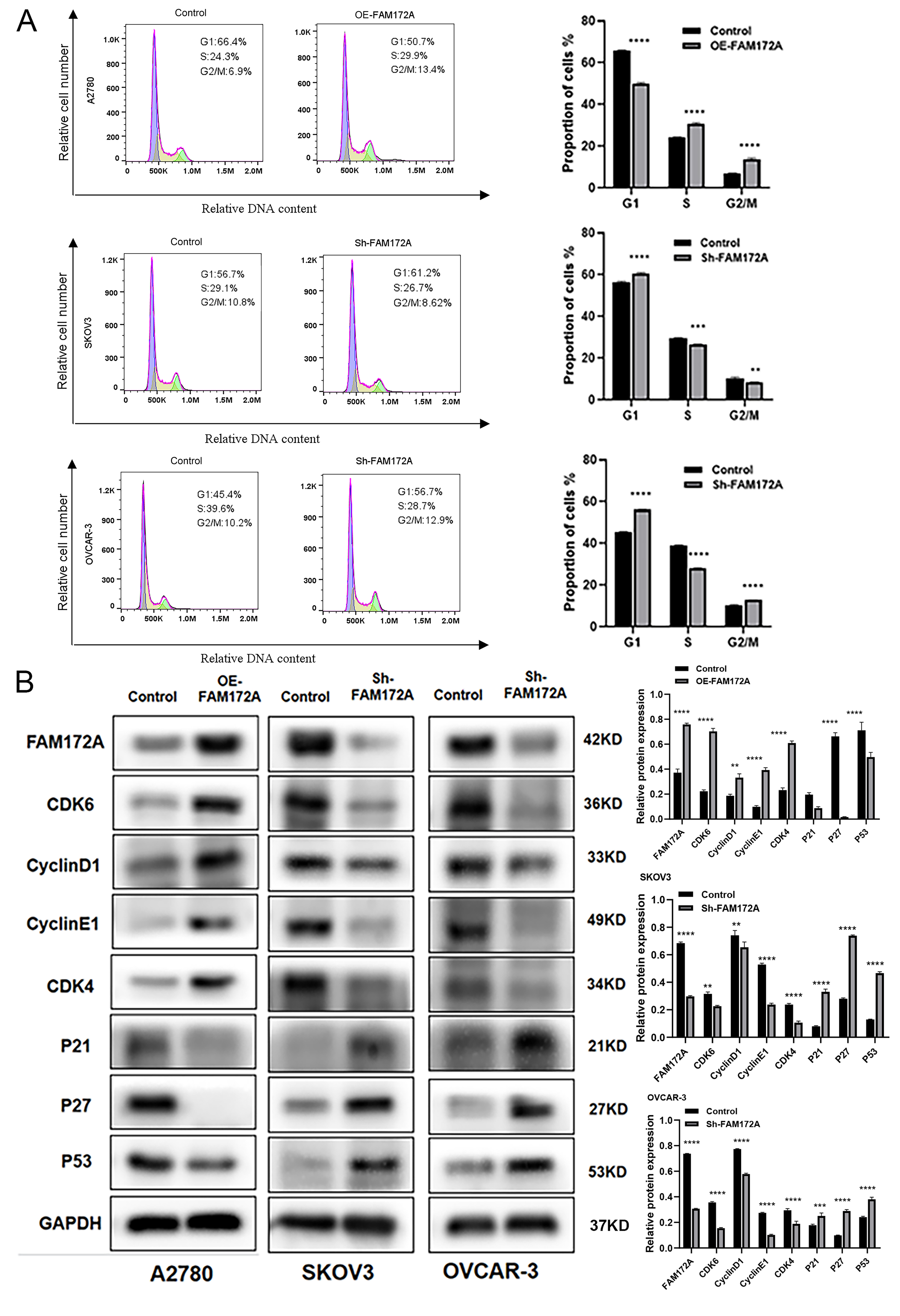

Supplement: Supplementary file 5 — Supplementary Material 5 [file 41598_2025_26676_MOESM5_ESM.tif]
