# Supplementary material for: FAM172A promotes epithelial ovarian cancer progression and induces platinum resistance via the PI3K/AKT pathway
Source: Sci Rep. 2025 Dec 3;15:43128. doi: 10.1038/s41598-025-26676-9 (PMC12678607; doi:10.1038/s41598-025-26676-9)
Supplement: Supplementary file 3 — Supplementary Material 3 [file 41598_2025_26676_MOESM3_ESM.docx]

**Supplementary materials**

**Figure S1. Construction of a nomogram model for predicting survival in EOC patients**

A, Nomogram plot illustrating the prognostic prediction for EOC patients. B, Calibration plot for predicted overall survival rate**.**

**Figure S2. Construction of overexpressing, silenced FAM172A, and stable transfected cell lines in EOC cell lines.**

A, Expression levels of FAM172A in EOC cell lines A2780, SKOV3, and OVCAR-3. B, Validation of FAM172A overexpression in A2780 and silencing of FAM172A in SKOV3 and OVCAR-3. C, The expression of FAM172A in EOC cell line was characterized by immunofluorescence. D, Verification of stable transfected cell lines overexpressing or silencing FAM172A in EOC cell lines. *P<0.05, **P<0.01, ***P<0.001, ****P<0.0001.

**Figure S3. Impact of FAM172A overexpression and silencing on cell cycle progression of EOC cells**

A, Flow cytometry analysis of the effect of FAM172A overexpression and silencing on the cell cycle modulation in EOC cells. B, Western blot analysis depicting the influence of FAM172A on cell cycle-related proteins. *P<0.05, **P<0.01, ***P<0.001, ****P<0.0001.

**Figure S4. The proteomic findings indicate that FAM172A modulates biological processes through the activation of the PI3K/AKT signaling pathway.**

**Table S1. Correlation between clinical and pathological features, FAM172A expression and platinum resistance in patients.**

**Table S2. Univariate and multivariate COX regression analysis of survival factors in patients with ovarian epithelial cancer.**

**Table S3. Forest plot of factors affecting survival of patients with ovarian epithelial cancer.**
