# Supplementary material for: FAM172A promotes epithelial ovarian cancer progression and induces platinum resistance via the PI3K/AKT pathway
Source: Sci Rep. 2025 Dec 3;15:43128. doi: 10.1038/s41598-025-26676-9 (PMC12678607; doi:10.1038/s41598-025-26676-9)

Figure 1B

FAM172A _pub in the first figures

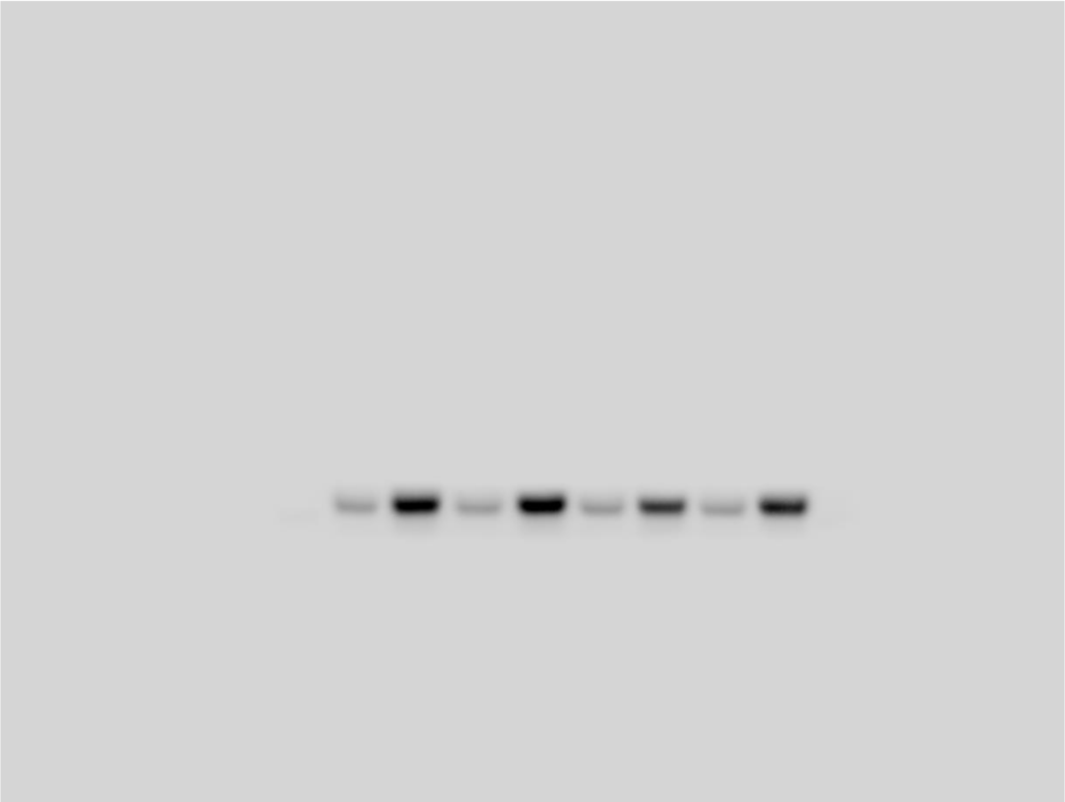

FAM172A _pub in the second figures

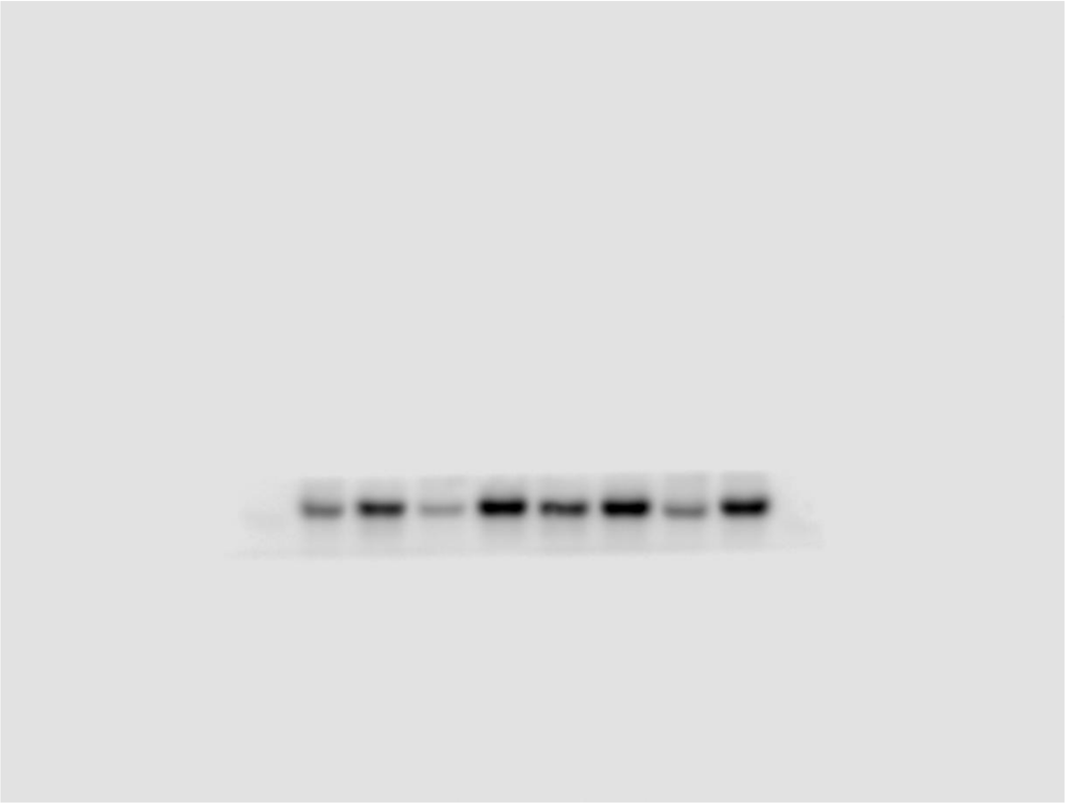

GAPDH_pub in the first figures

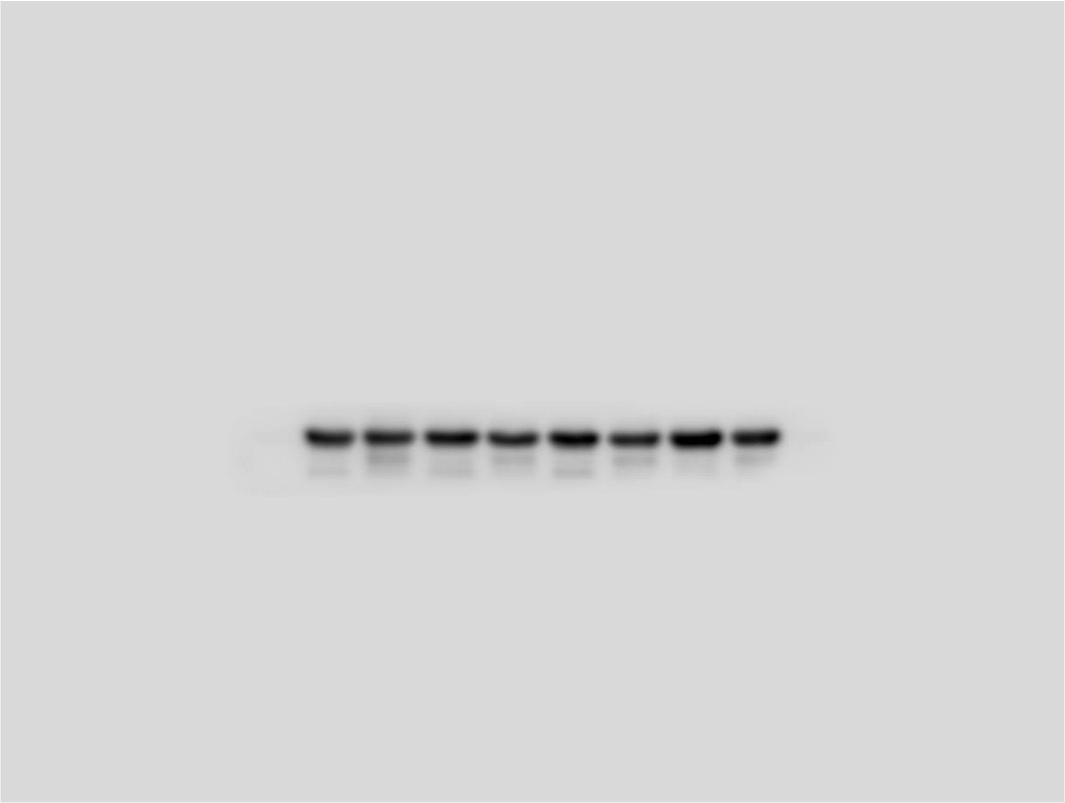

GAPDH_pub in the second figures

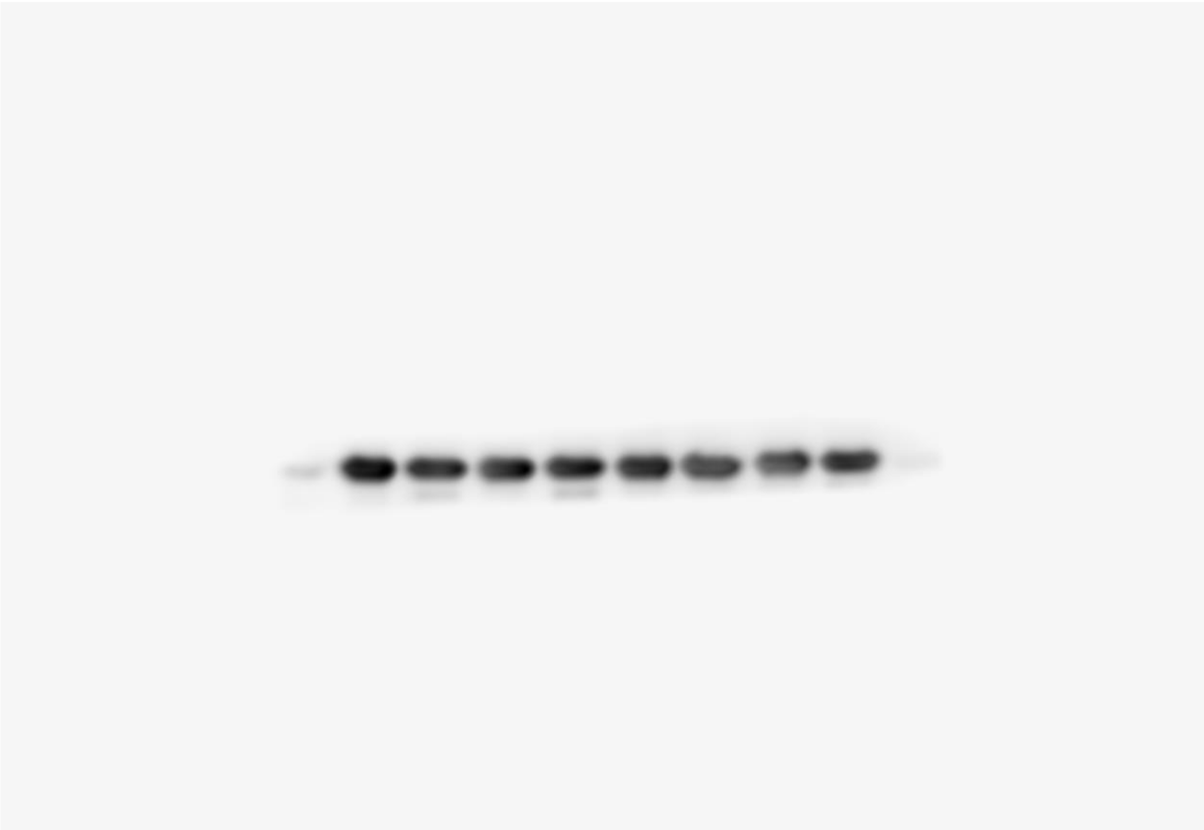

Figure 1F

A2789 SKOV3 OVCAR-3 IOSE-8 FAM172A_pub

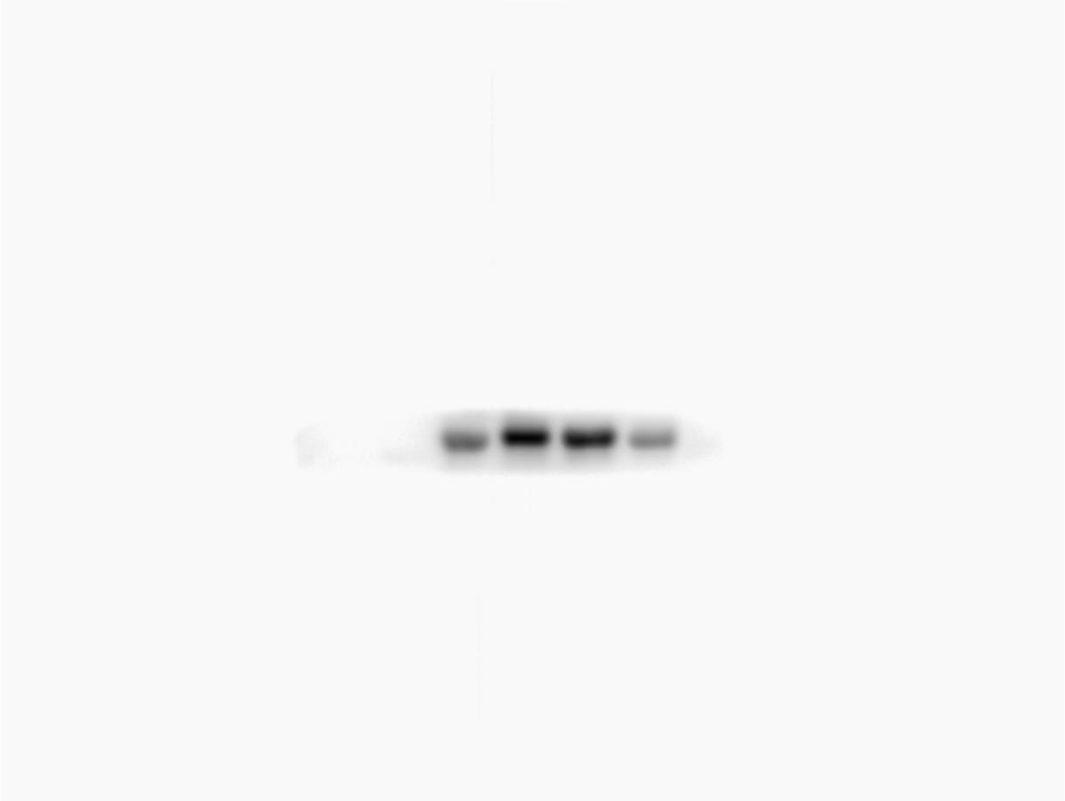

Gapdh_pub

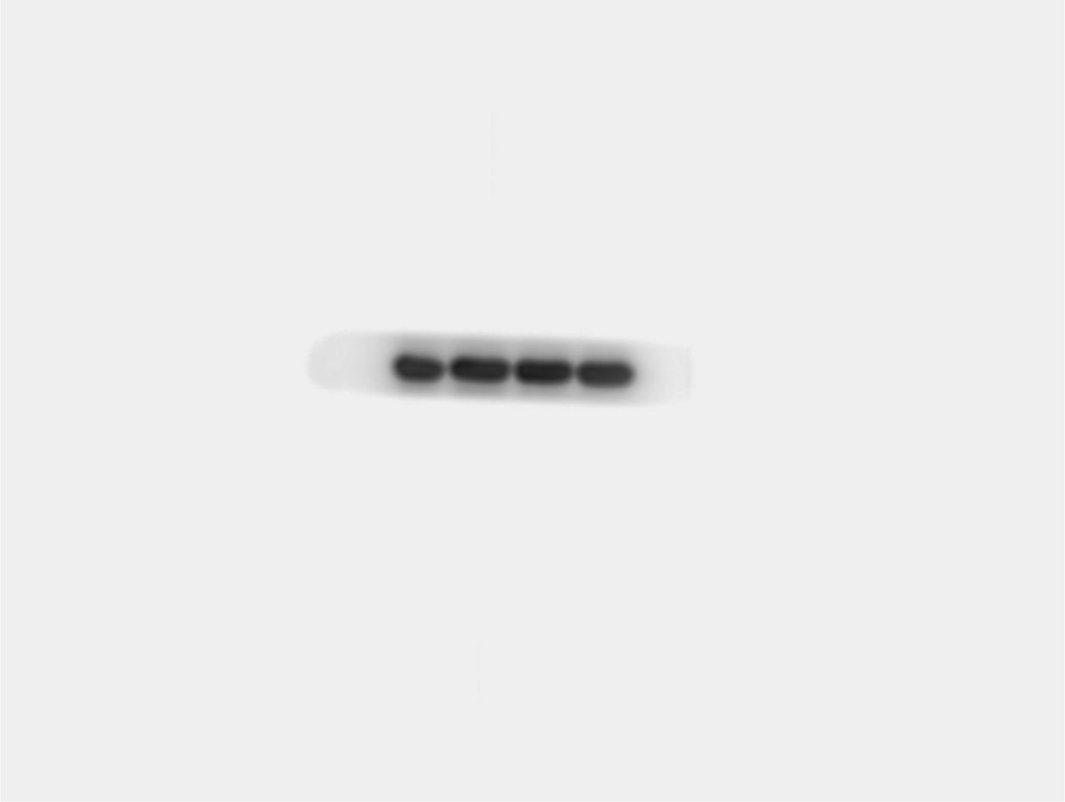

Figure 1H

A2780 FAM172A_pub

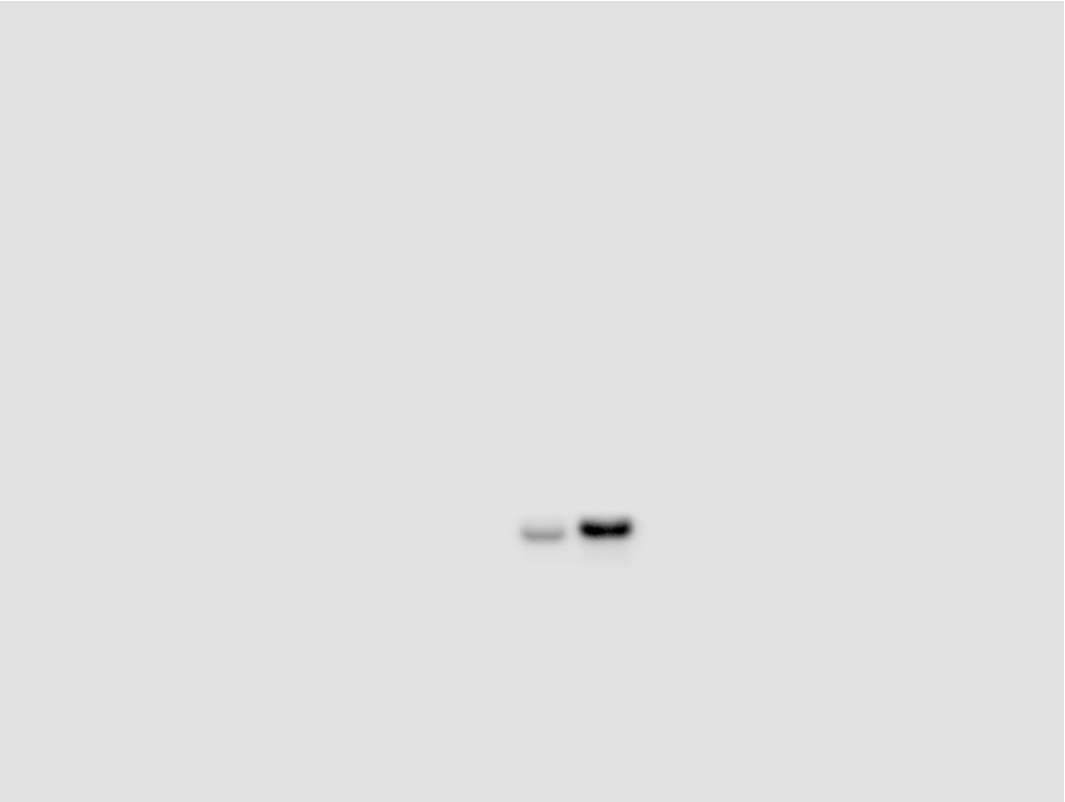

A2780 GAPDH_pub

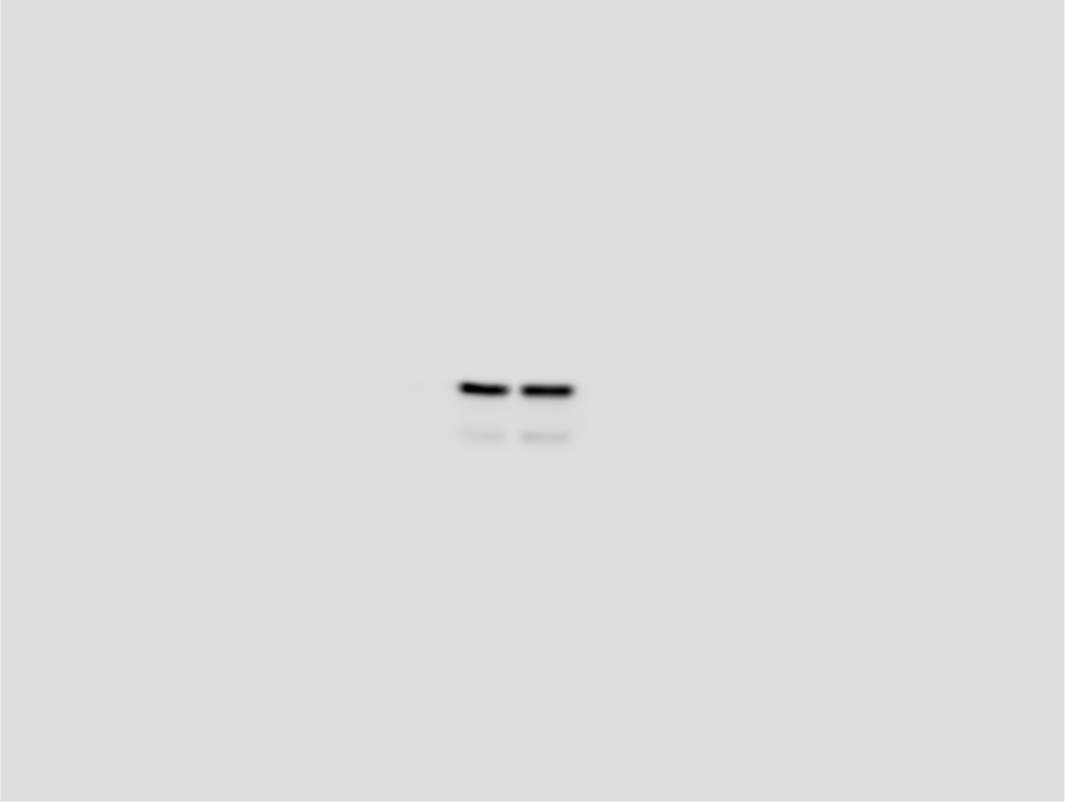

A2780 Ki67_pub

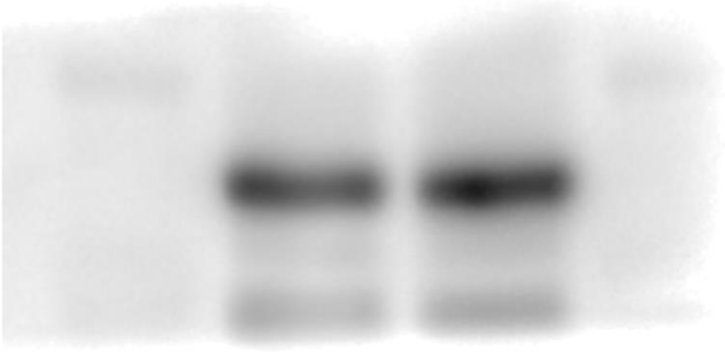

A2780 PCNA_pub

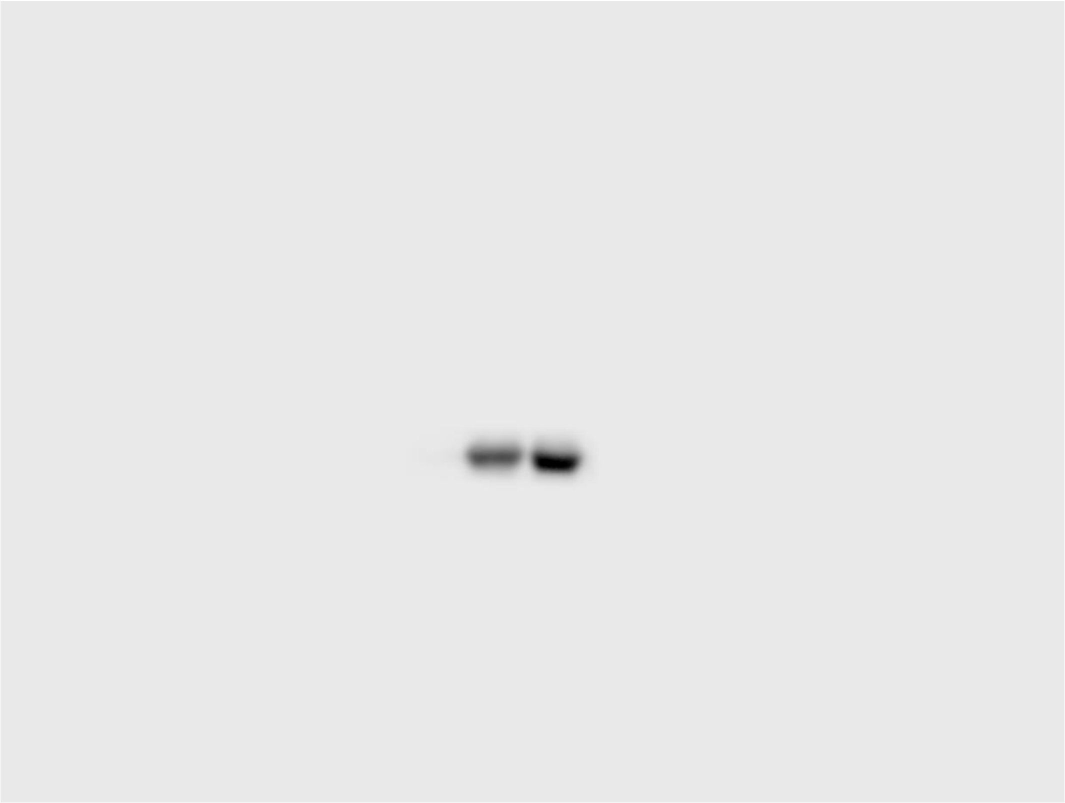

OVCAR-3 FAM172A_pub

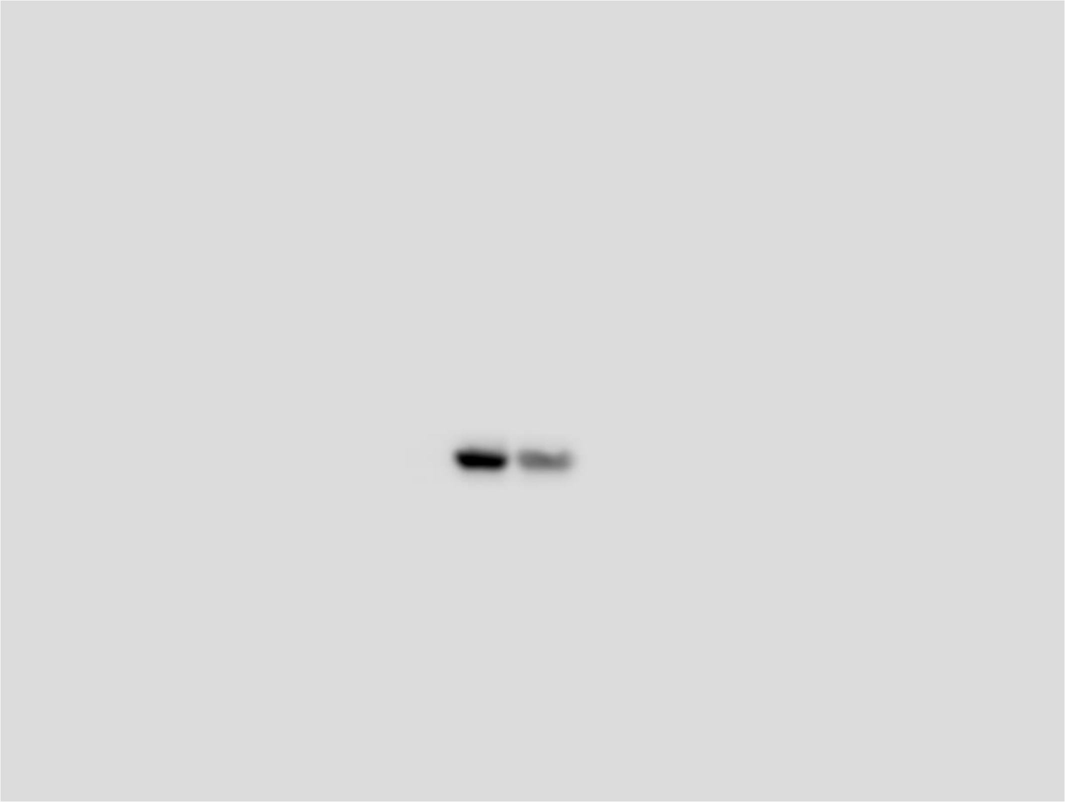

OVCAR-3 ki67_pub

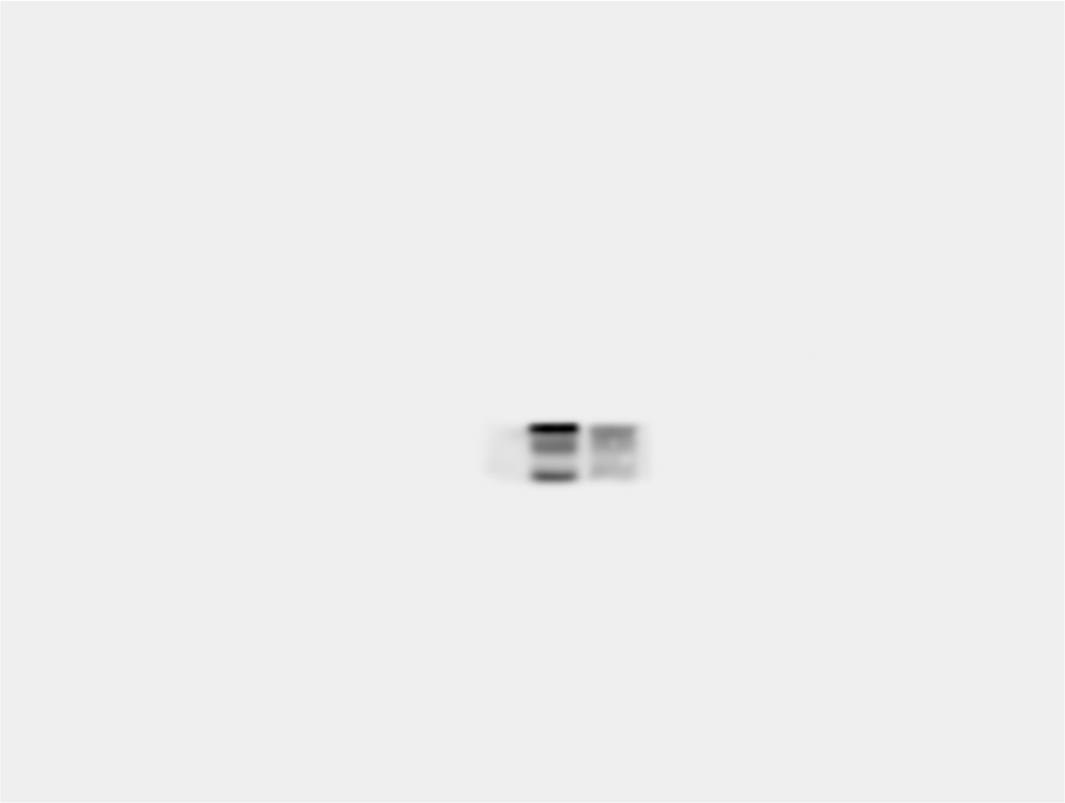

OVCAR-3 PCNA_pub

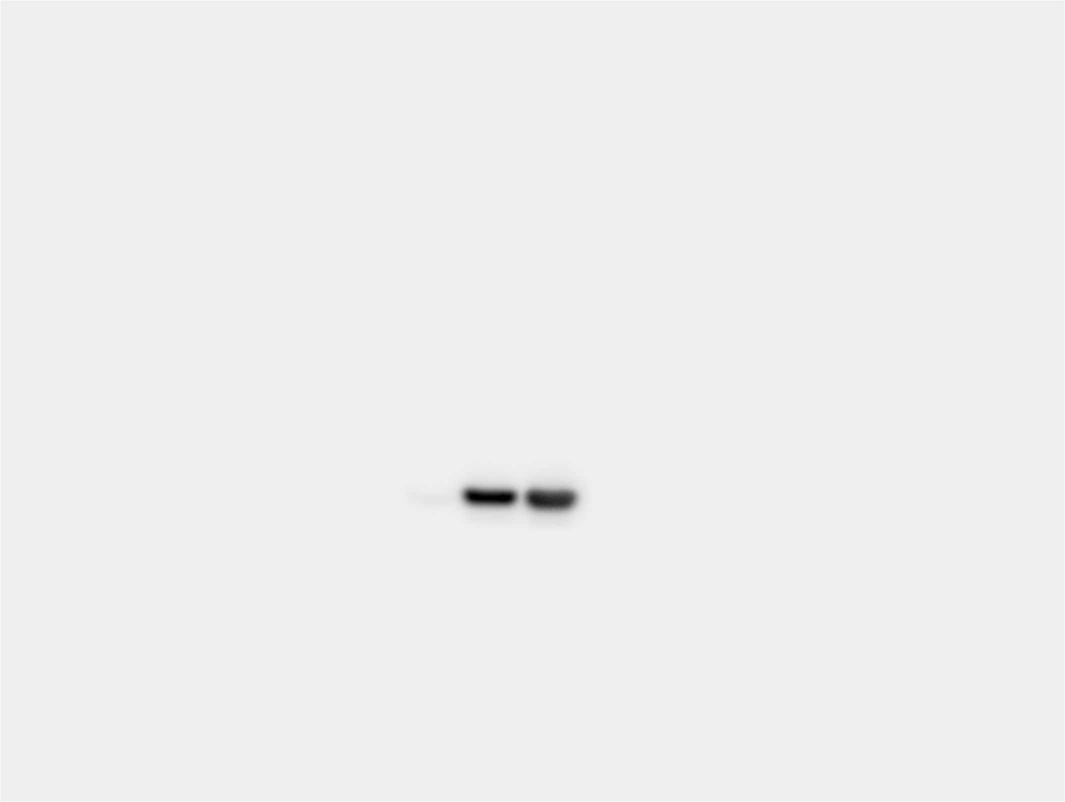

OVCAR-3gapdh_pub

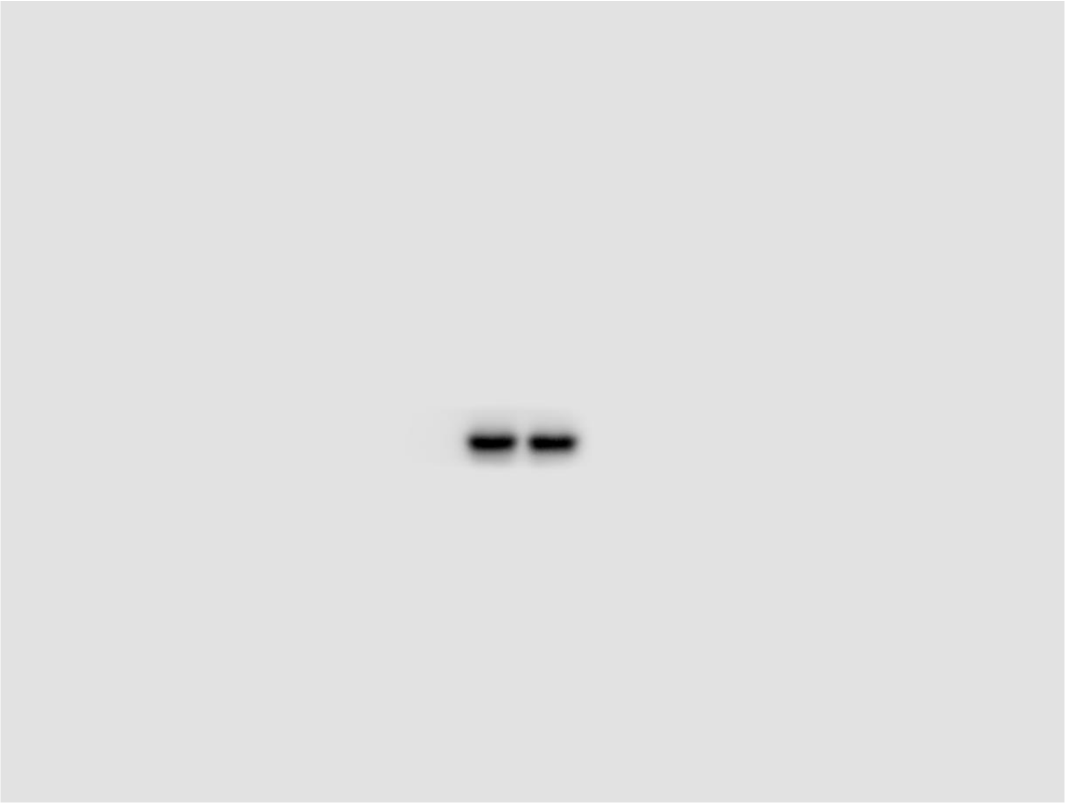

SKOV3 FAM172A_pub

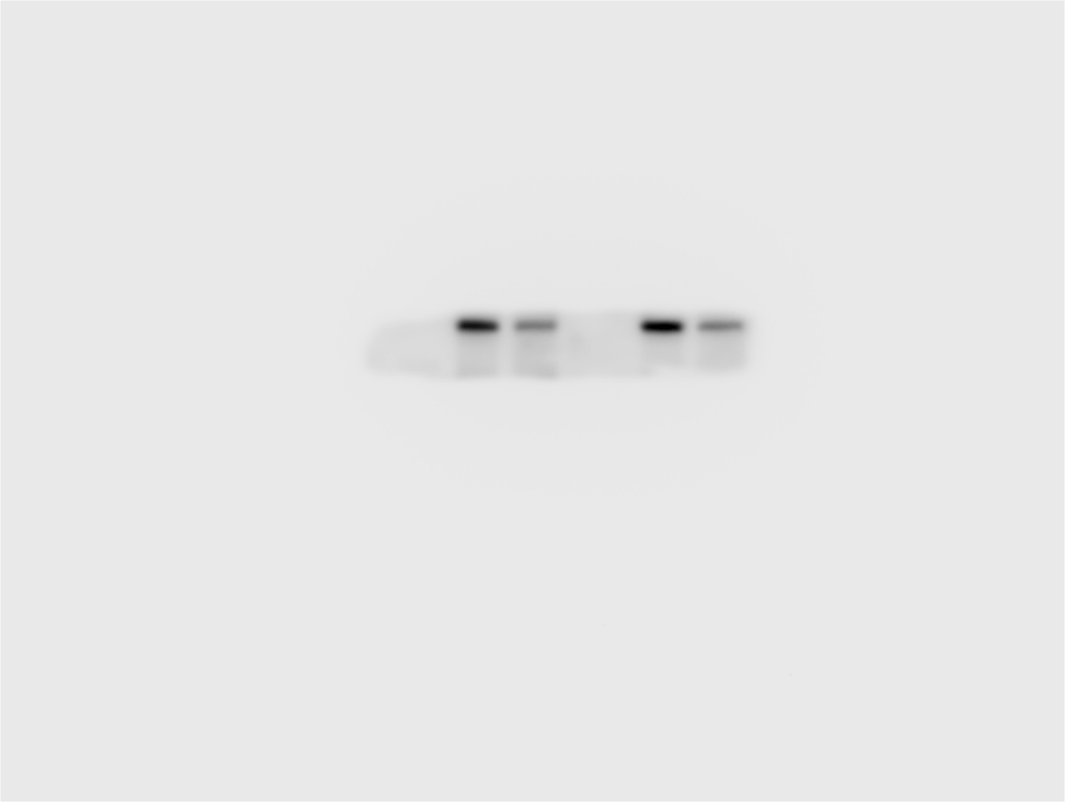

SKOV3 GAPDH_pub

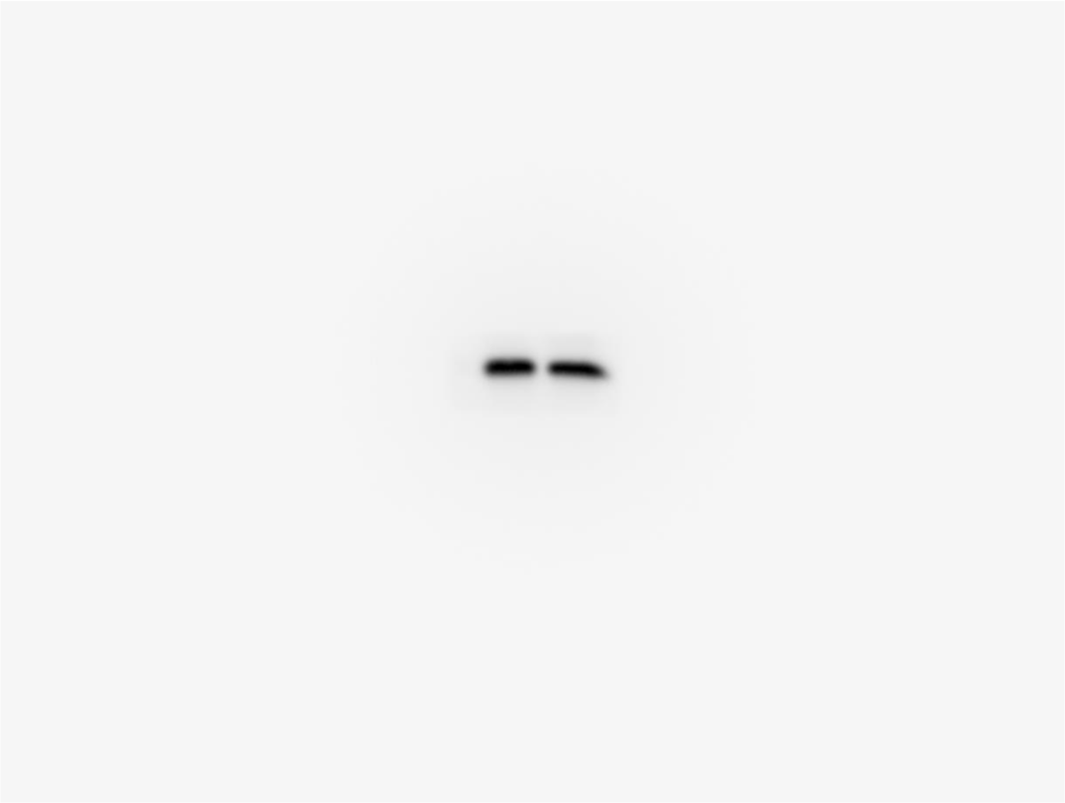

SKOV3 Ki67_pub

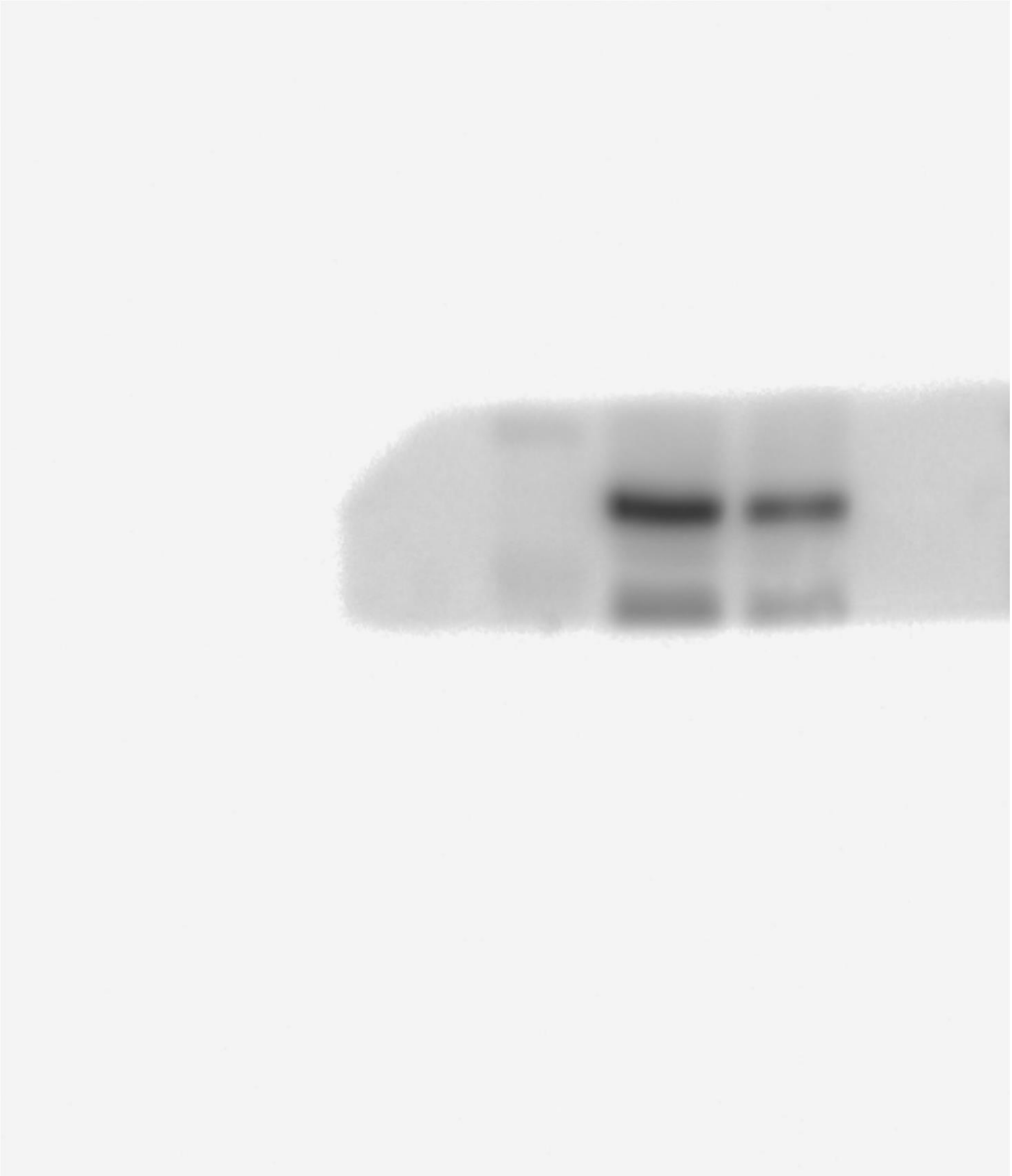

SKOV3 PCNA_pub

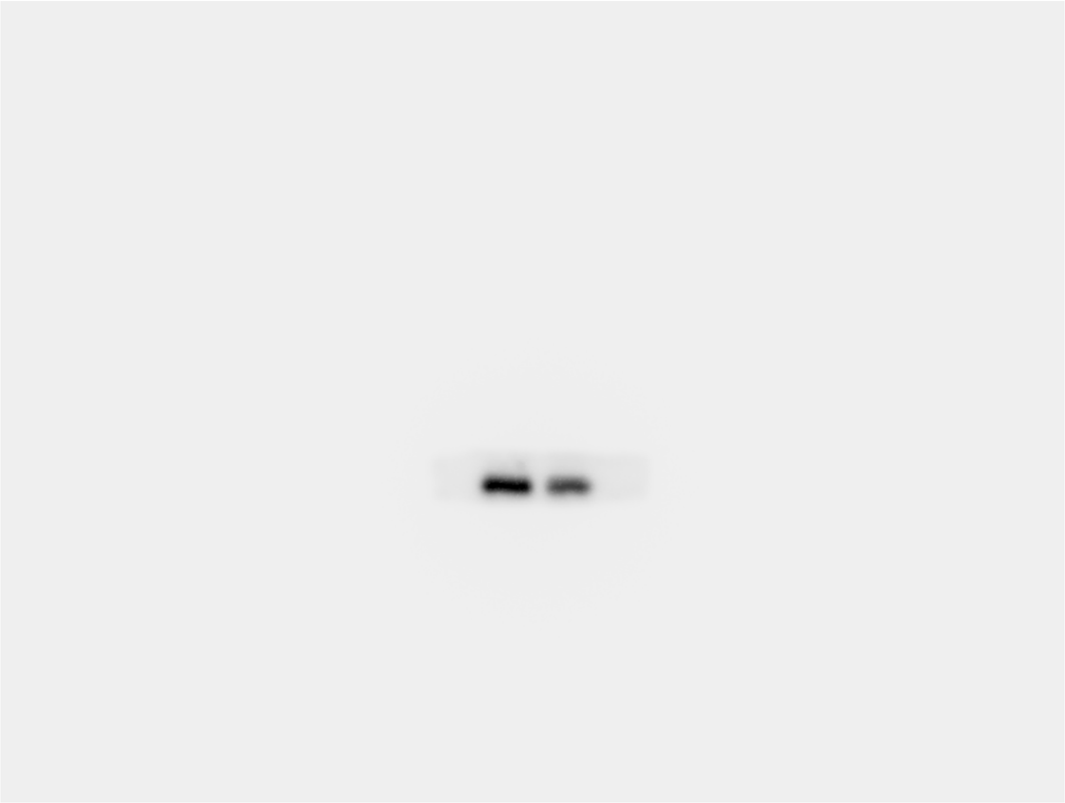

Figure 1L

A2780 N-cad_pub

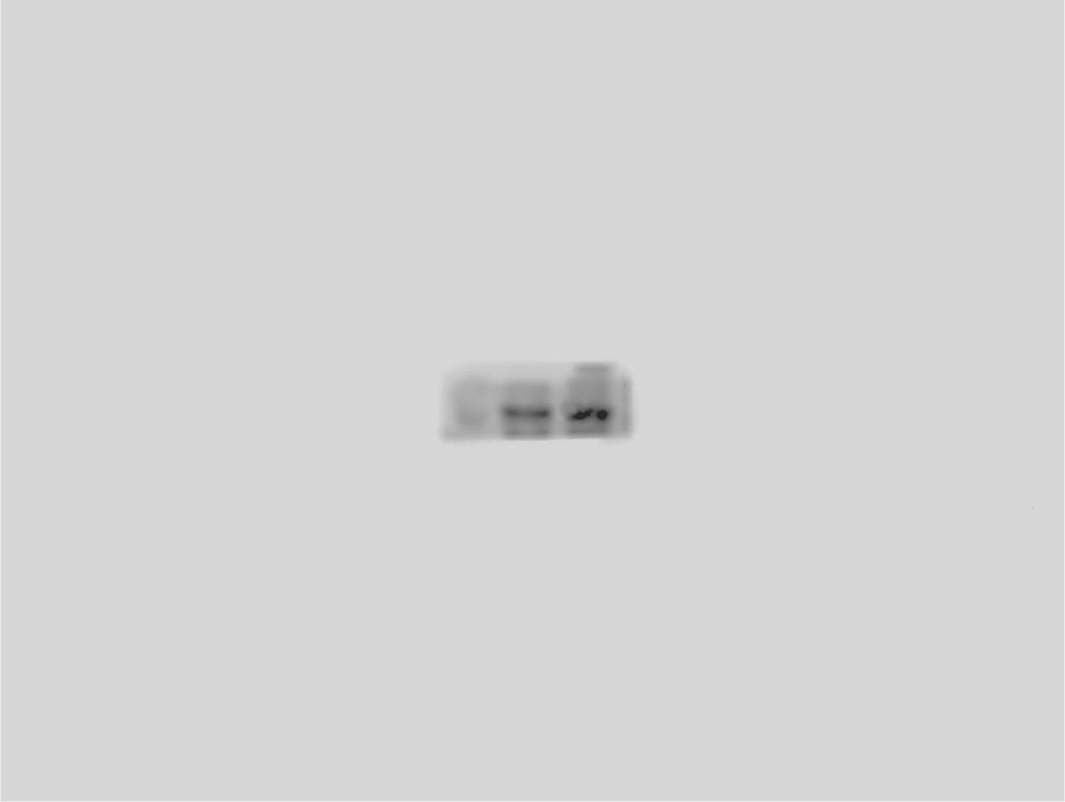

A2780 snail_pub

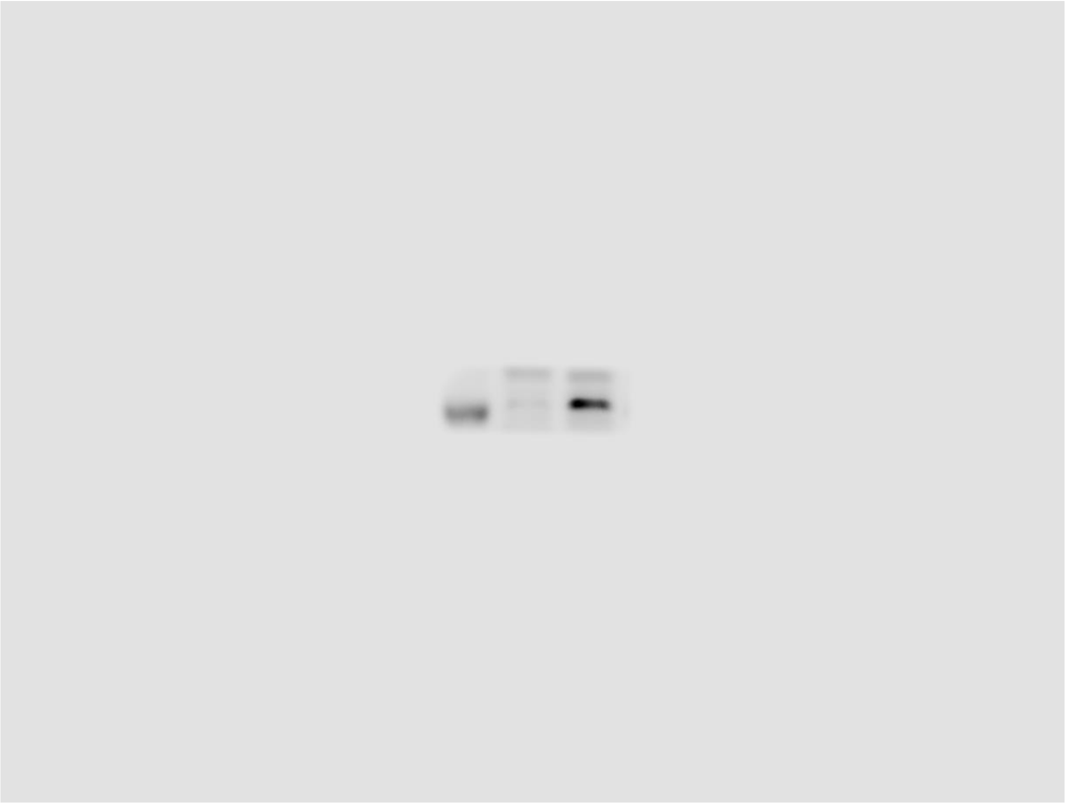

A2780fam172A_pub

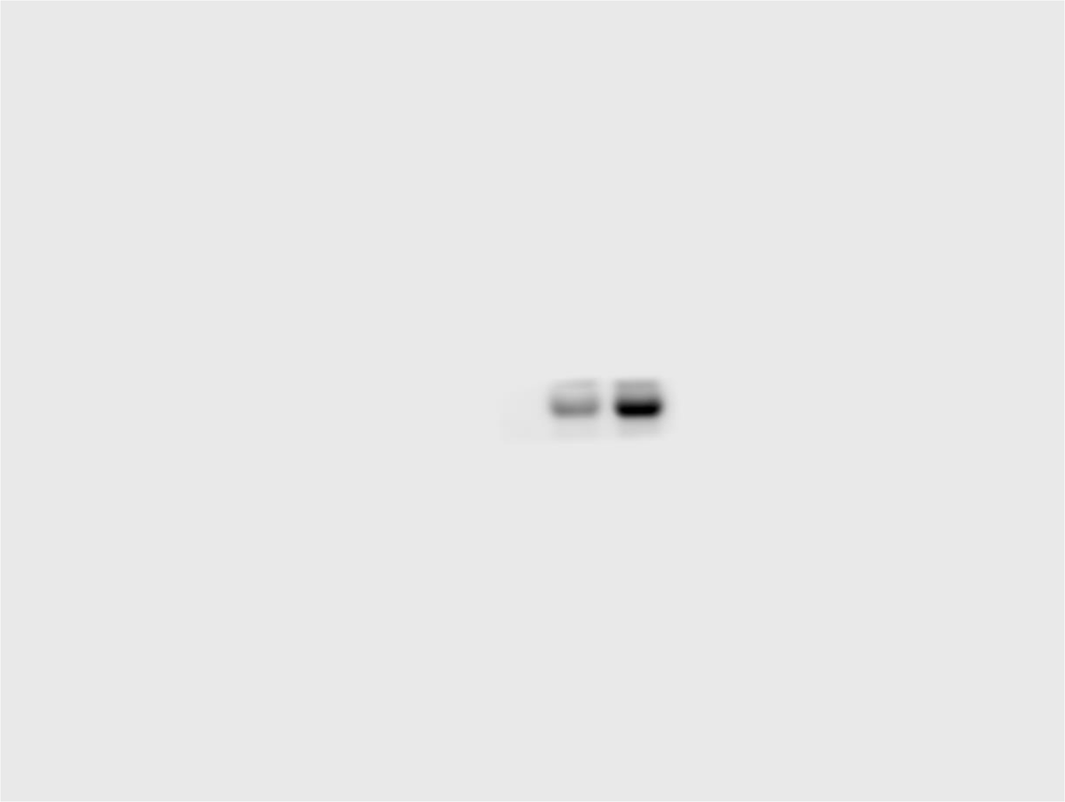

A2780GAPDH_pub

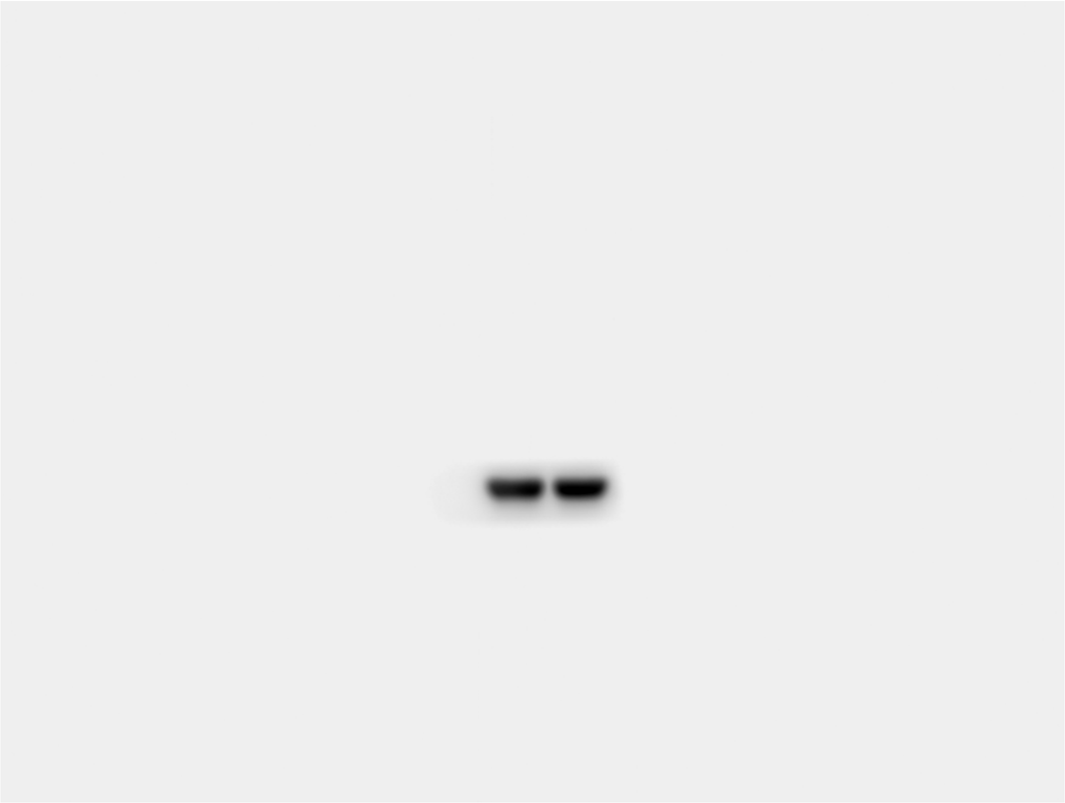

A2780slug_pub

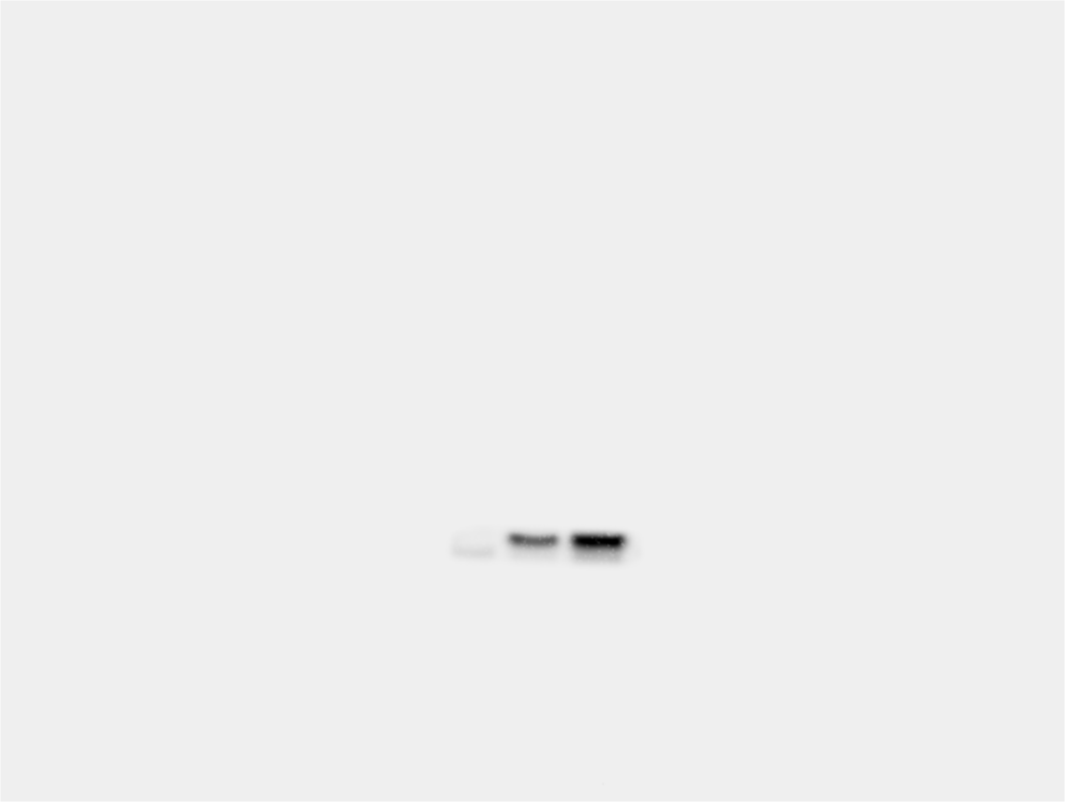

A2780twist_pub

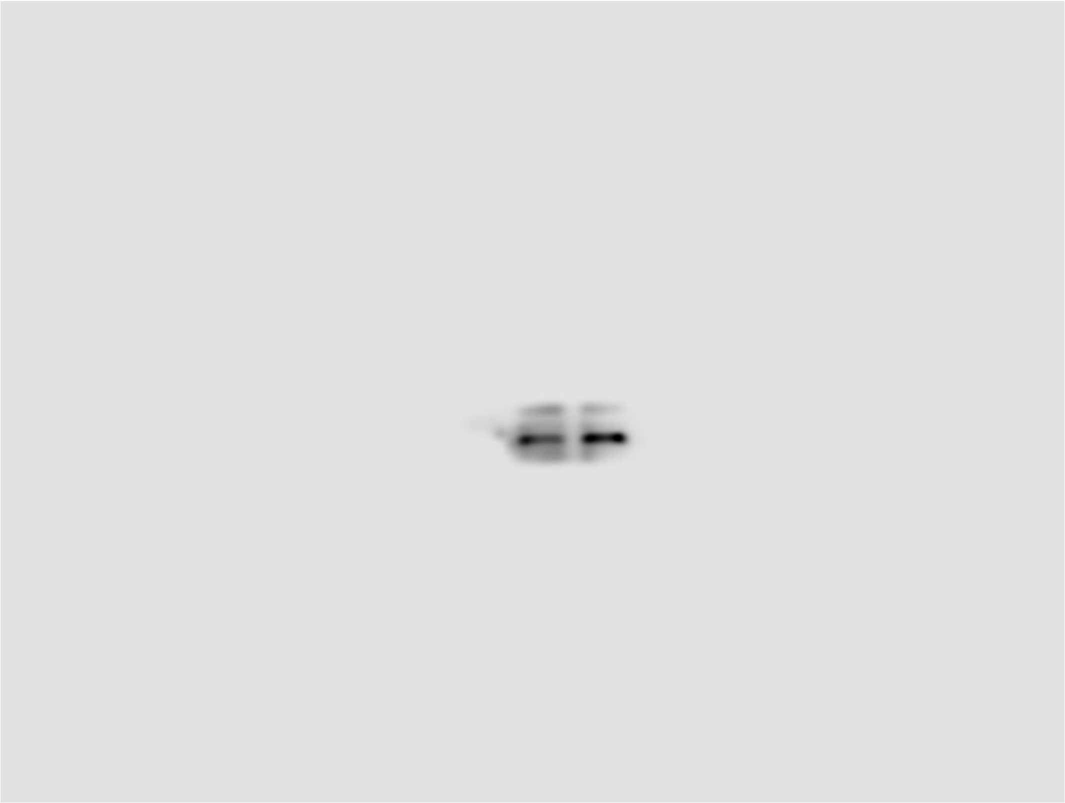

EcadA2780_pub

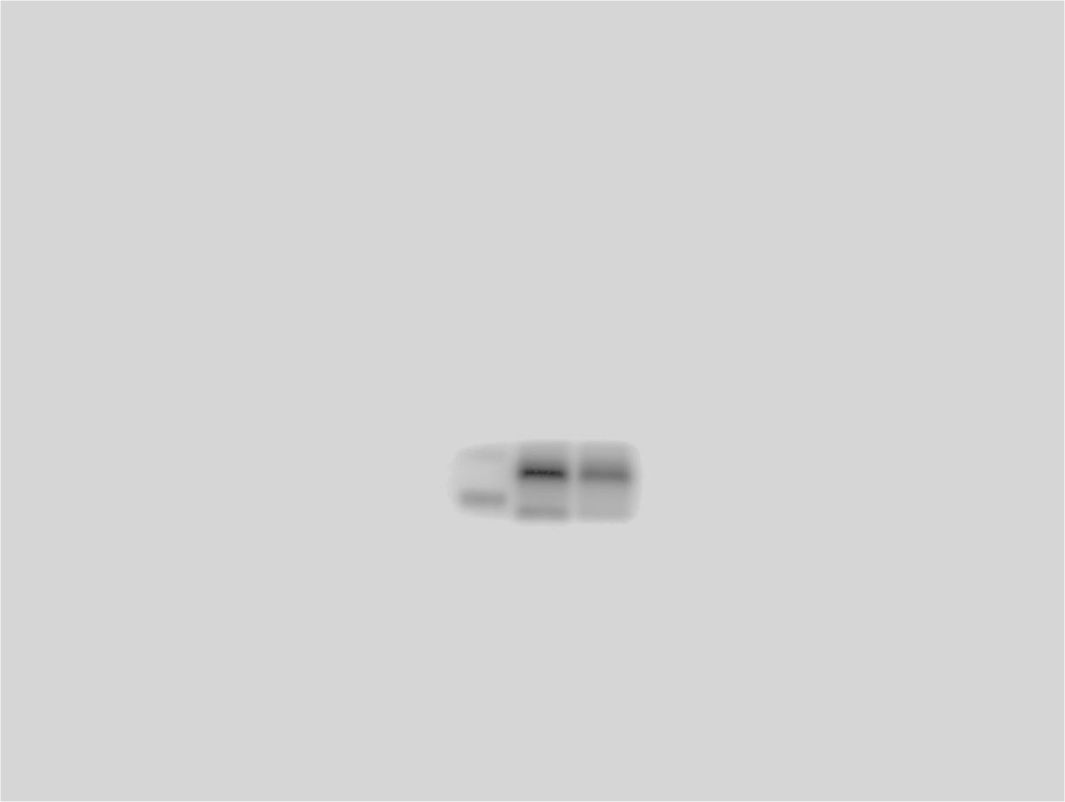

vimentinA2780_pub

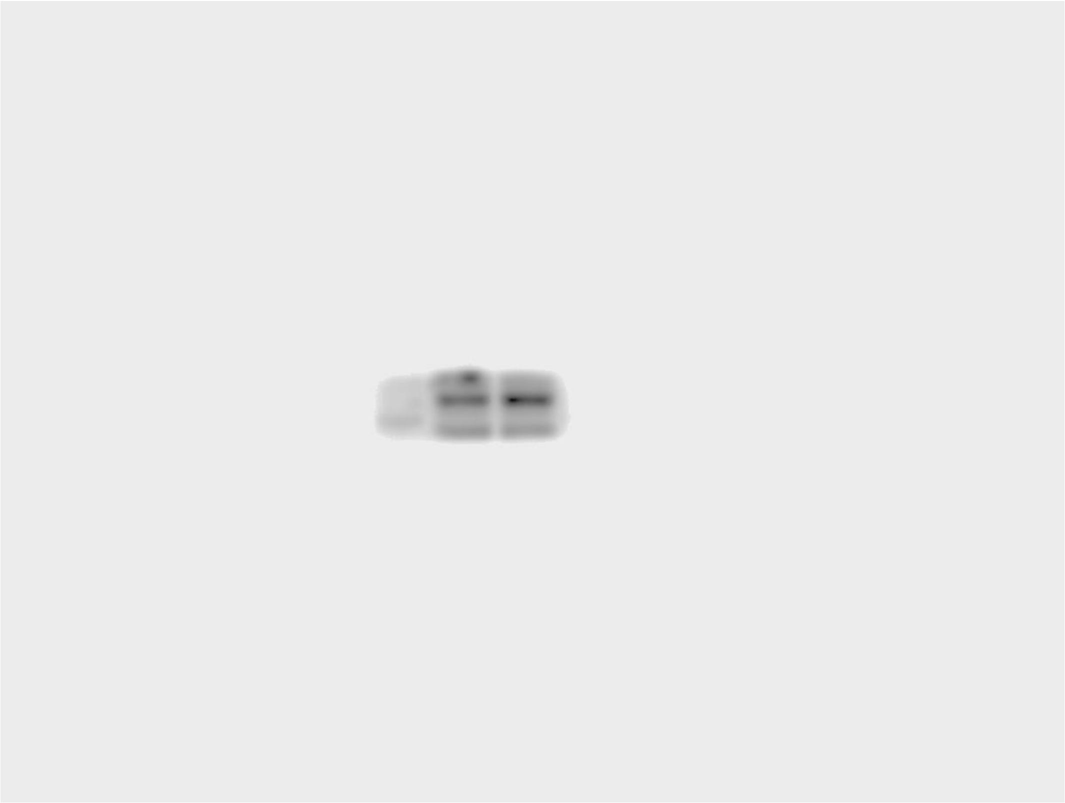

OV Sh Ecad_pub

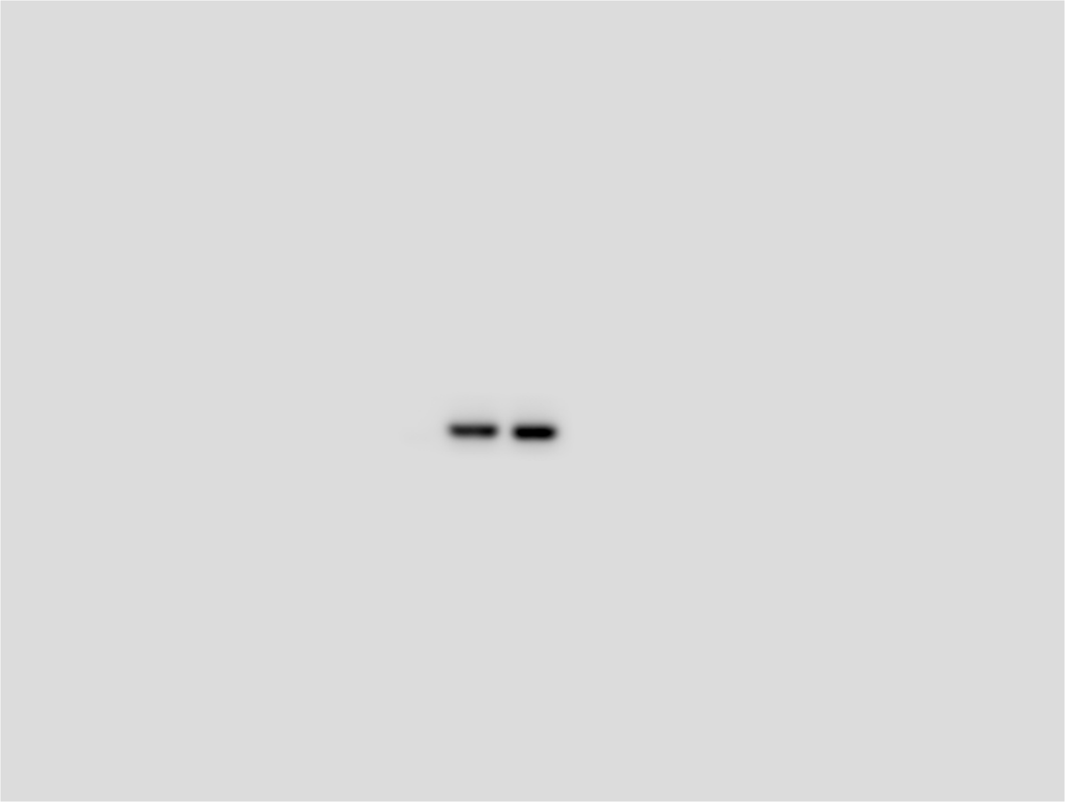

OV Sh fam_pub

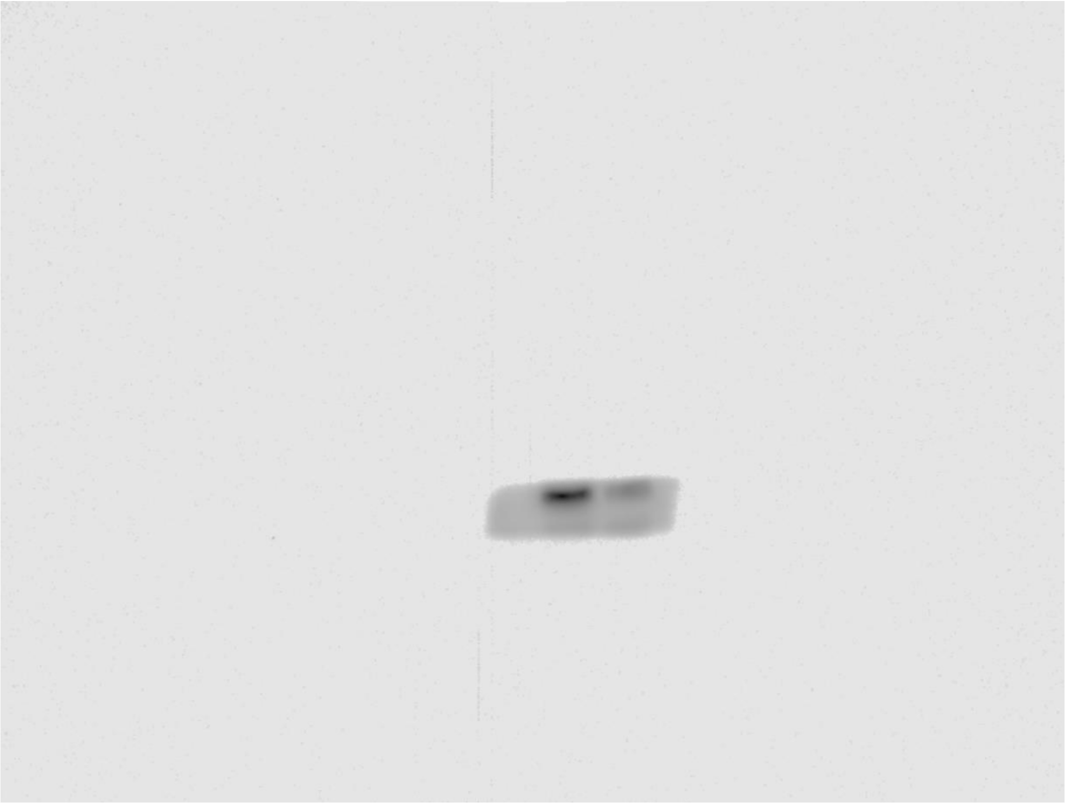

OV Sh gapdh_pub

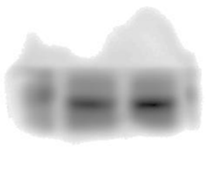

OV Sh N-cad_pub

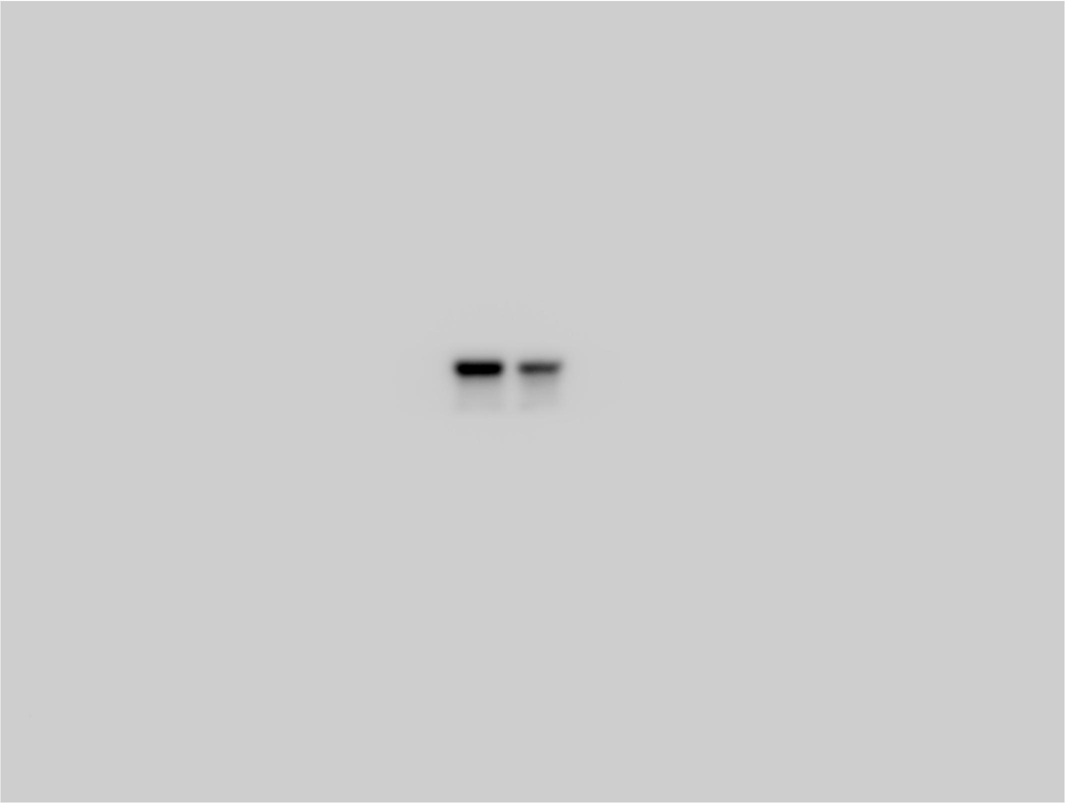

OV sh Slug_pub

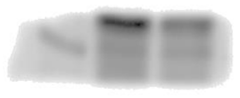

OV Sh snail_pub

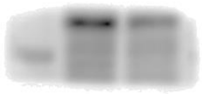

OV sh twist_pub

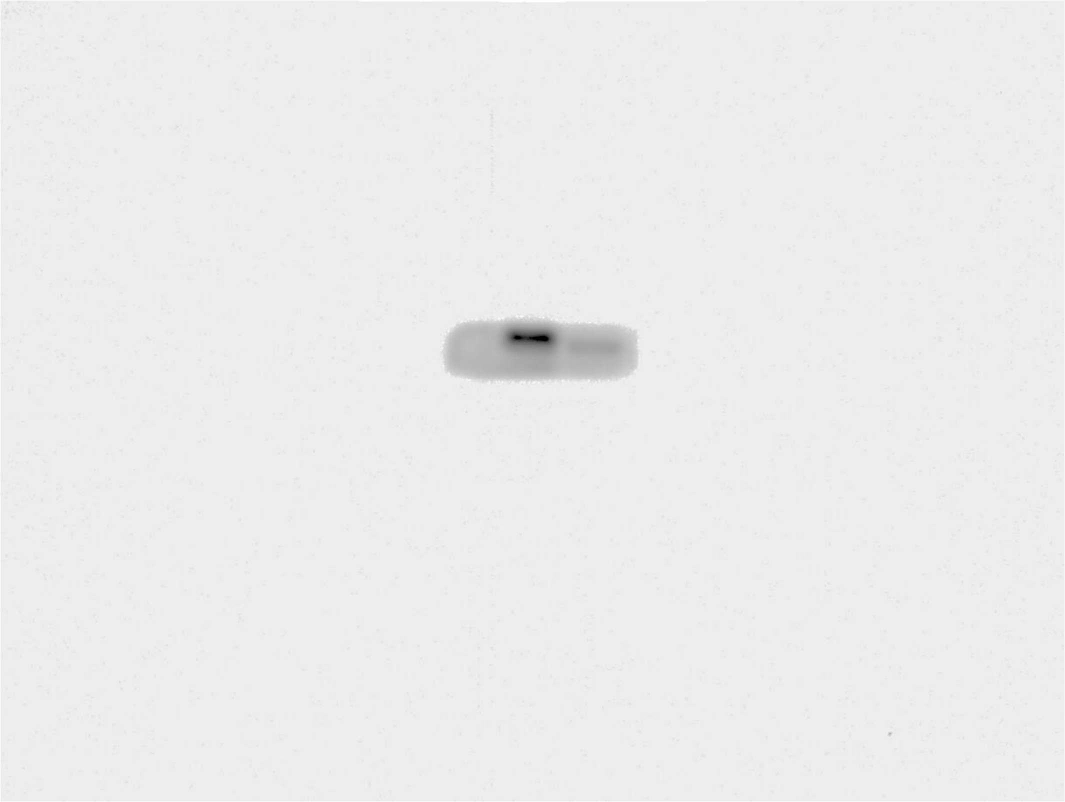

OV Sh vimentin_pub

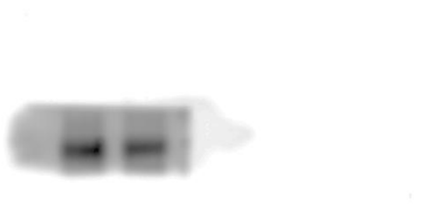

SKO Ecad_pub

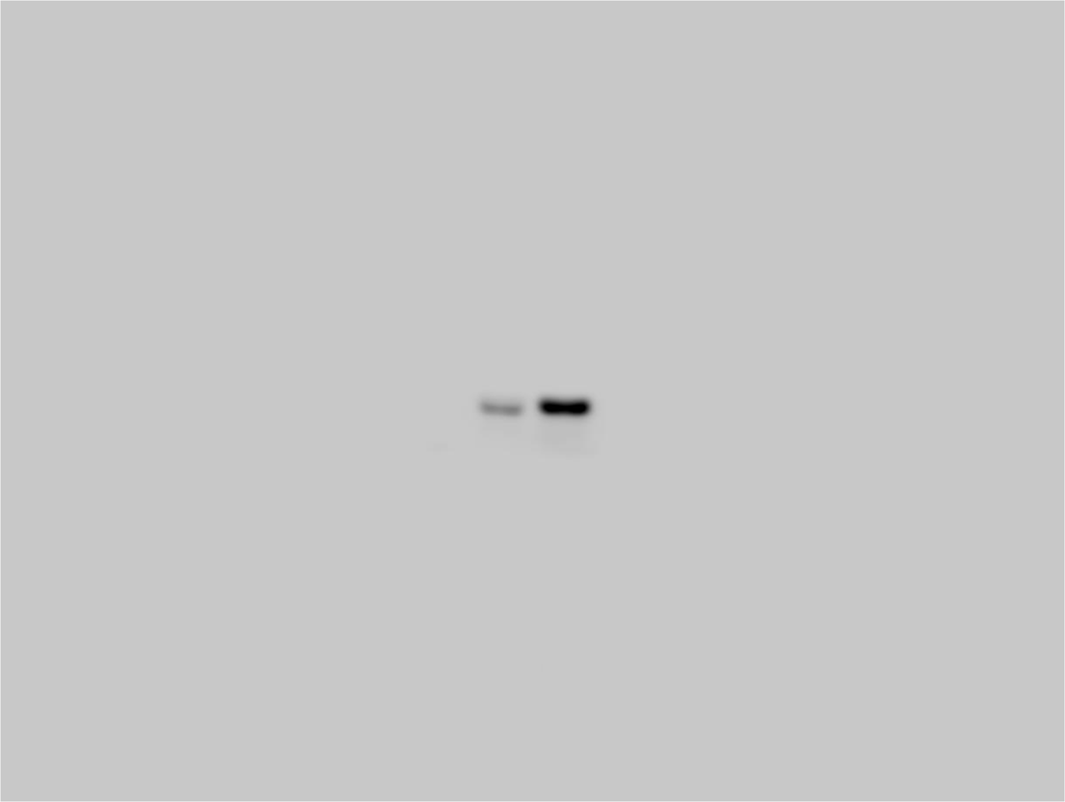

SKO fam172A_pub

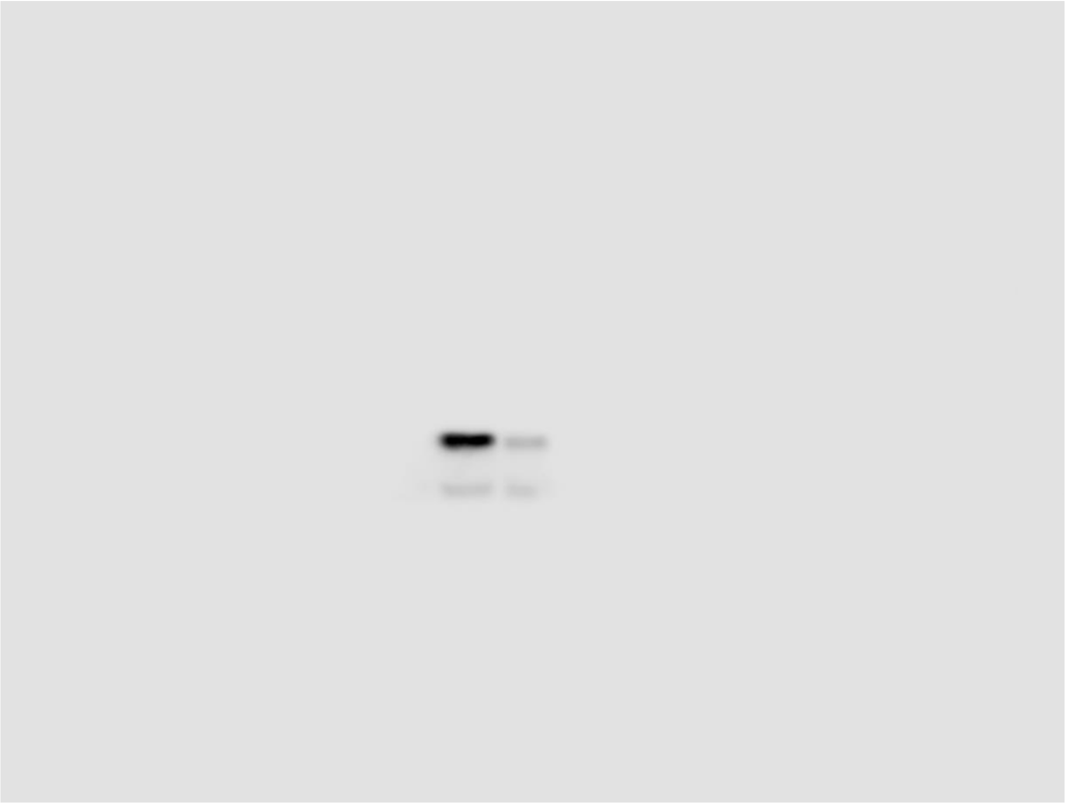

SKO gapdh_pub

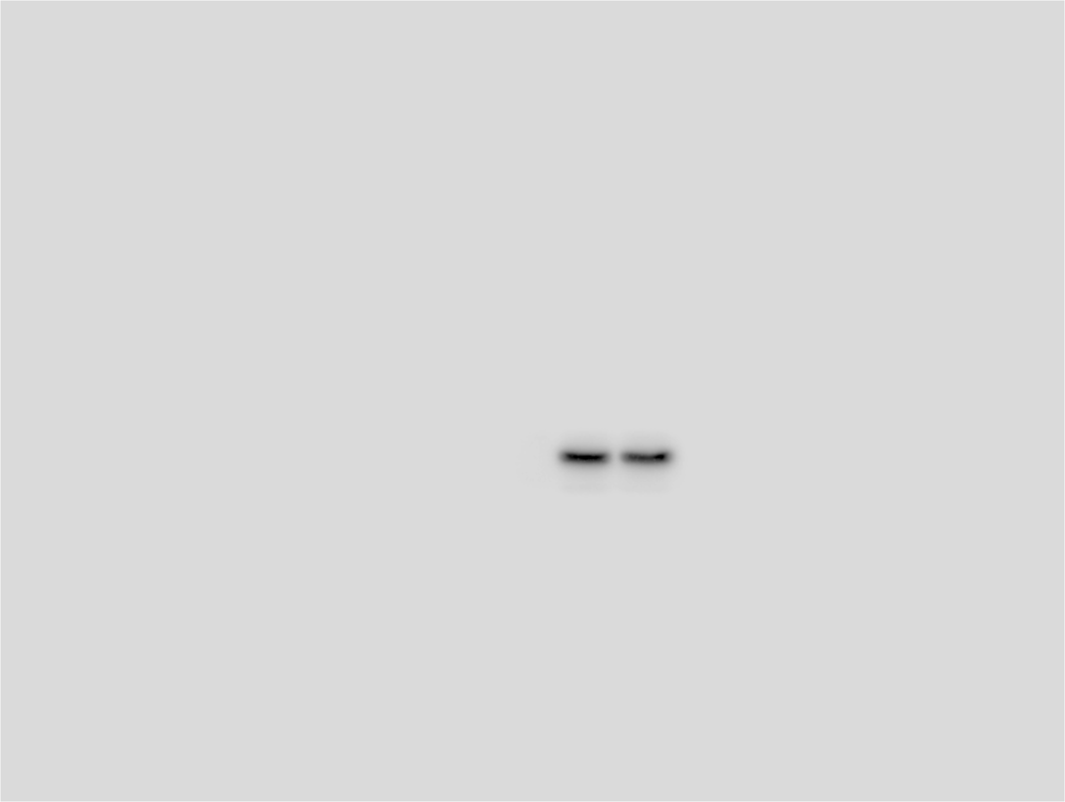

SKO Ncad_pub

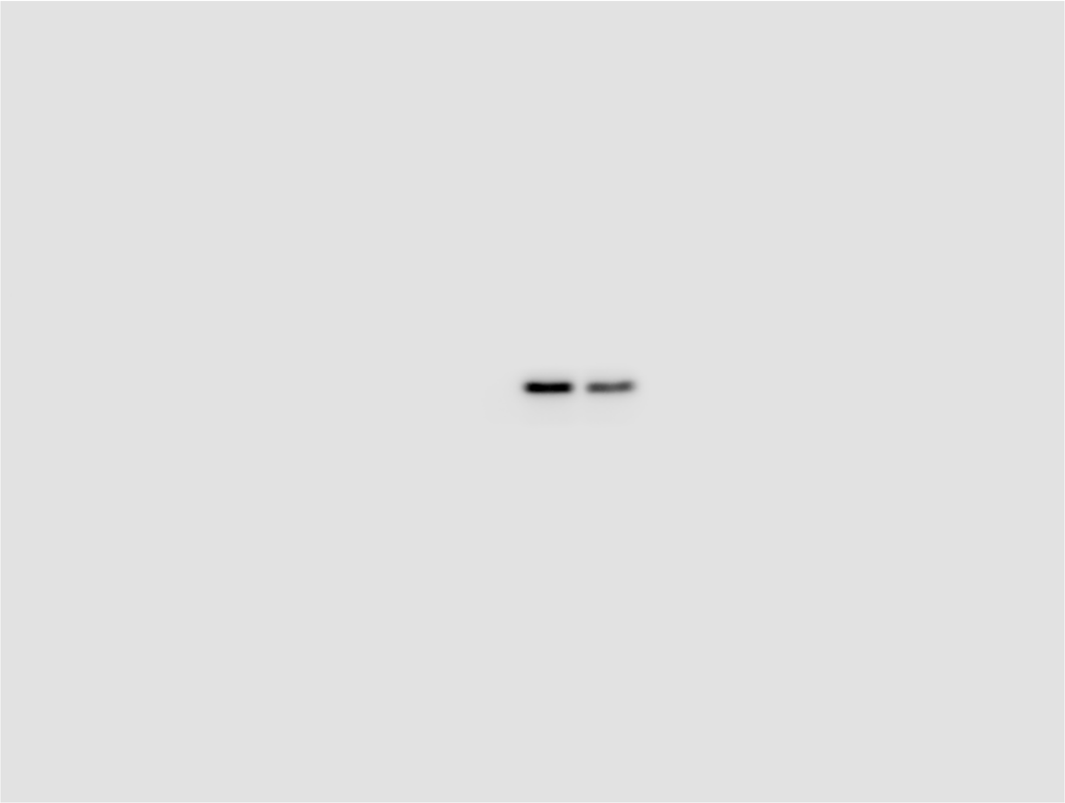

SKO sh slug_pub

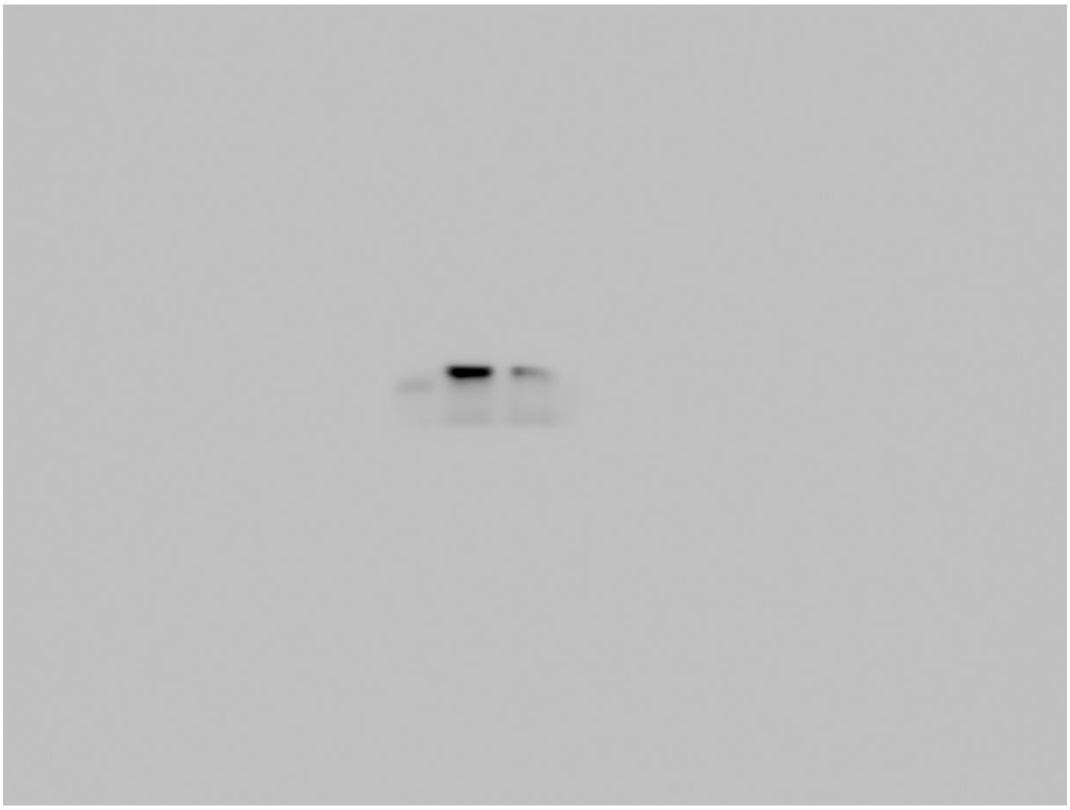

SKO Sh snail_pub

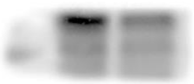

SKO twist_pub

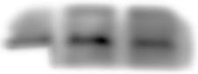

SKO vimentin_pub

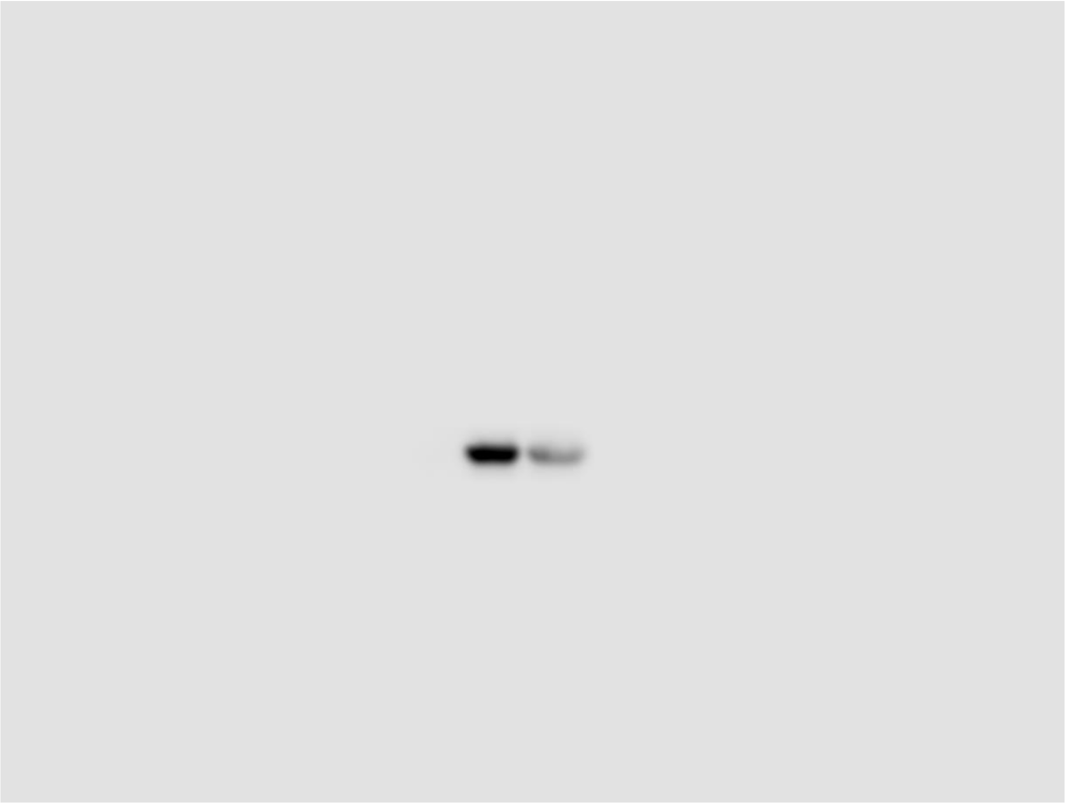

Figure 2B
A2780 bax_pub

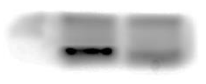

A2780 bcl-2_pub

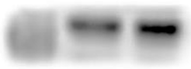

A2780 cleavedcas3_pub

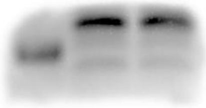

A2780 cleavedcas9_pub

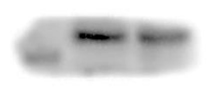

A2780 gapdh_pub

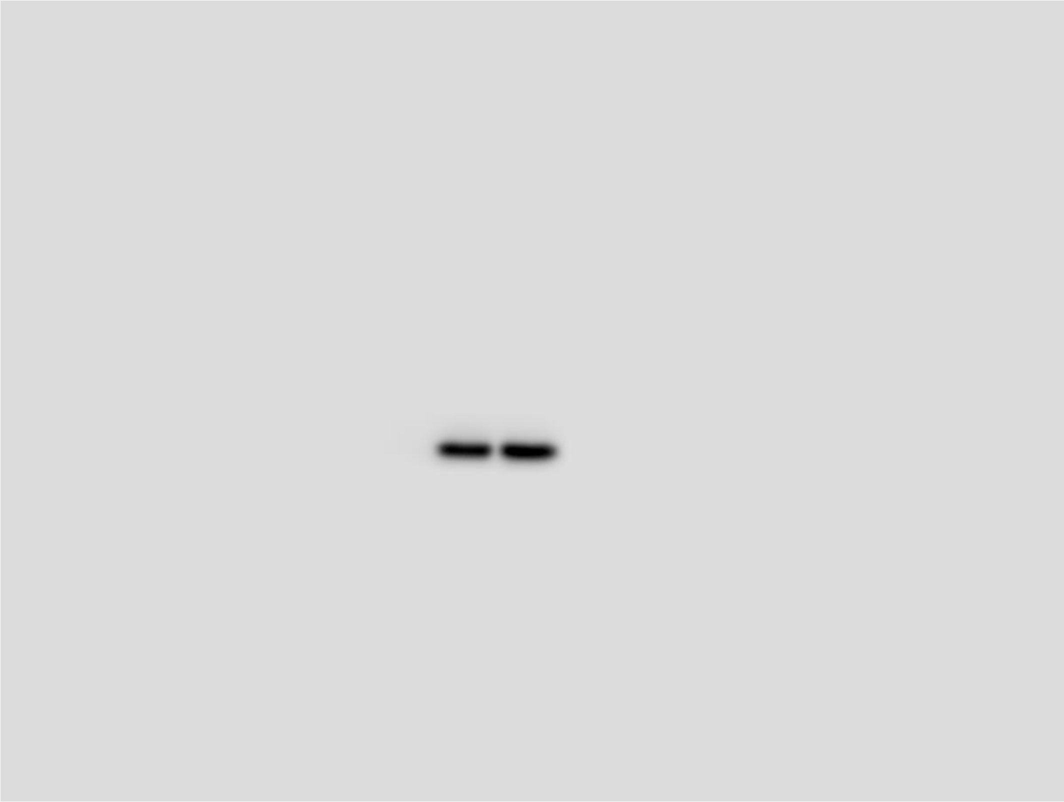

famA2780_pub

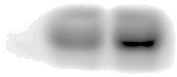

2OVCAR-3　fam_pub

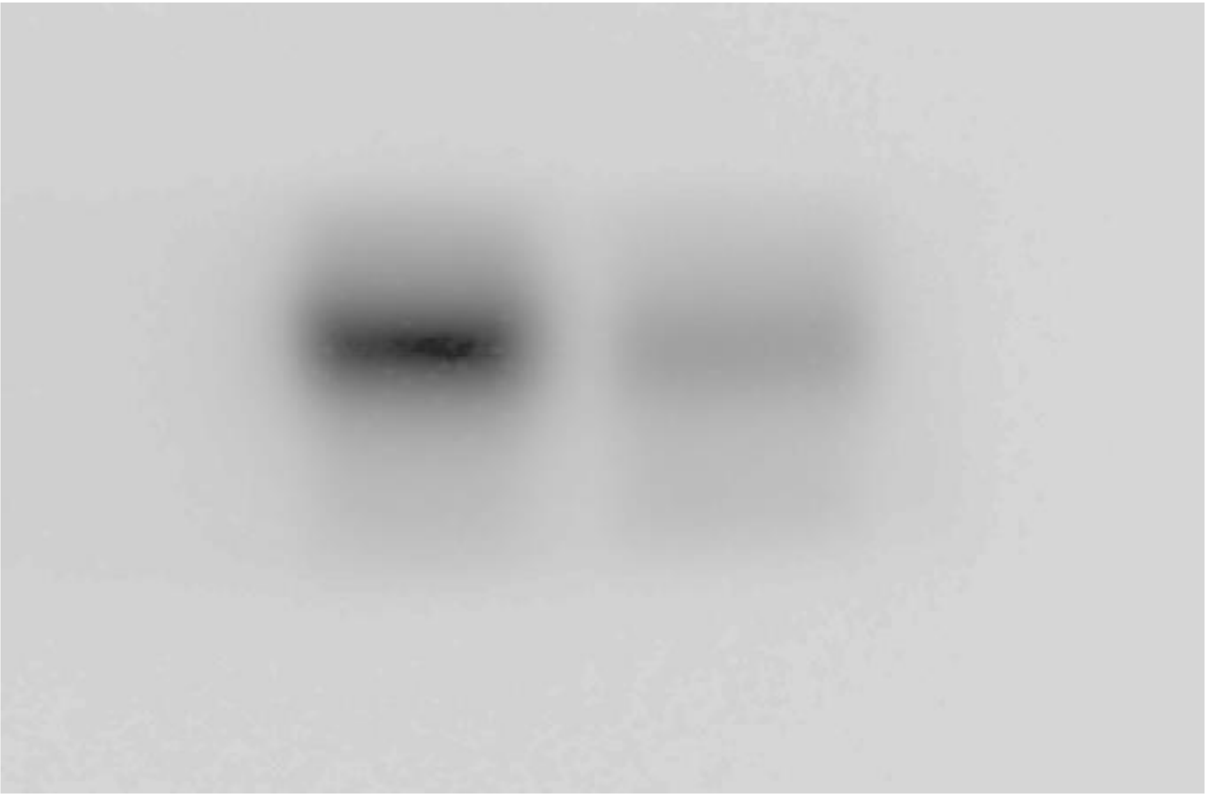

OV leavedcas3_pub

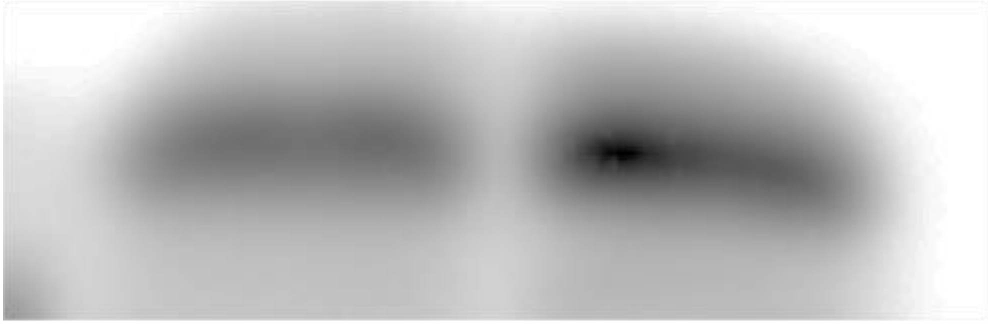

OVCAR-3 BCL_pub

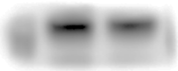

OVCAR-3 bax_pub

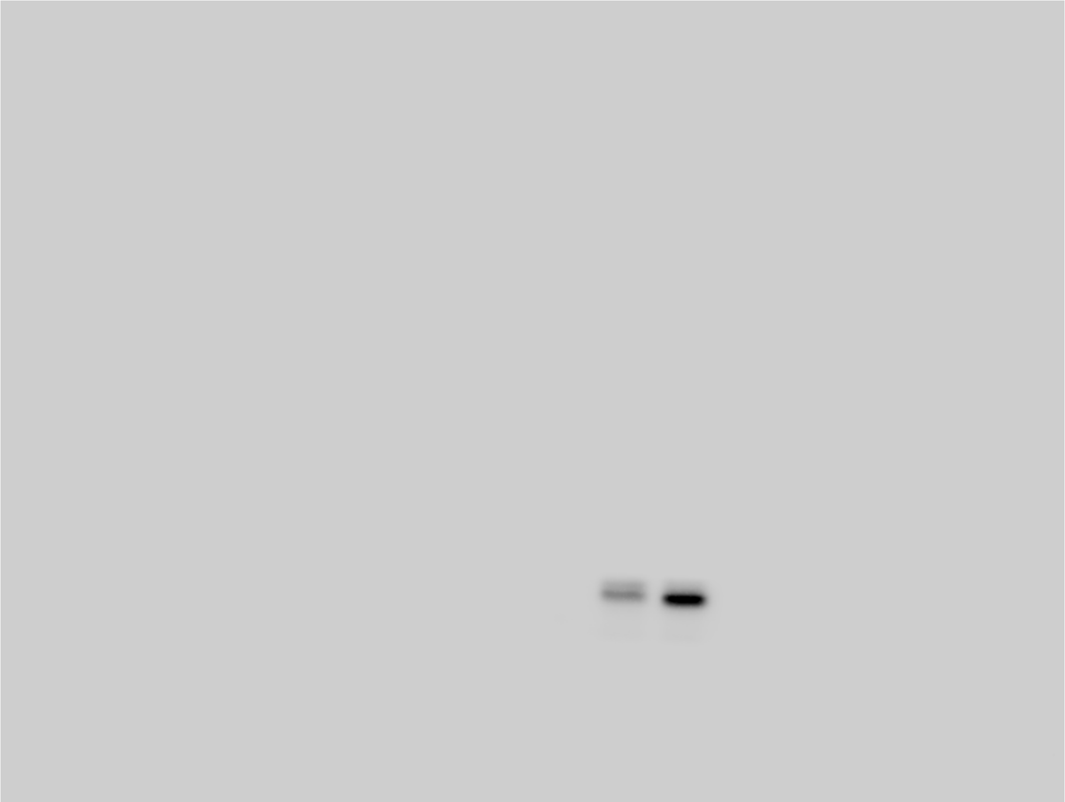

OVCAR-3 cleavedcas9_pub

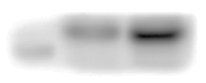

OVCAR-3 GAPDH_pub

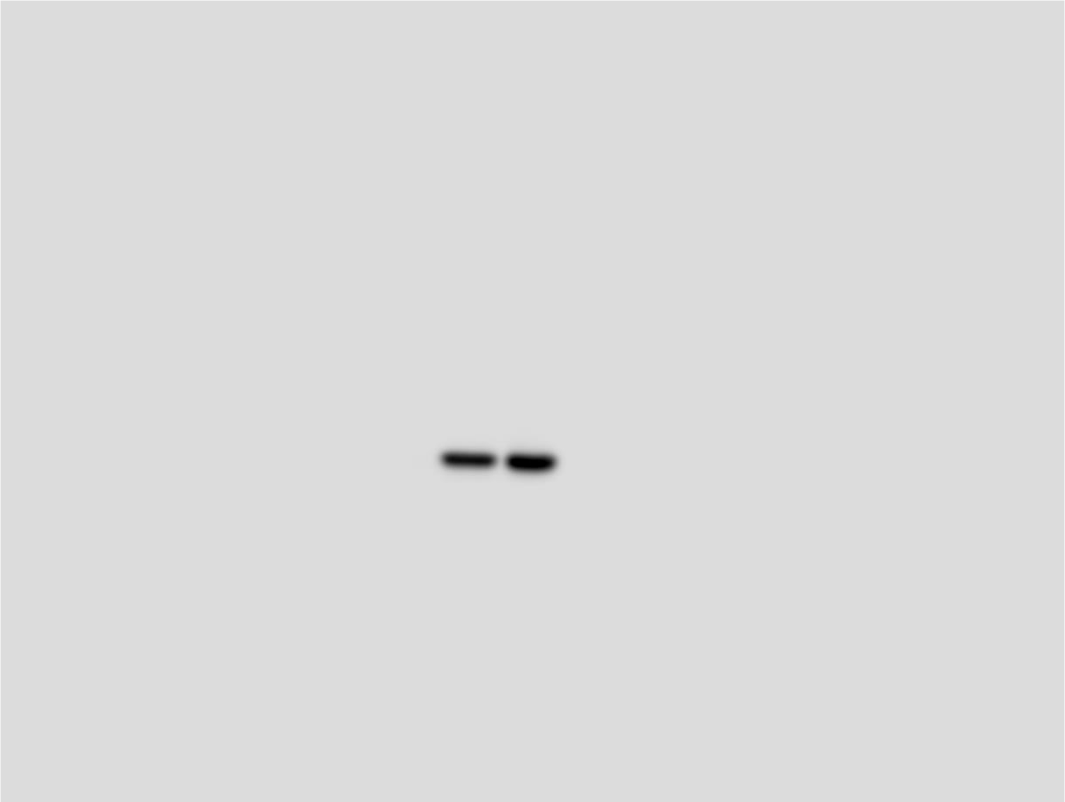

SKO FAM172A_pub

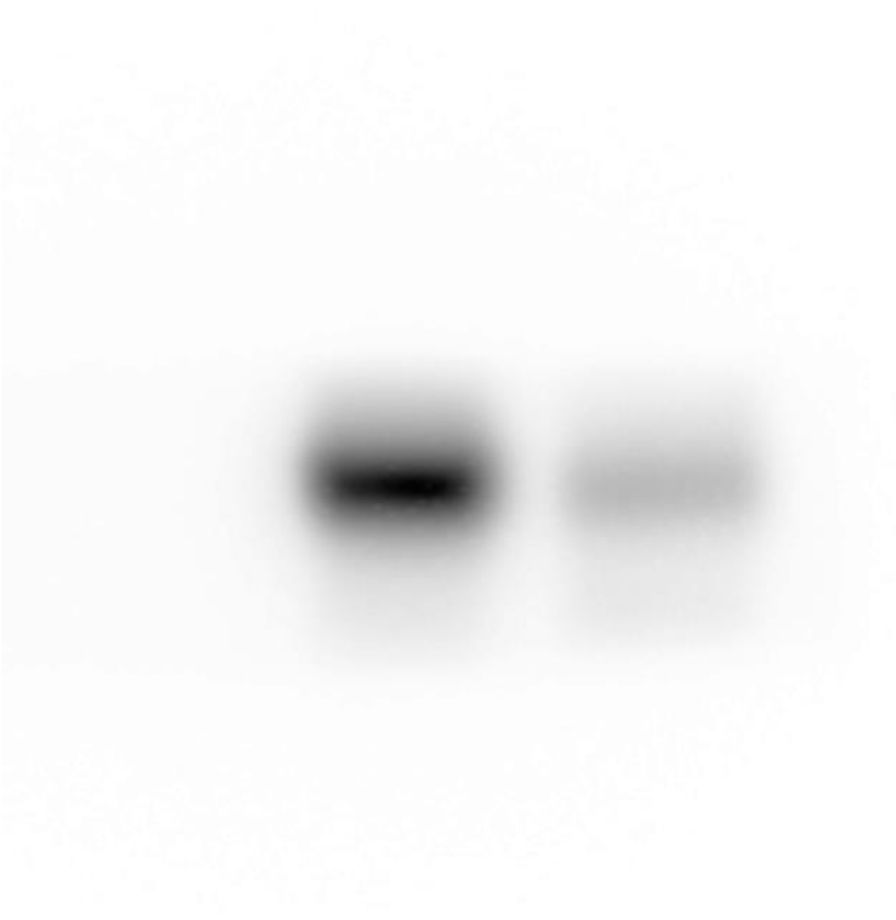

SKOV3 bax_pub

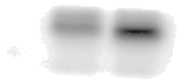

SKOV3 BCL_pub

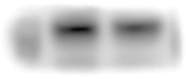

SKOV3 cleavedcas3_pub

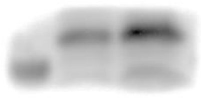

SKOV3 cleavedcas9_pub

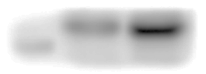

SKOV3 GAPDH_pub

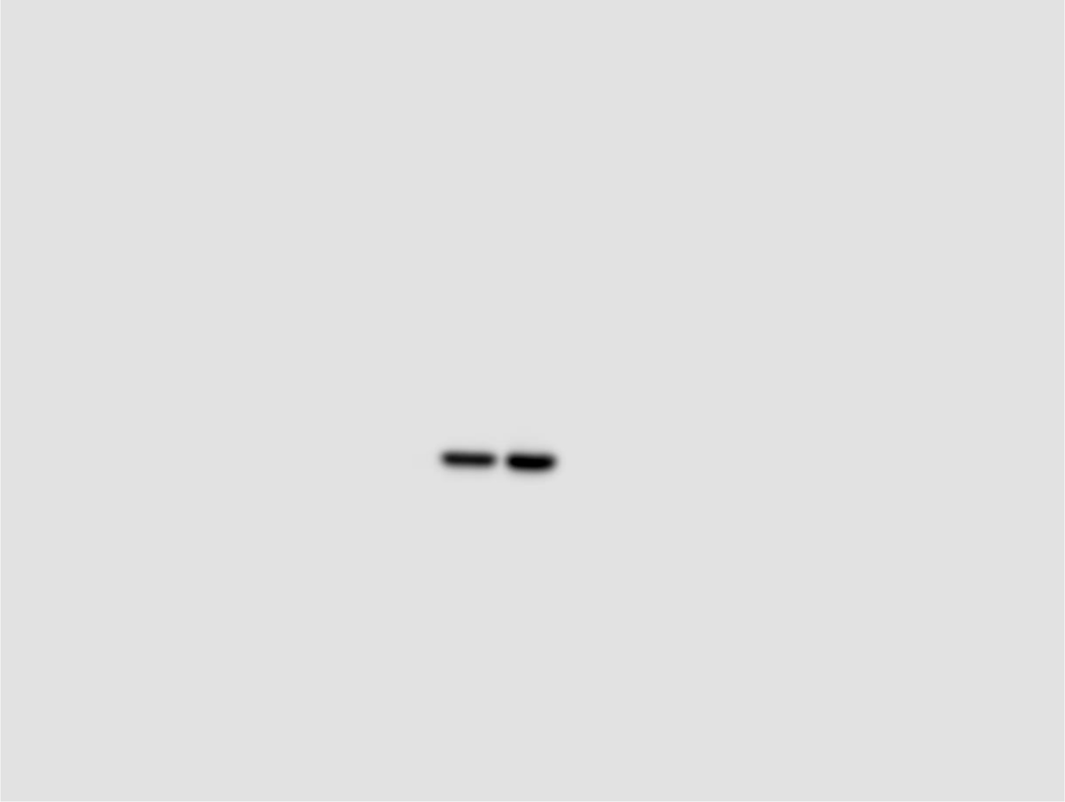

Figure 2C
A2780_pub

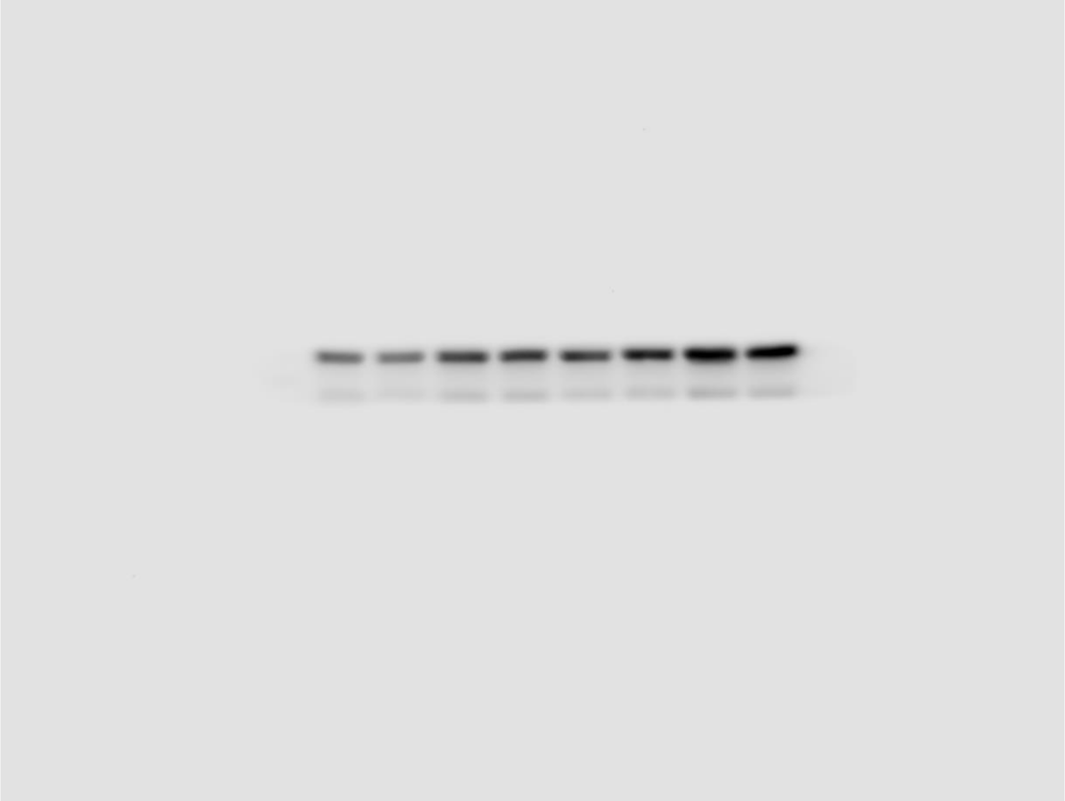

GAPDH A2780_pub

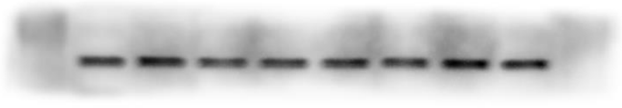

GAPDH ov_pub

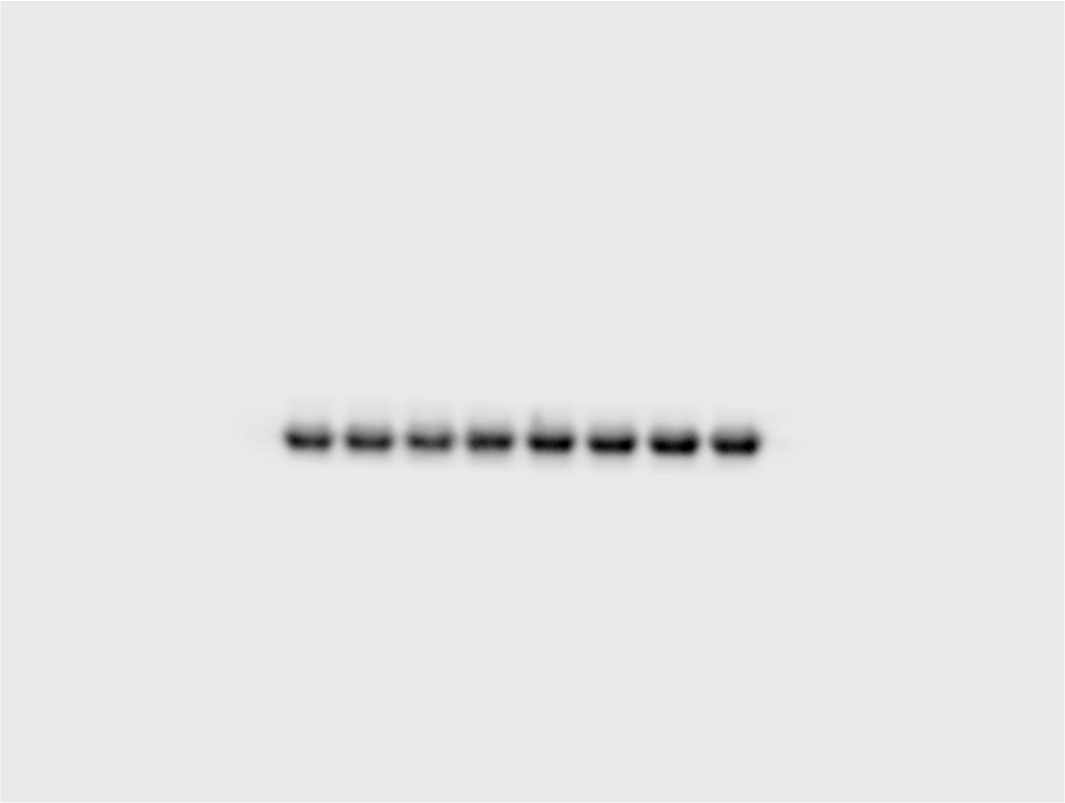

OVCAR-3_pub

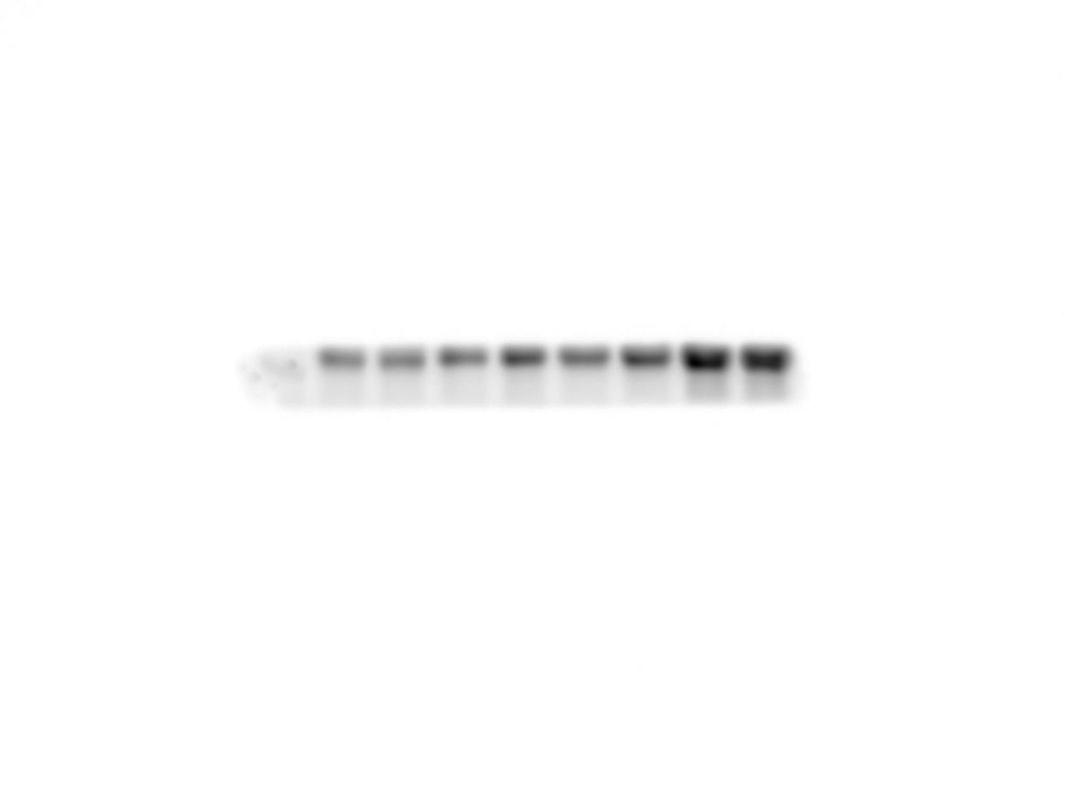

gapdh SKOV3_pub

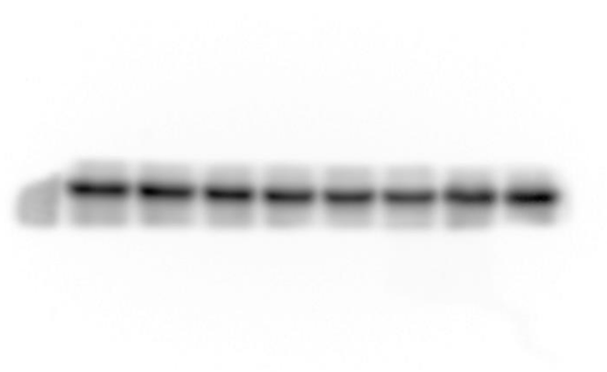

SKOV3_pub

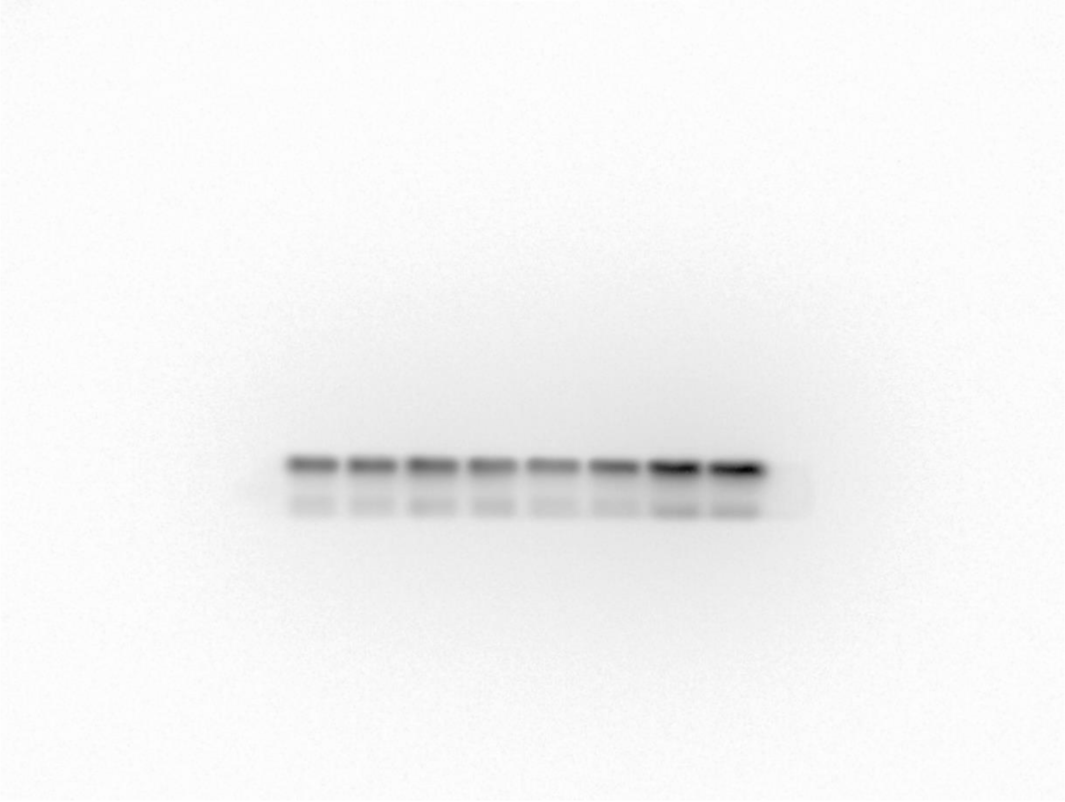

Figure 3C

FAM_pub

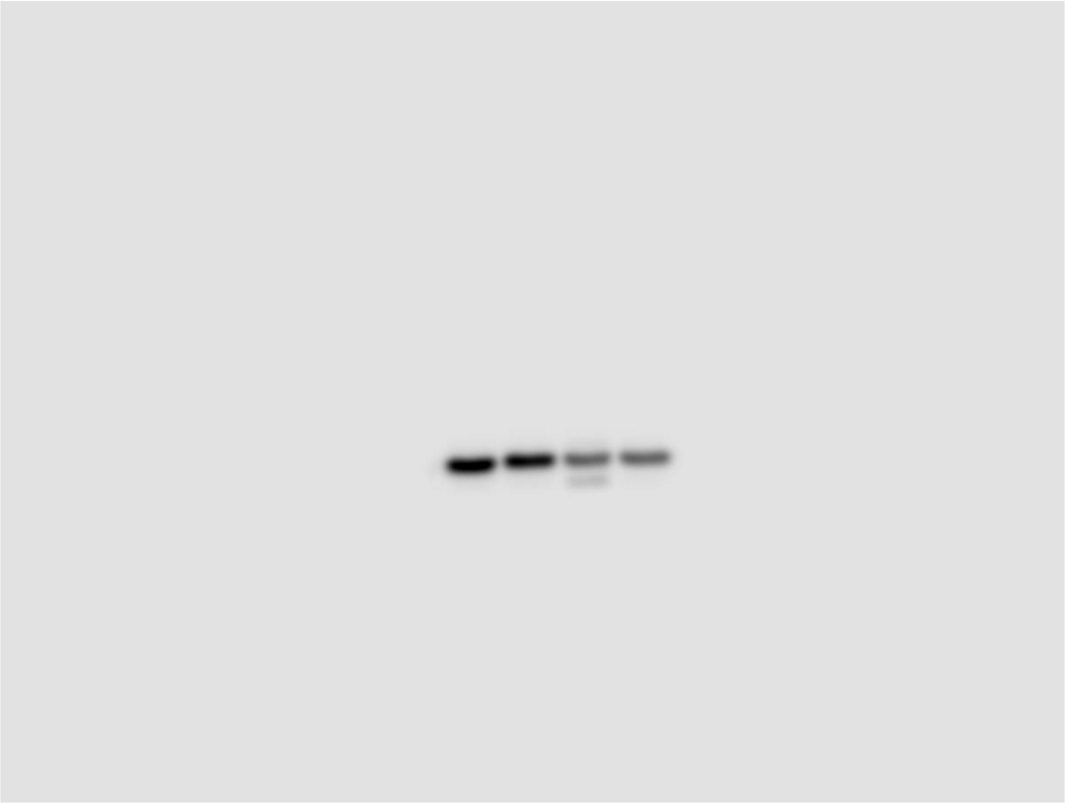

gapdh_pub

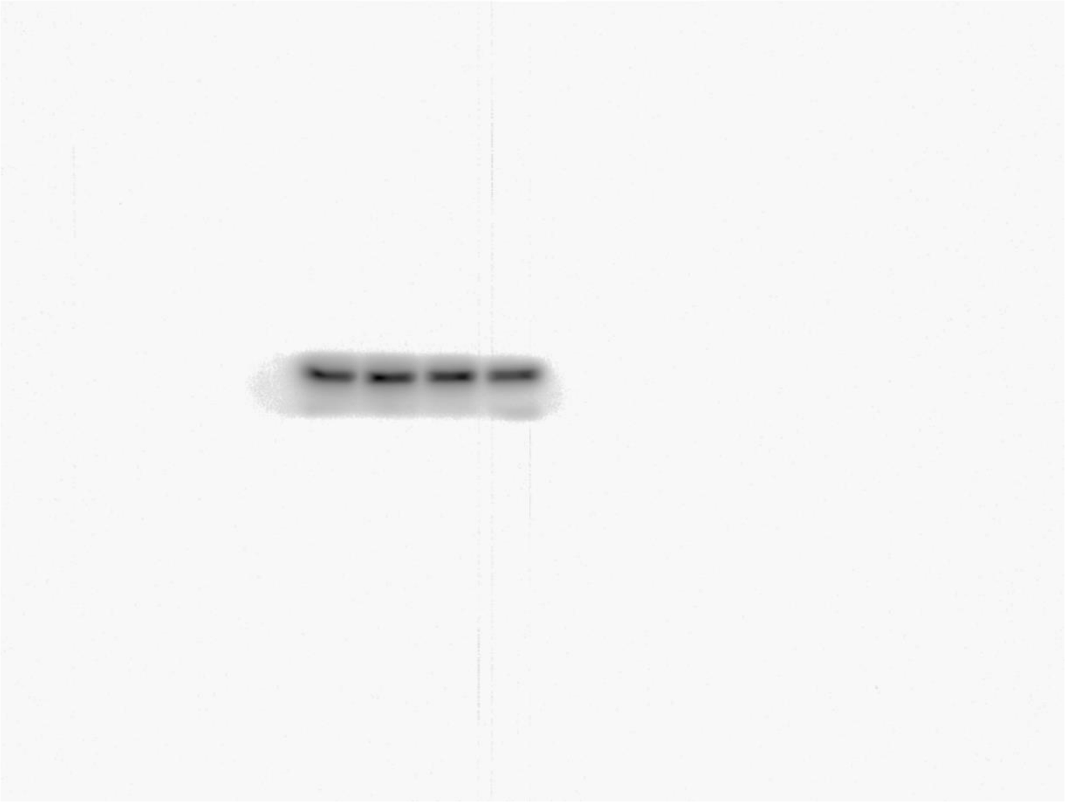

Ki67_pub

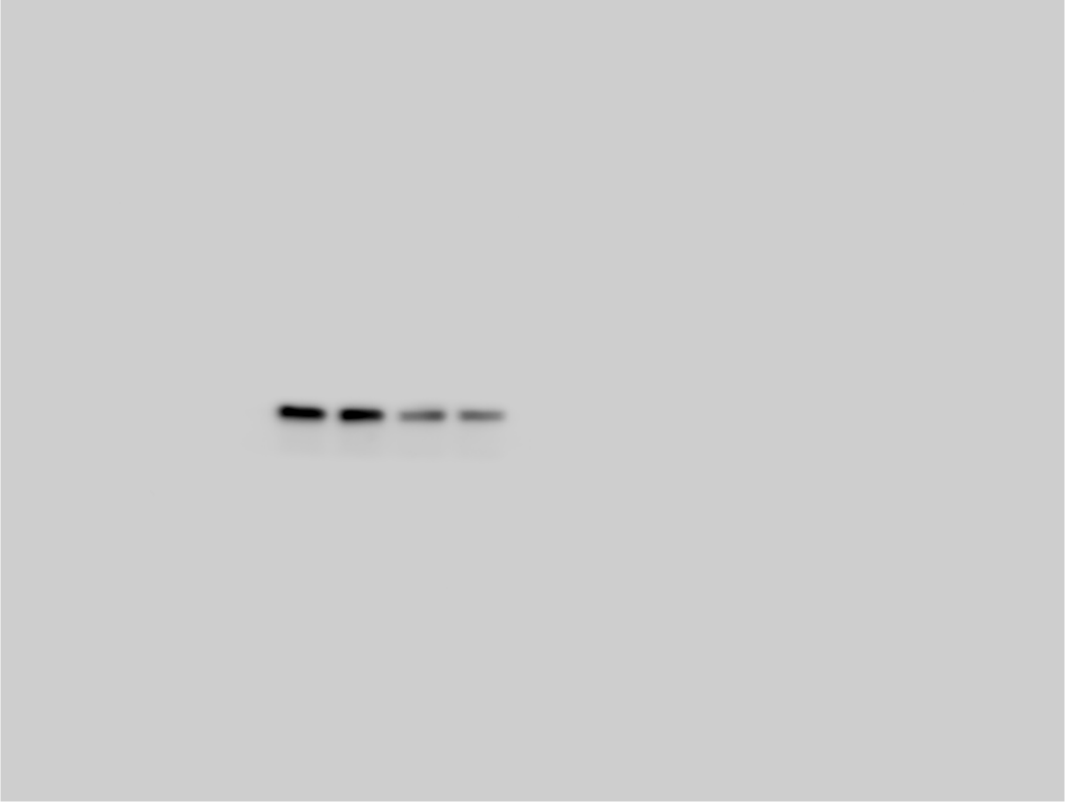

PCNA_pub

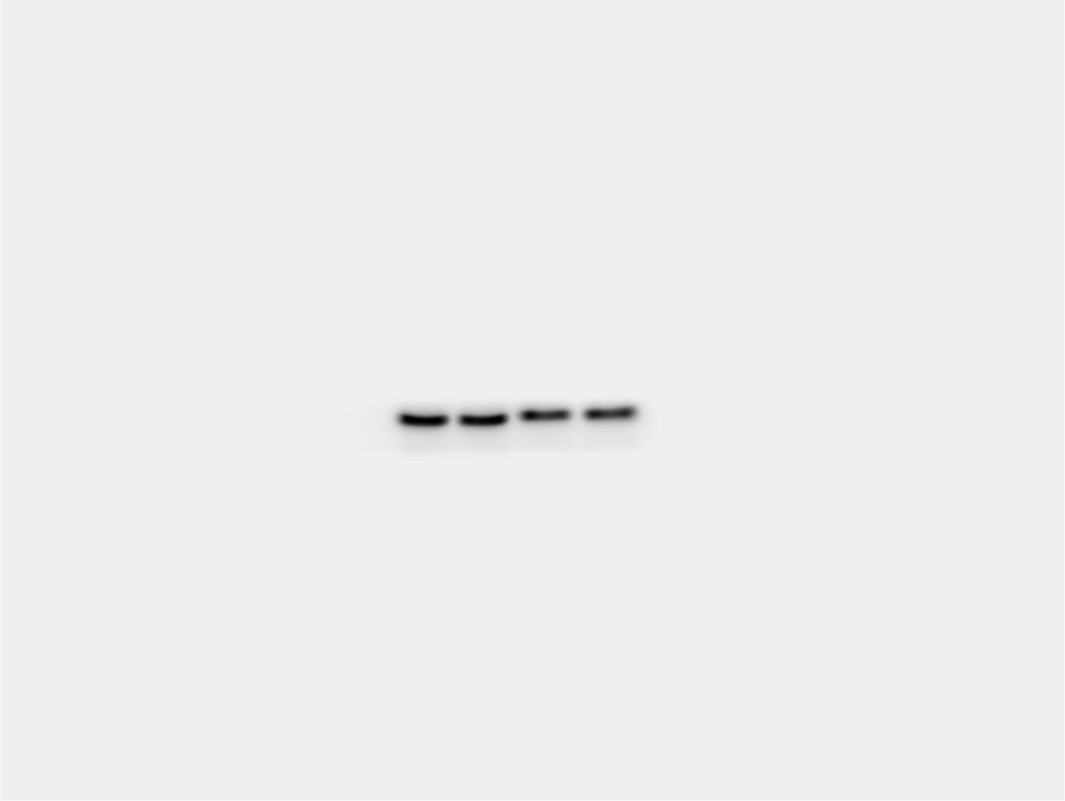

Figure 3I

E-CAD_pub

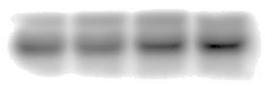

fam_pub

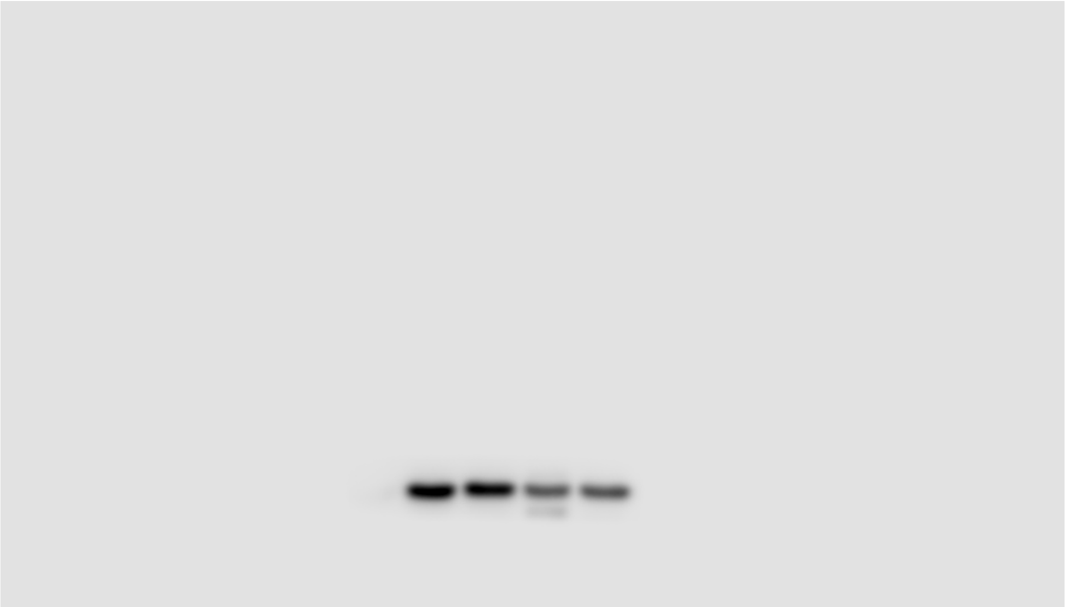

gapdh_pub

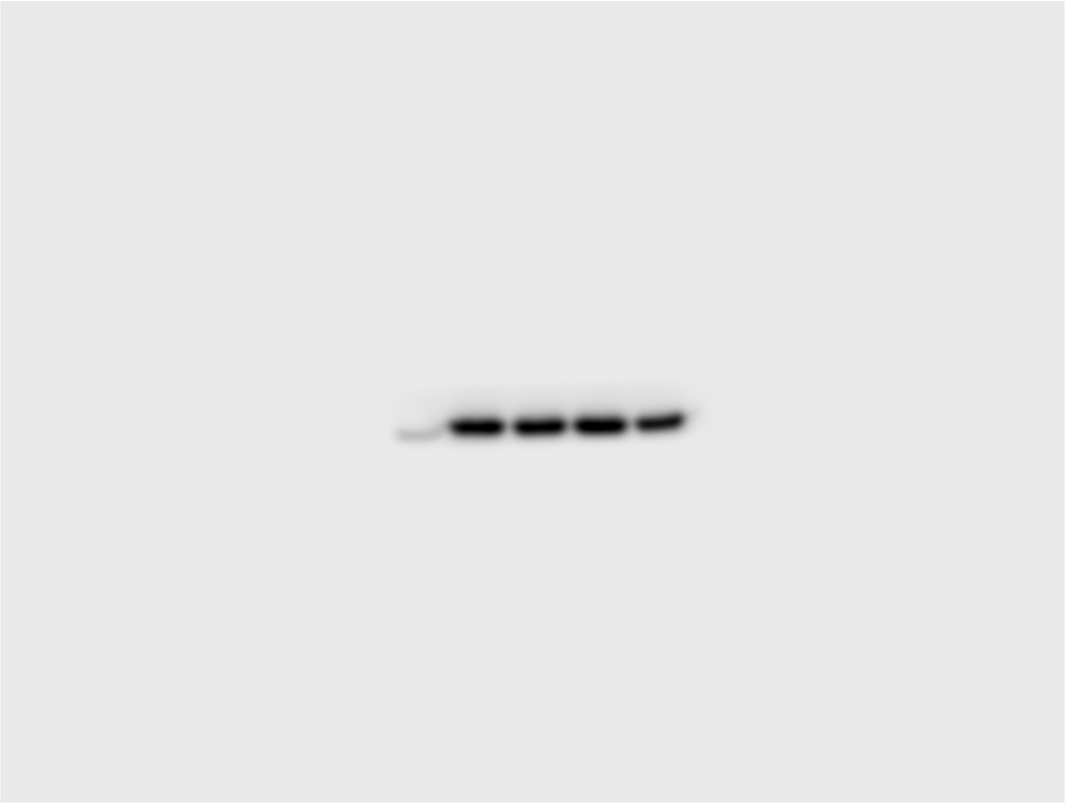

N-cad_pub

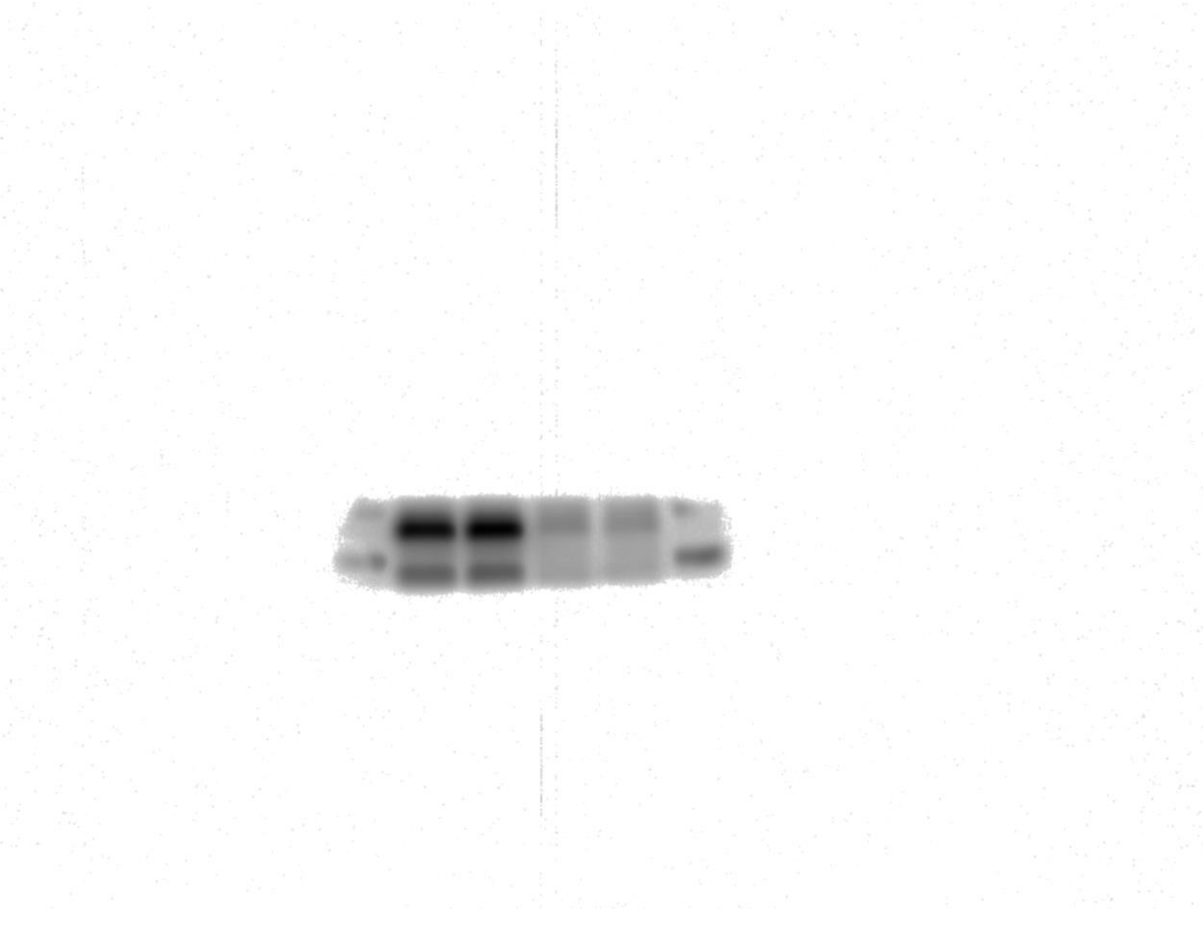

vimentin_pub

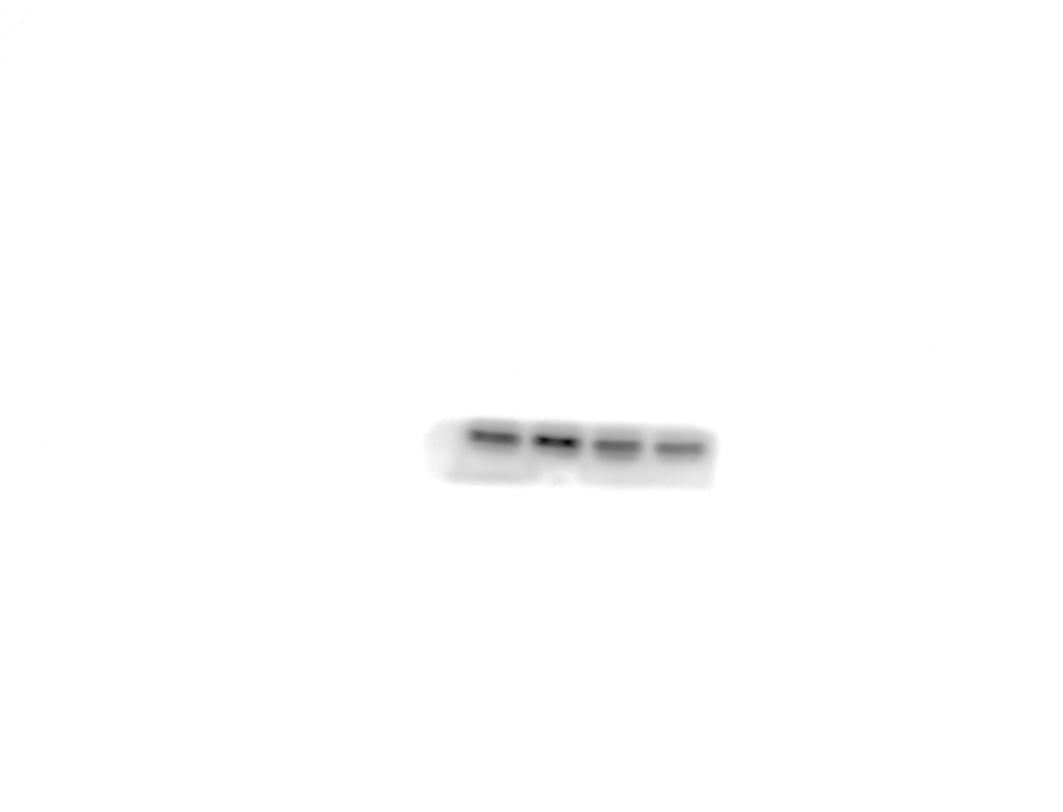

Figure 4D

A2780 AKT_pub

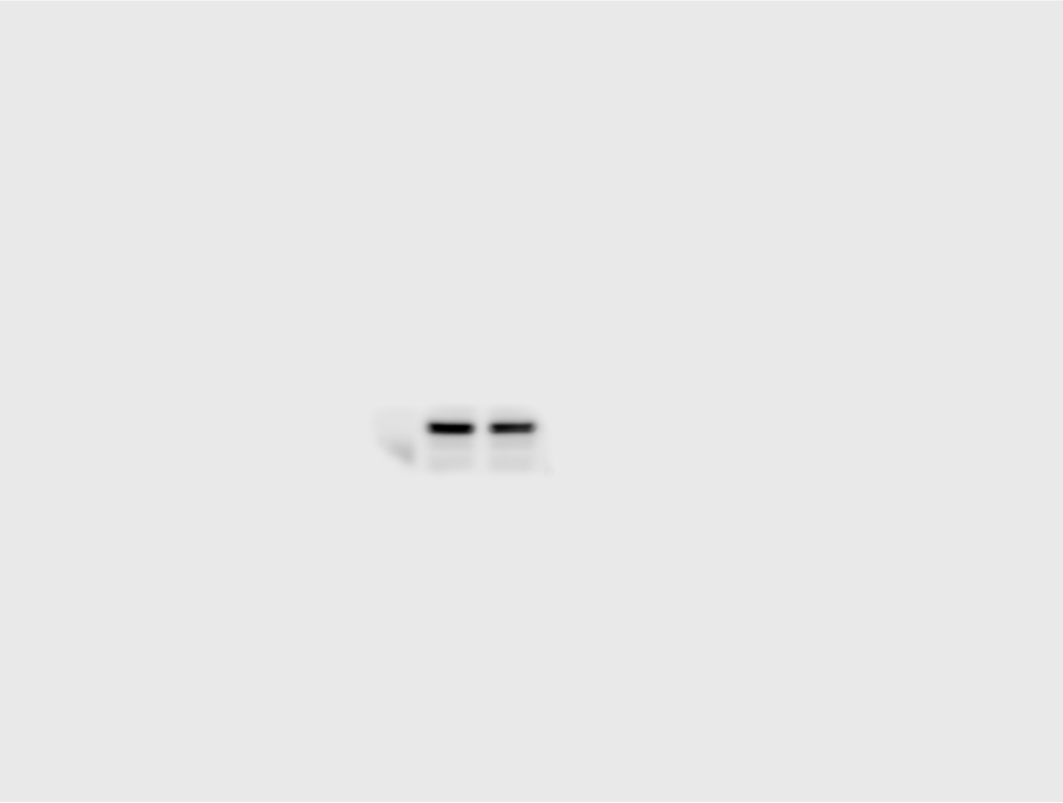

A2780 FAM_pub

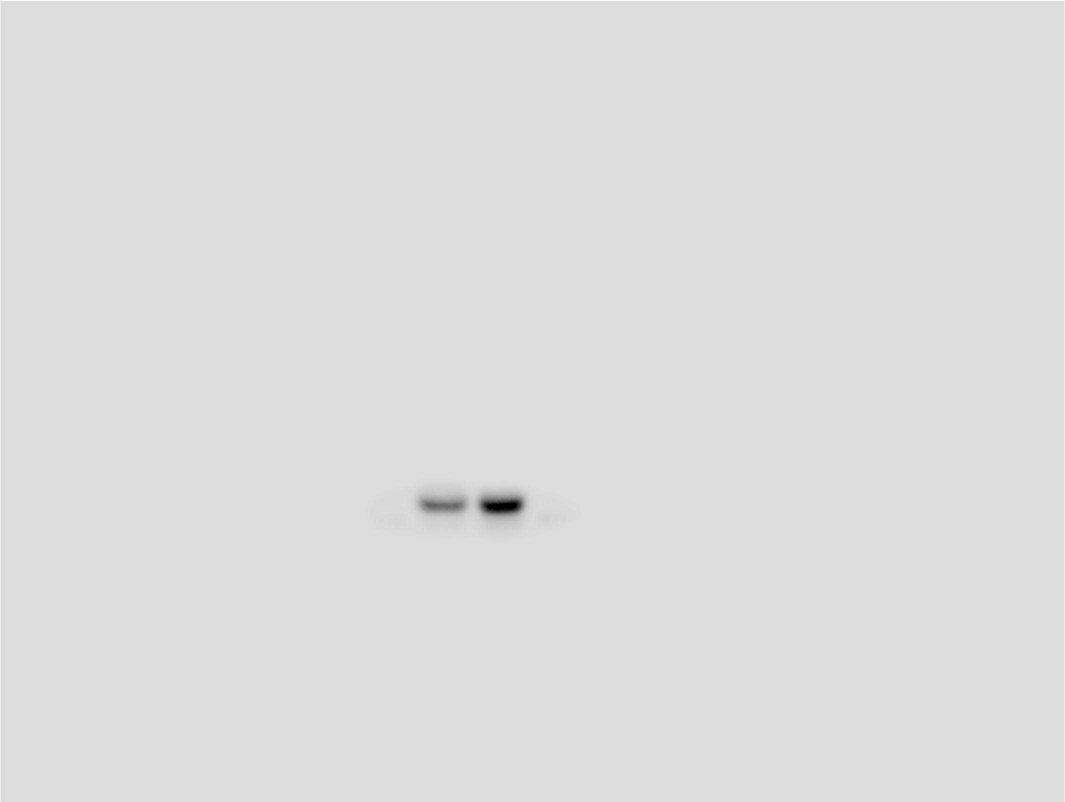

A2780 gapdh_pub

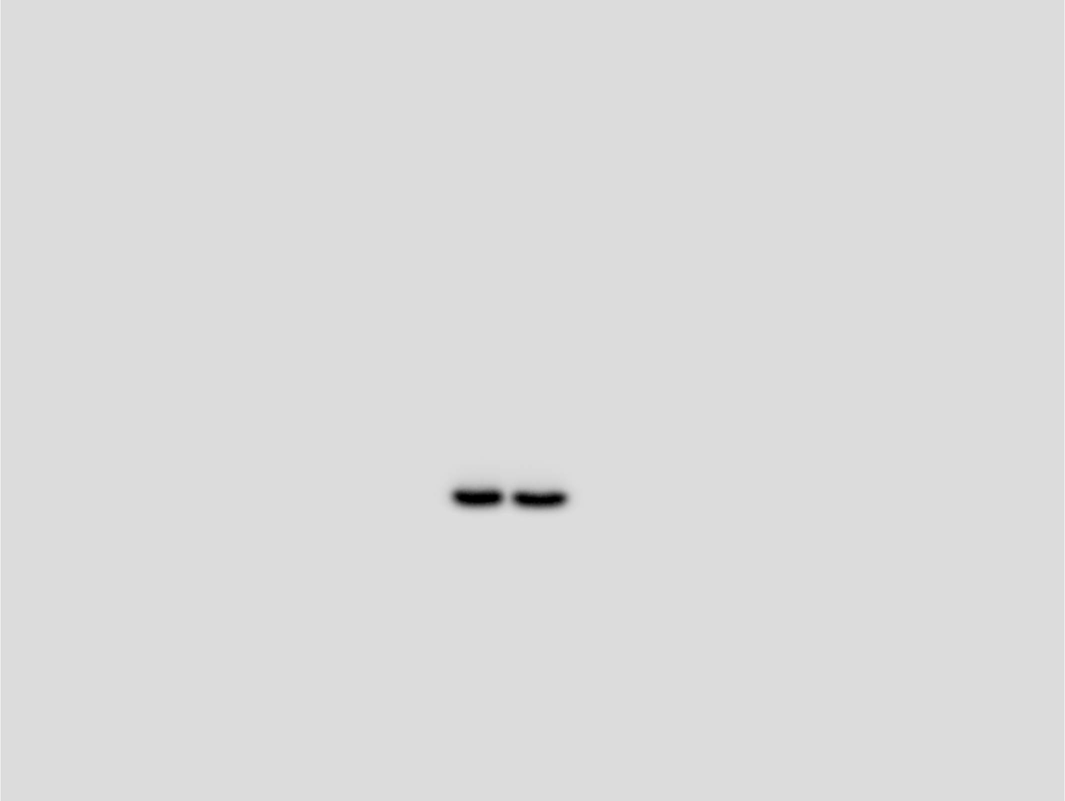

A2780 mtor_pub

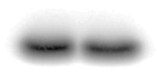

A2780 P-AKT_pub

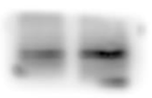

A2780 PI3K_pub

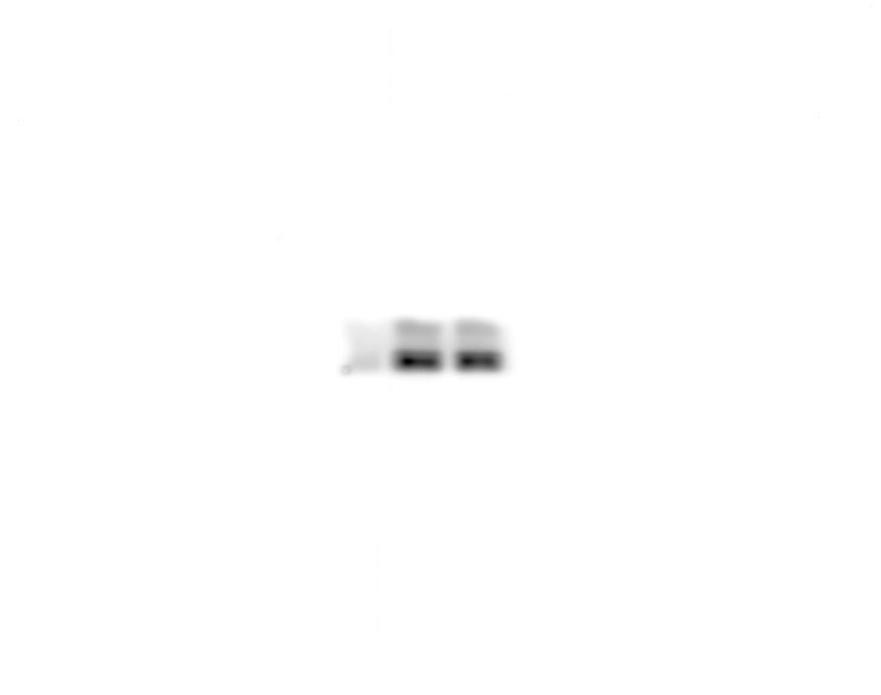

A2780 P-MTOR_pub

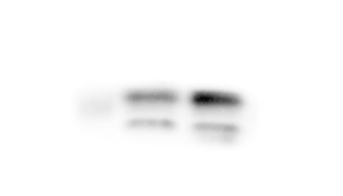

A2780 P-PI3K_pub

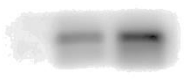

OVCAR-3 AKT_pub

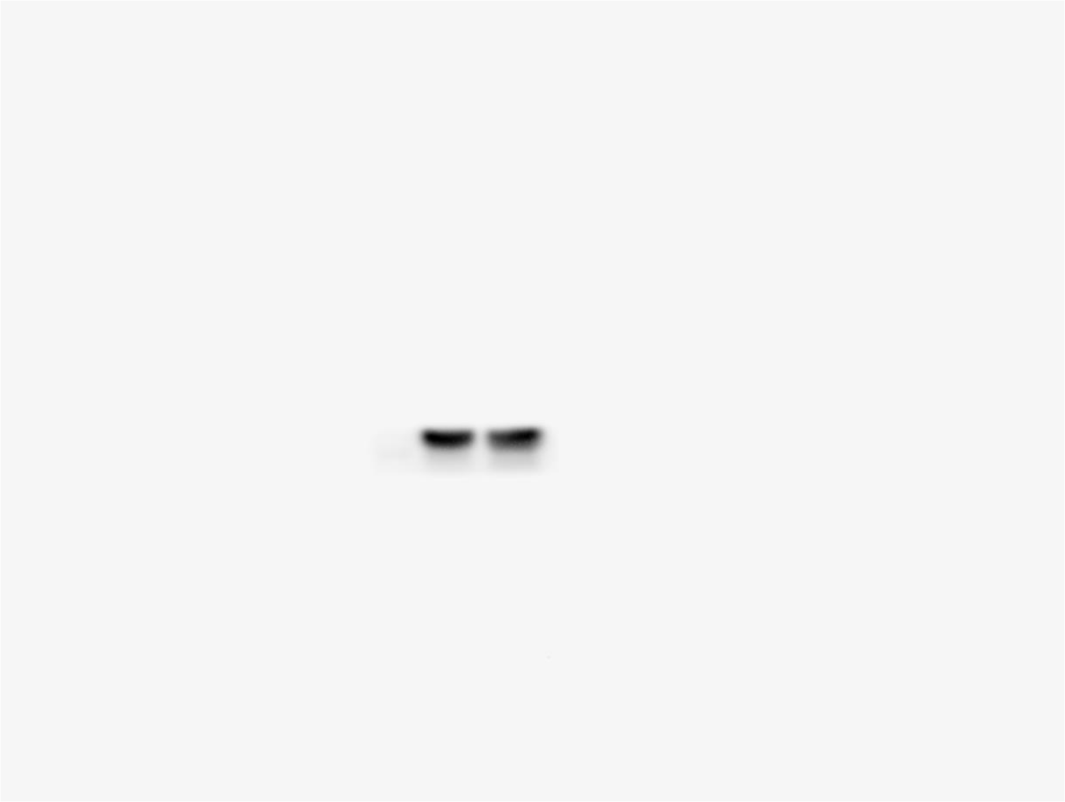

OVCAR-3 fam_pub

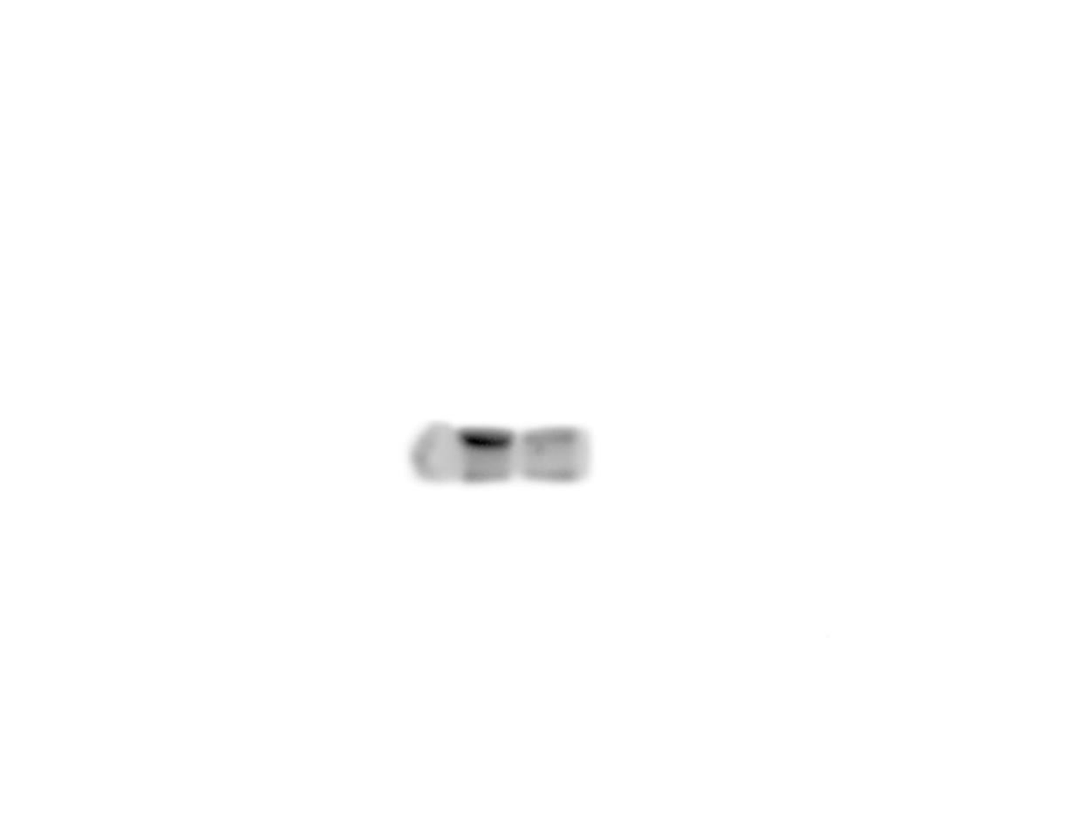

OVCAR-3 GAPDH_pub

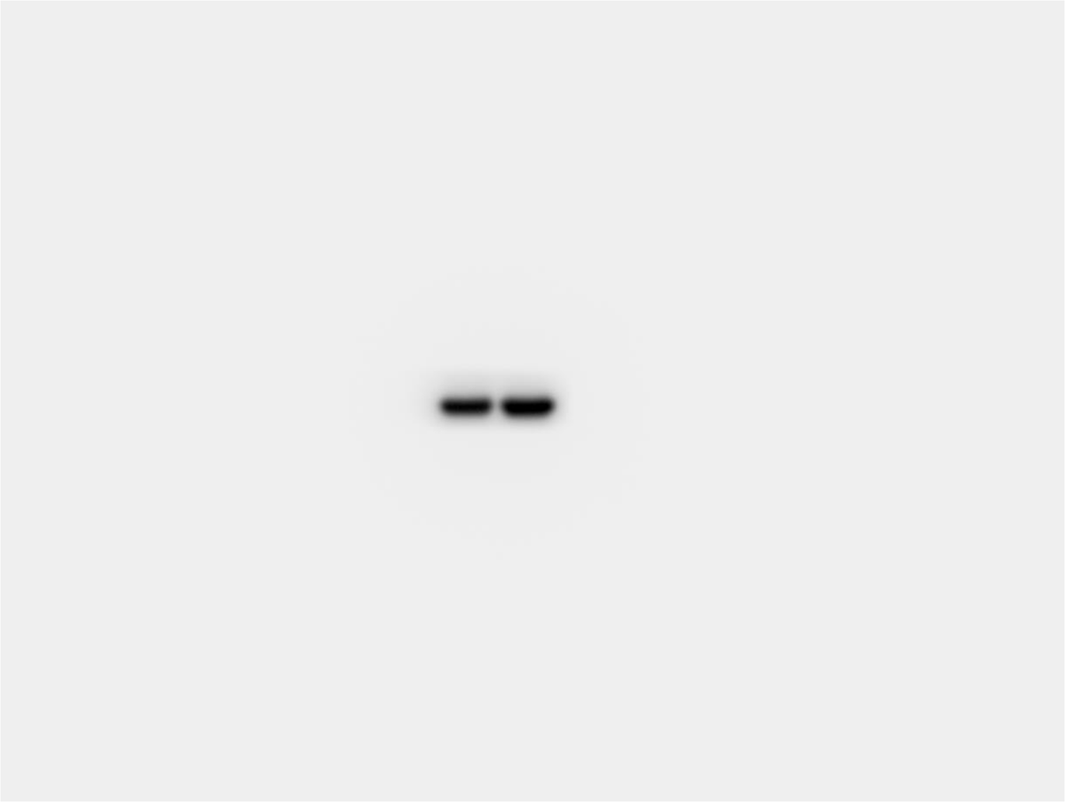

OVCAR-3 mtor_pub

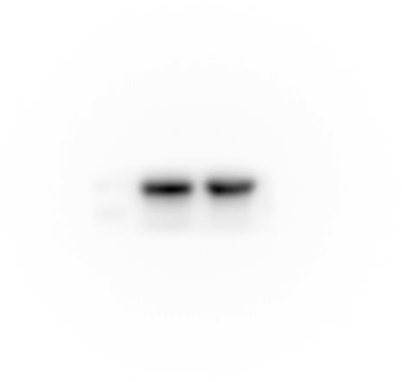

OVCAR-3 p-akt_pub

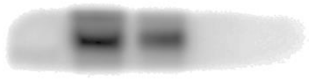

OVCAR-3 pi3k_pub

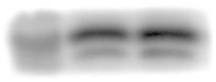

OVCAR-3 p-mtor_pub

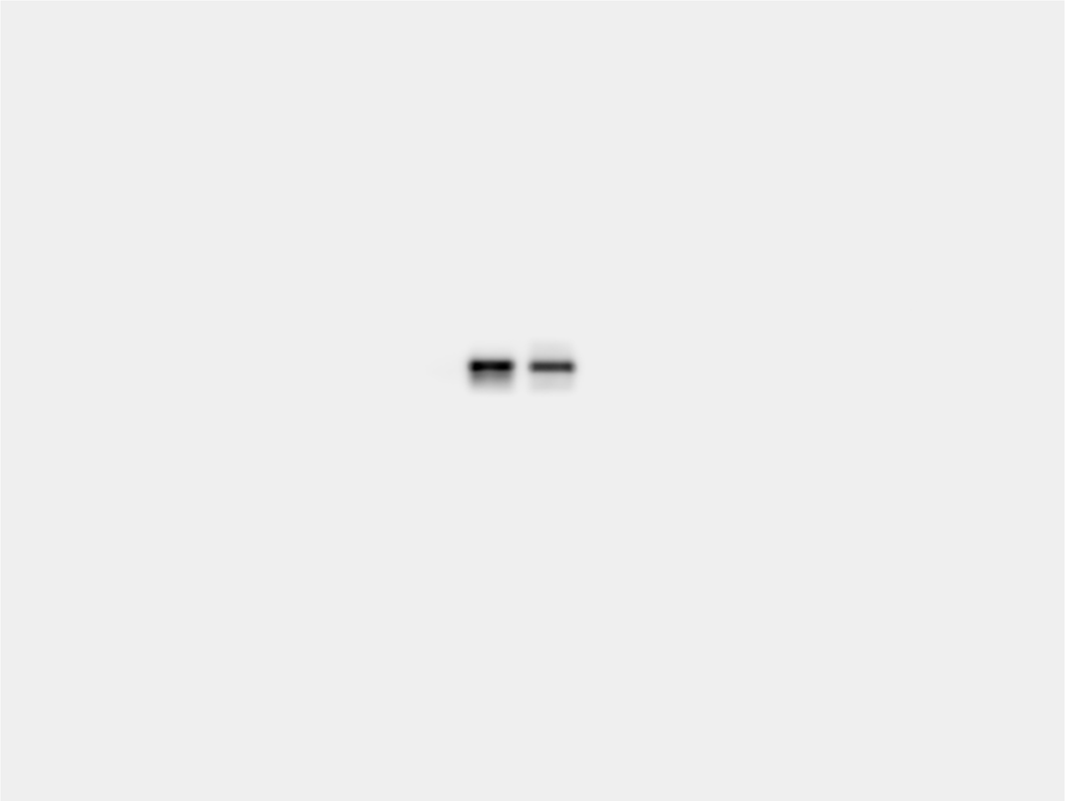

OVCAR-3 p-pi3k_pub

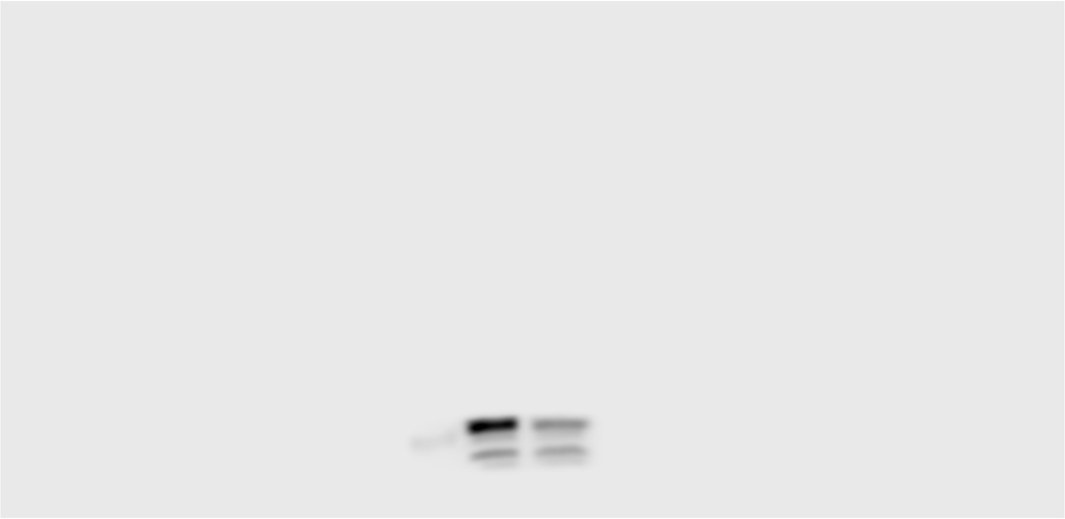

SKOV3 AKT_pub

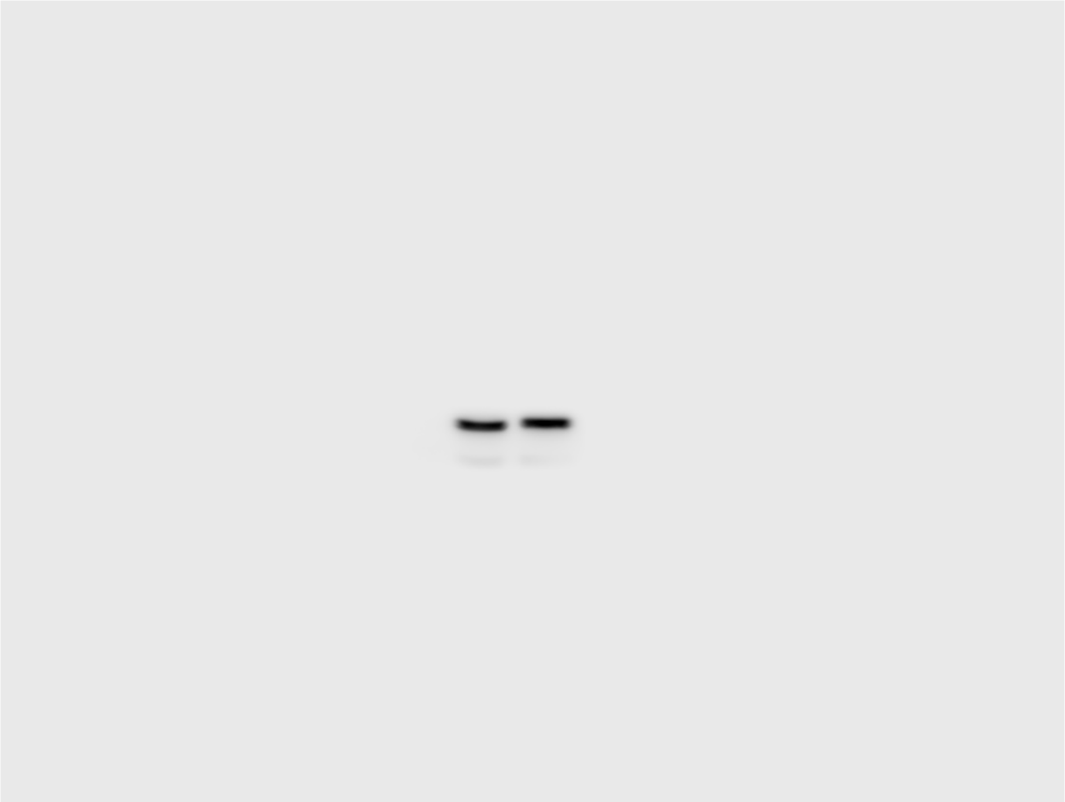

SKOV3 gapdh_pub

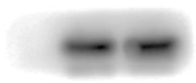

SKOV3 MTOR_pub

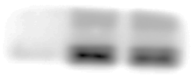

SKOV3 P-AKT_pub

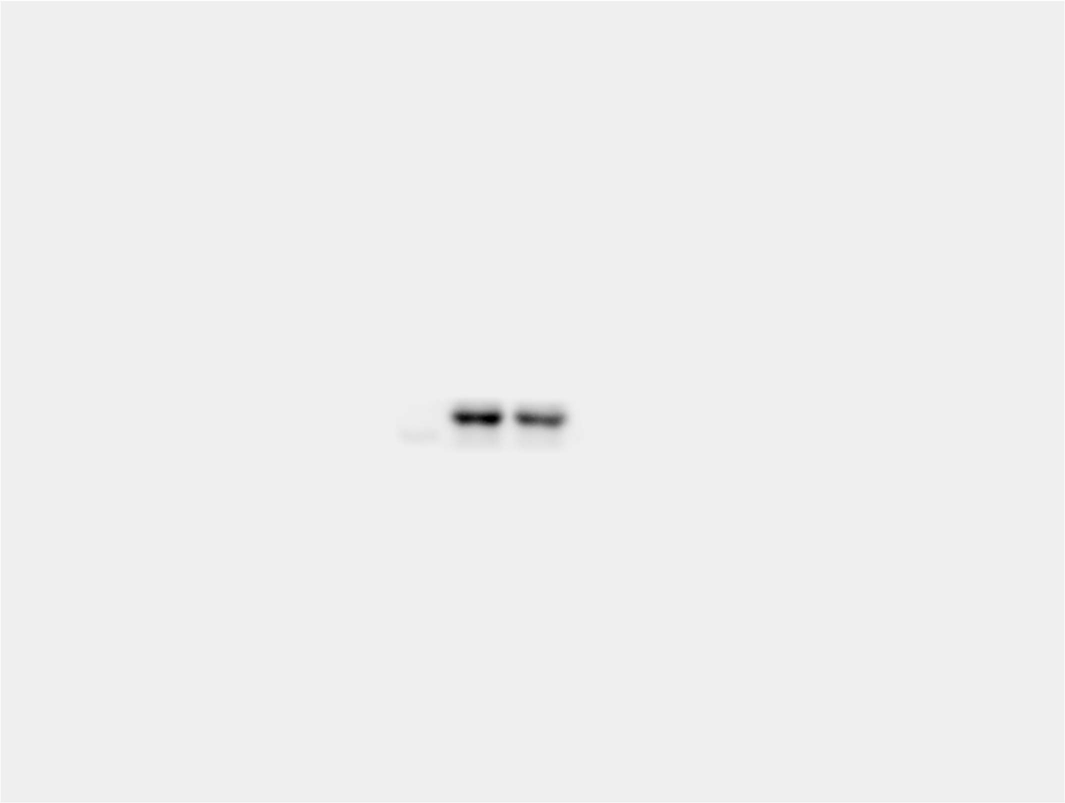

SKOV3 PI3K_pub

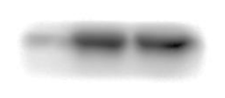

SKOV3 p-mtor_pub

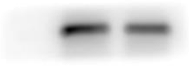

SKOV3 P-PI3K_pub

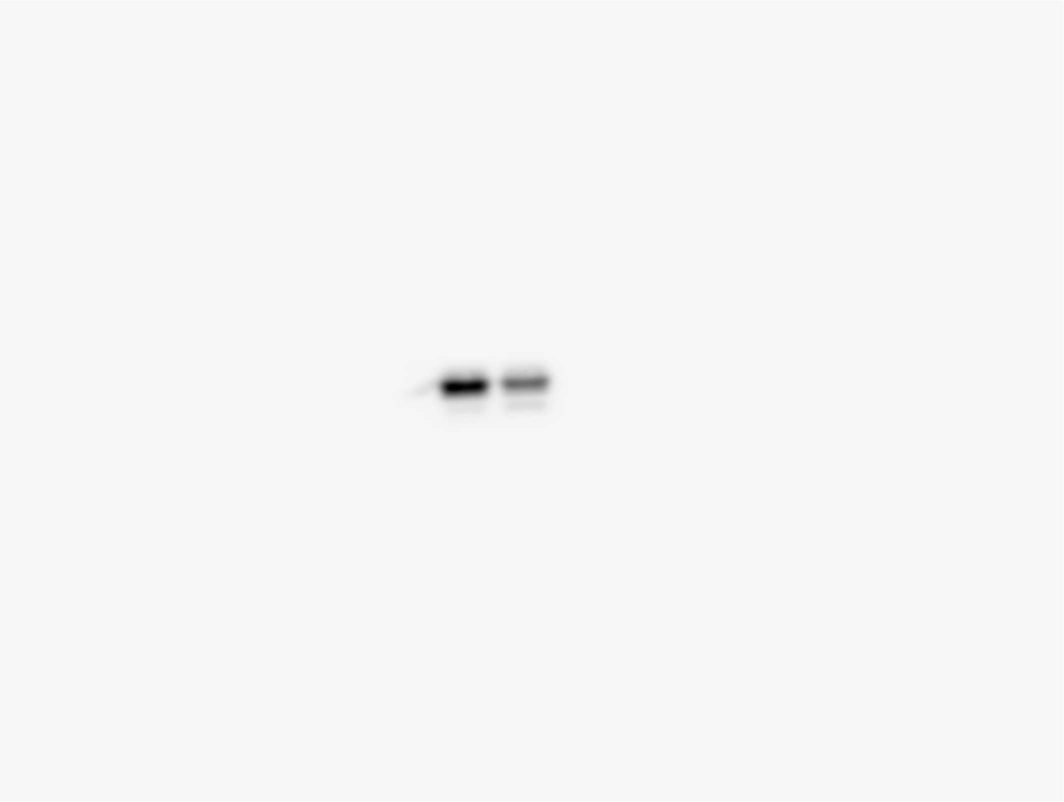

SKOV3 SKOFAM_pub

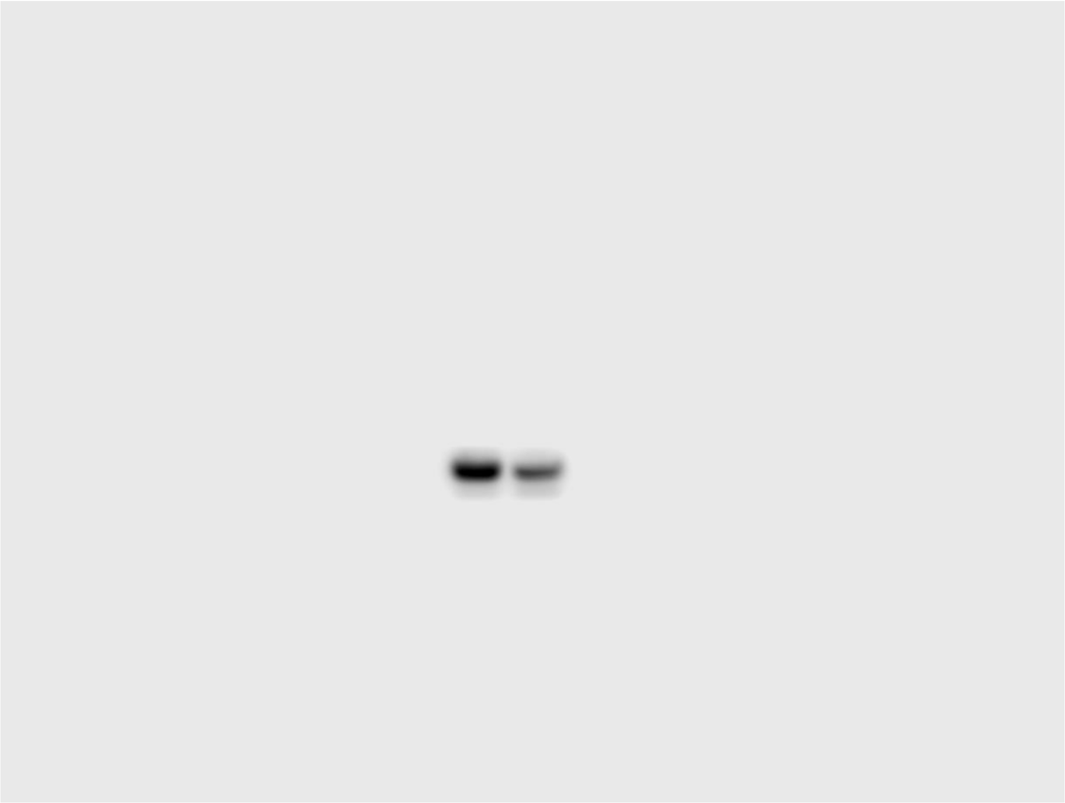

Figure 4E

A2780 AKT_pub

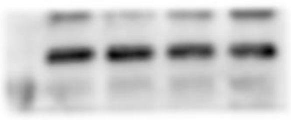

A2780 GAPDH_pub

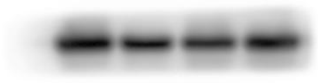

A2780 mtor_pub

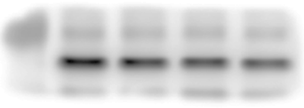

A2780 P-AKT_pub

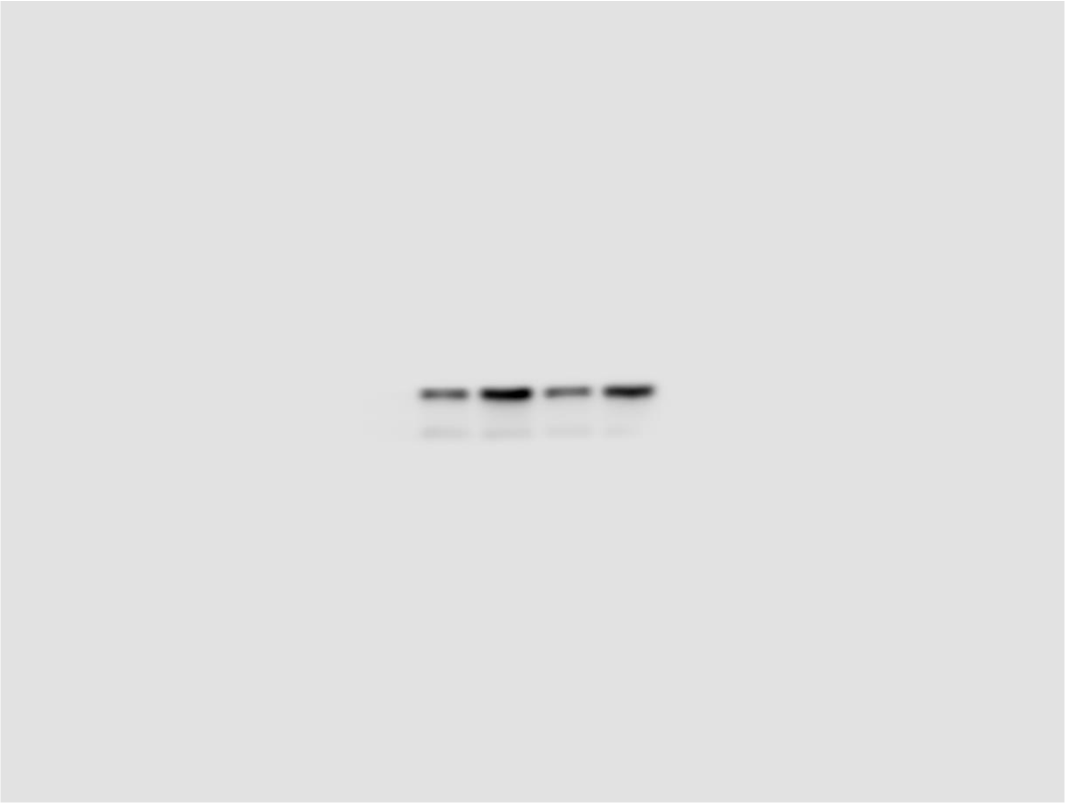

A2780 PI3K_pub

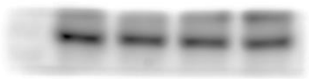

A2780 P-mtor_pub

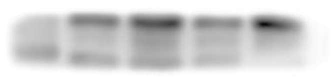

A2780 P-PI3K_pub

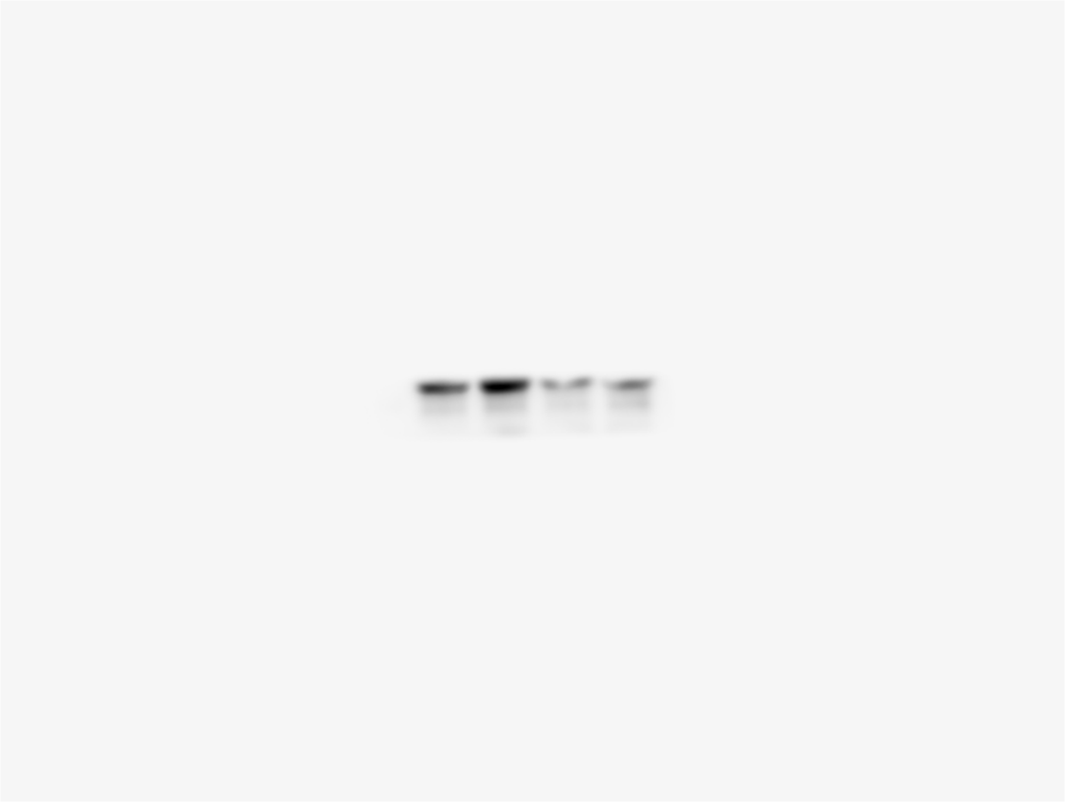

OVCAR-3 AKT_pub

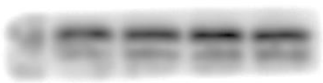

OVCAR-3 GAPDH_pub

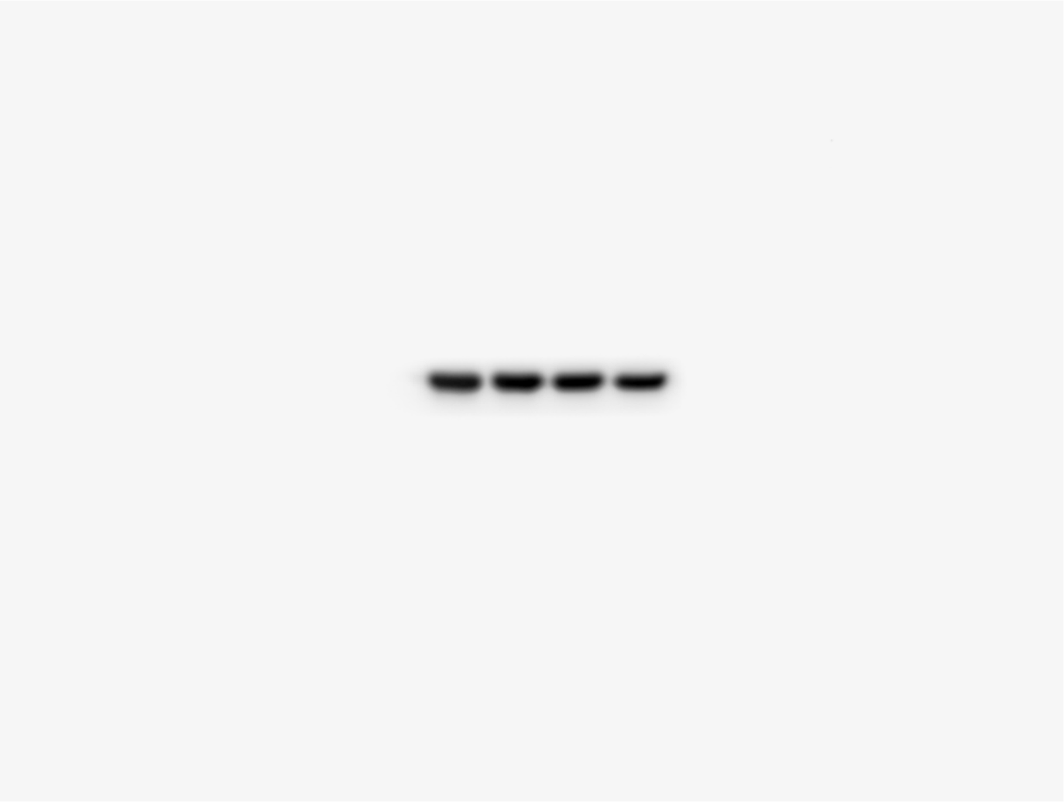

OVCAR-3 MTOR_pub

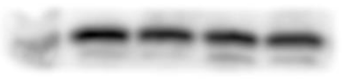

OVCAR-3 P-AKT_pub

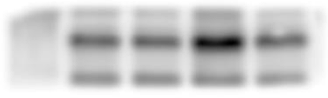

OVCAR-3 PI3K_pub

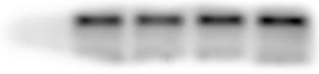

OVCAR-3 P-MTOR_pub

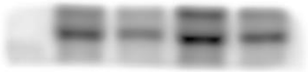

OVCAR-3 P-PI3K_pub

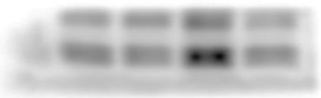

SKOV3 AKT_pub

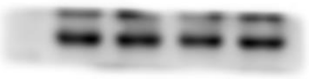

SKOV3 gapdh_pub

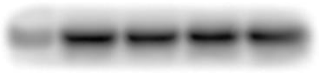

SKOV3 mtor_pub

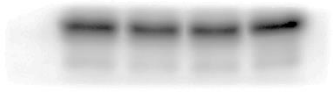

SKOV3 P-AKT_pub

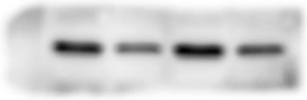

SKOV3 PI3K_pub

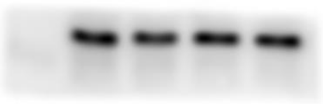

SKOV3 P-MTOR_pub

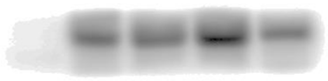

SKOV3 p-PI3K_pub

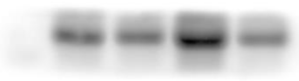

Figure 5C

A2780 GAPDH_pub

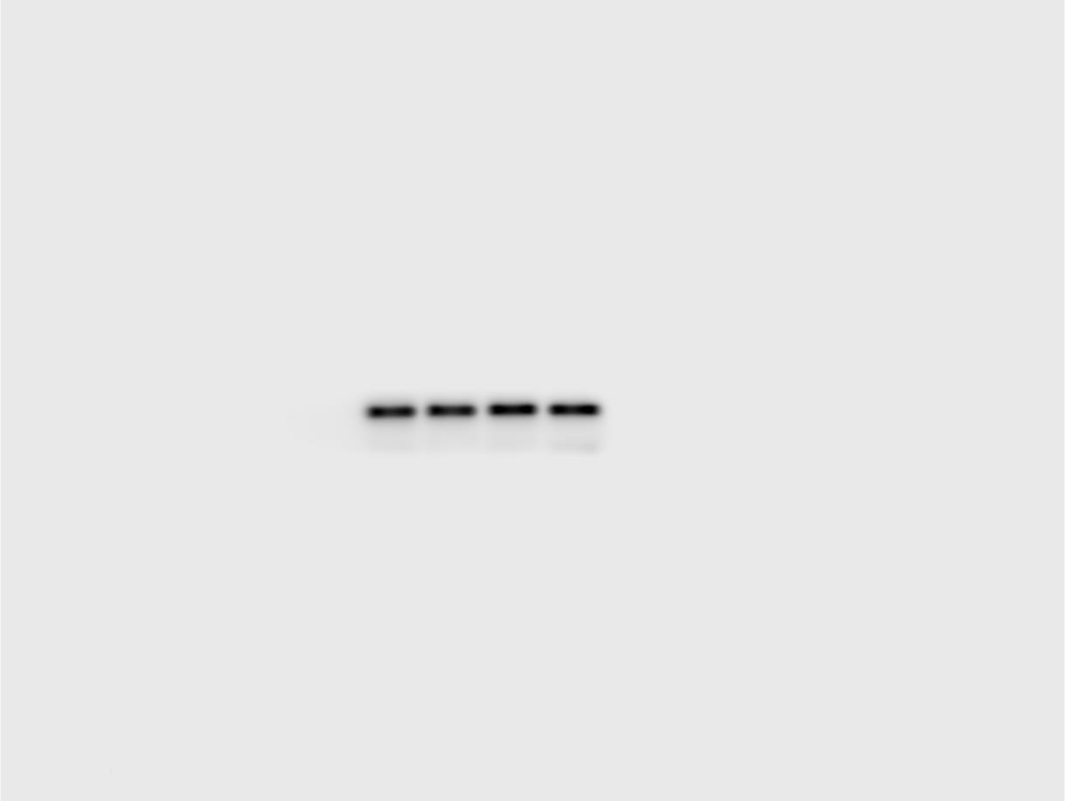

A2780 Ki67_pub

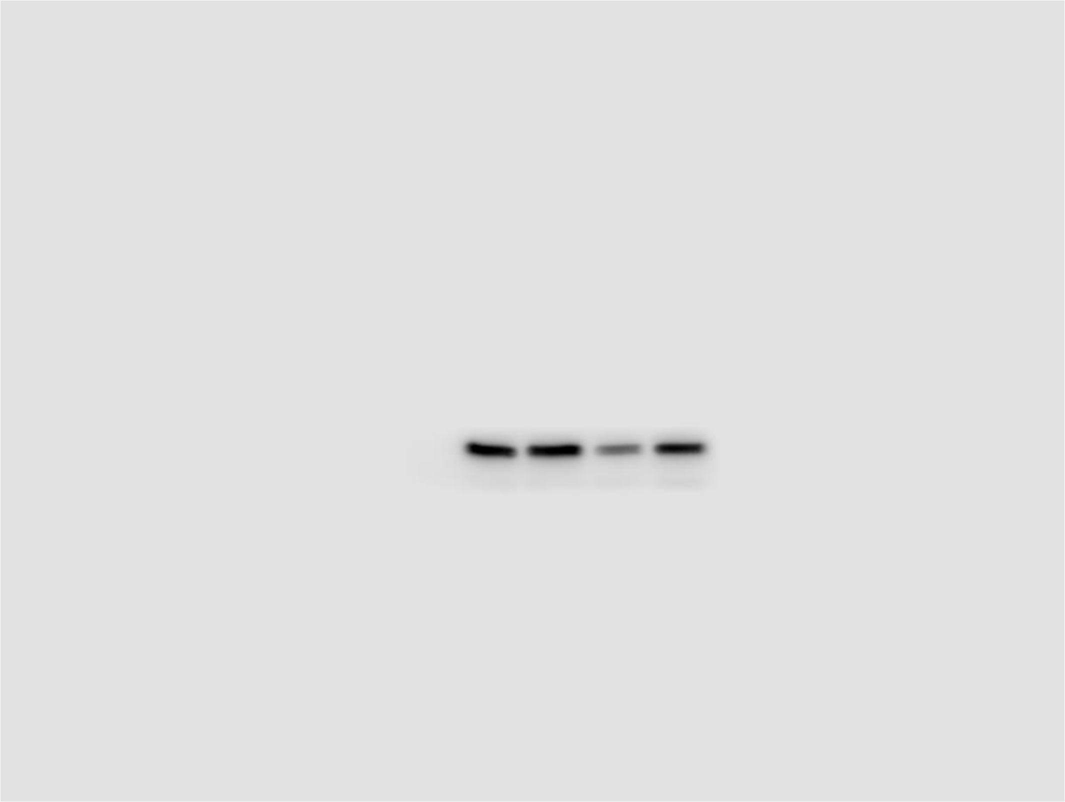

A2780 PCNA_pub

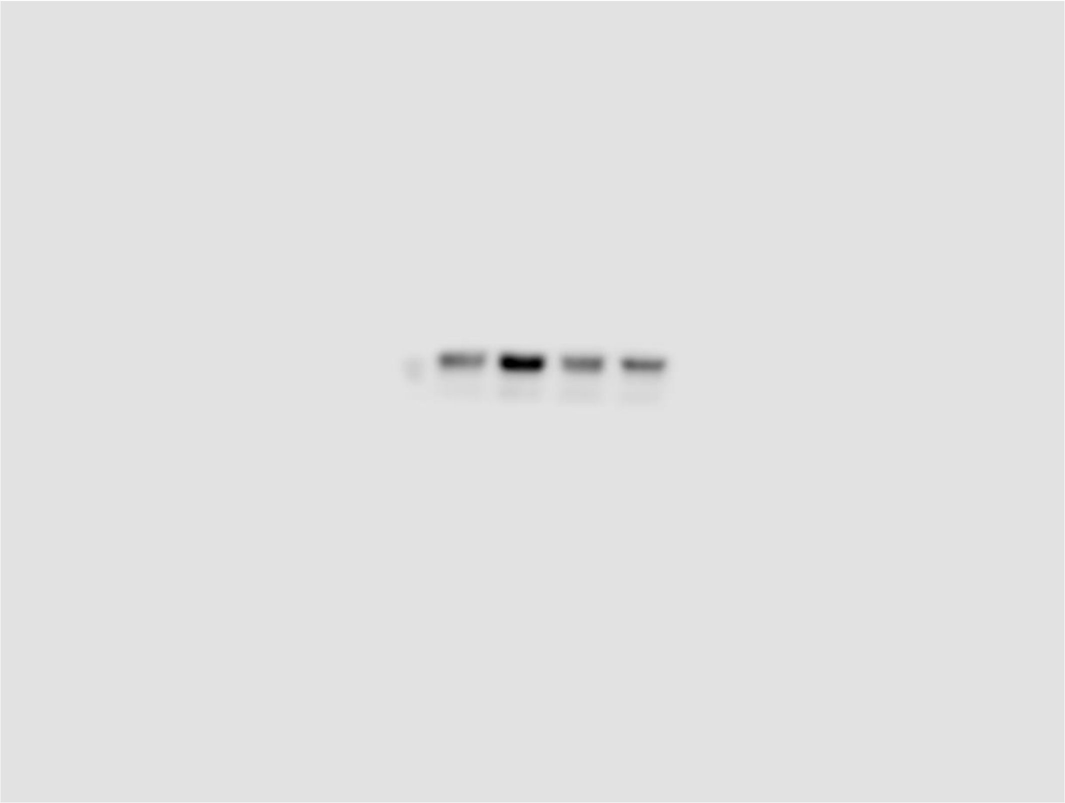

OVCAR-3 GAPDH_pub

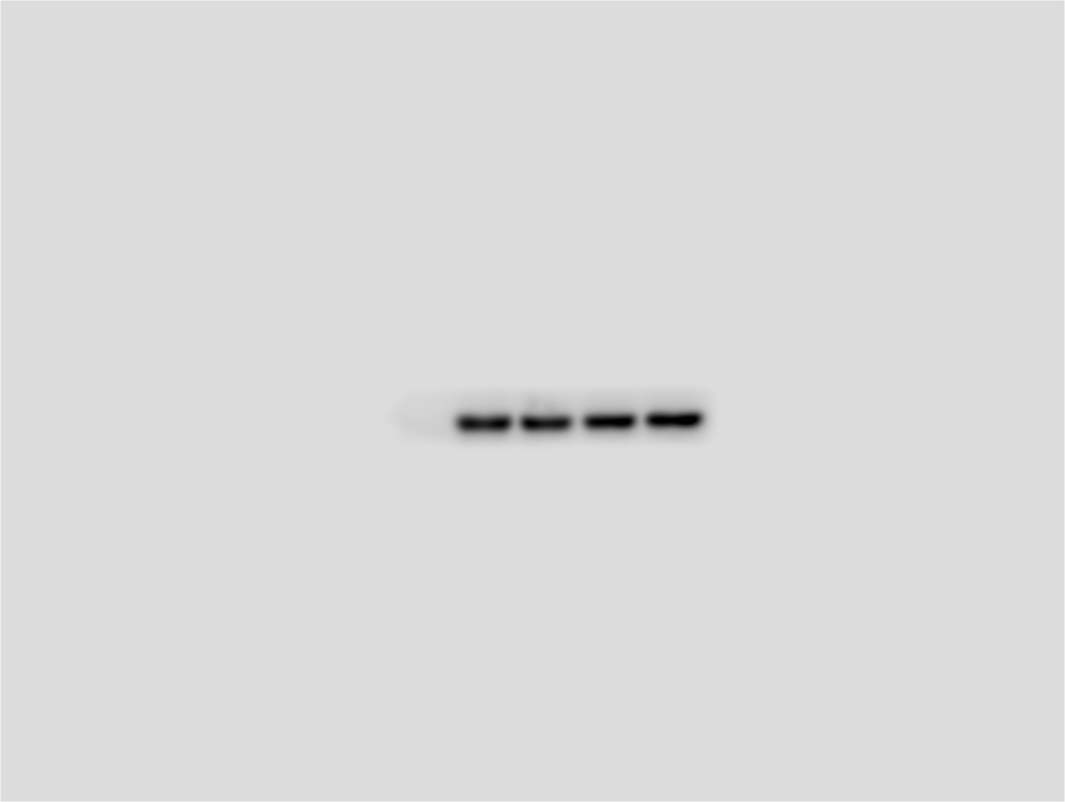

OVCAR-3 ki67_pub

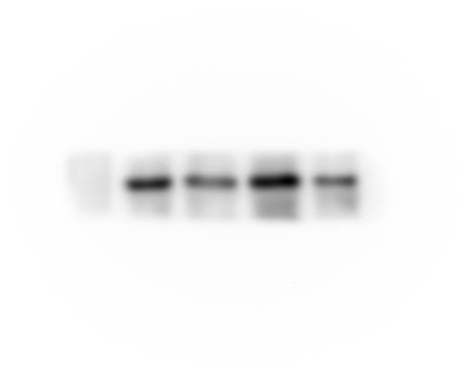

OVCAR-3 PCNA_pub

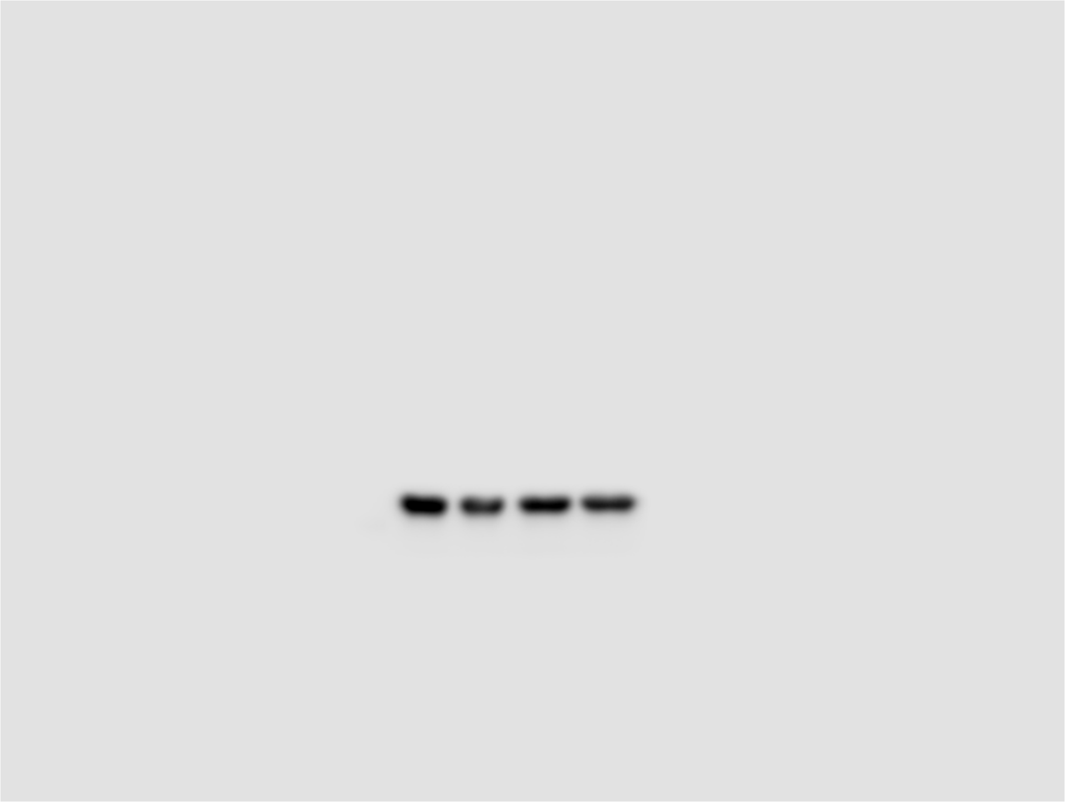

SKOV3 GAPDH_pub

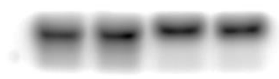

SKOV3 Ki67_pub

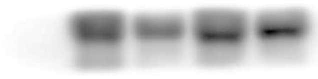

SKOV3 PCNA_pub

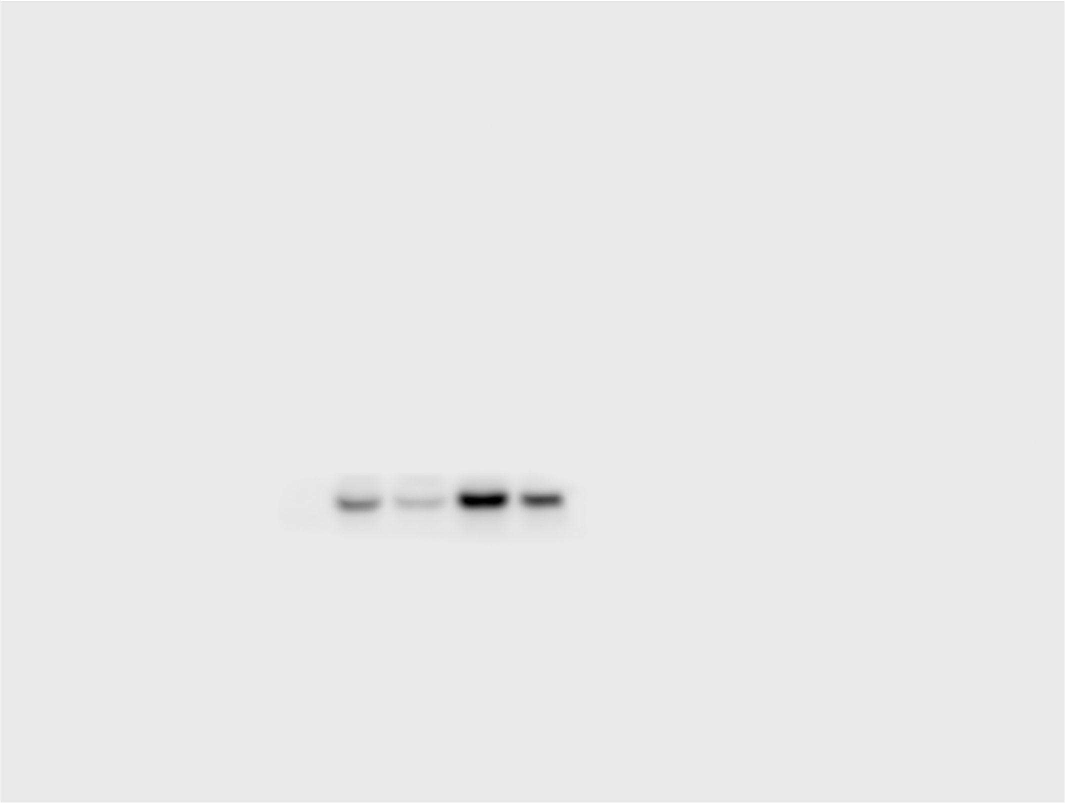

Figure 6B

A2780 E-cad_pub

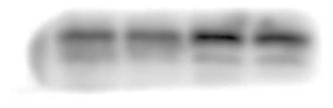

A2780 GAPDH_pub

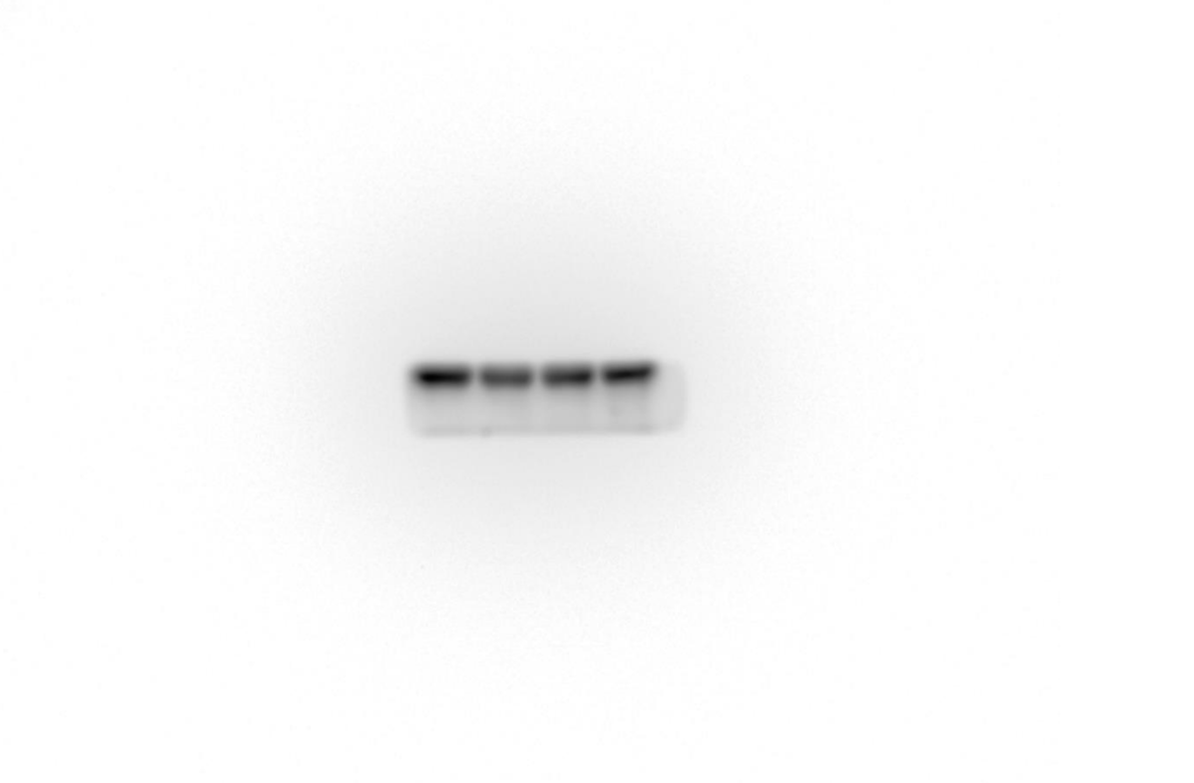

A2780 N-cad_pub

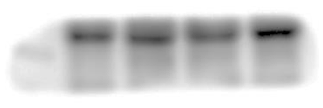

A2780 slug_pub

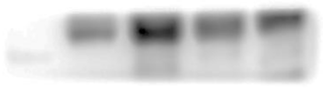

A2780 snail_pub

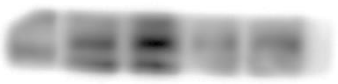

A2780 Twist_pub

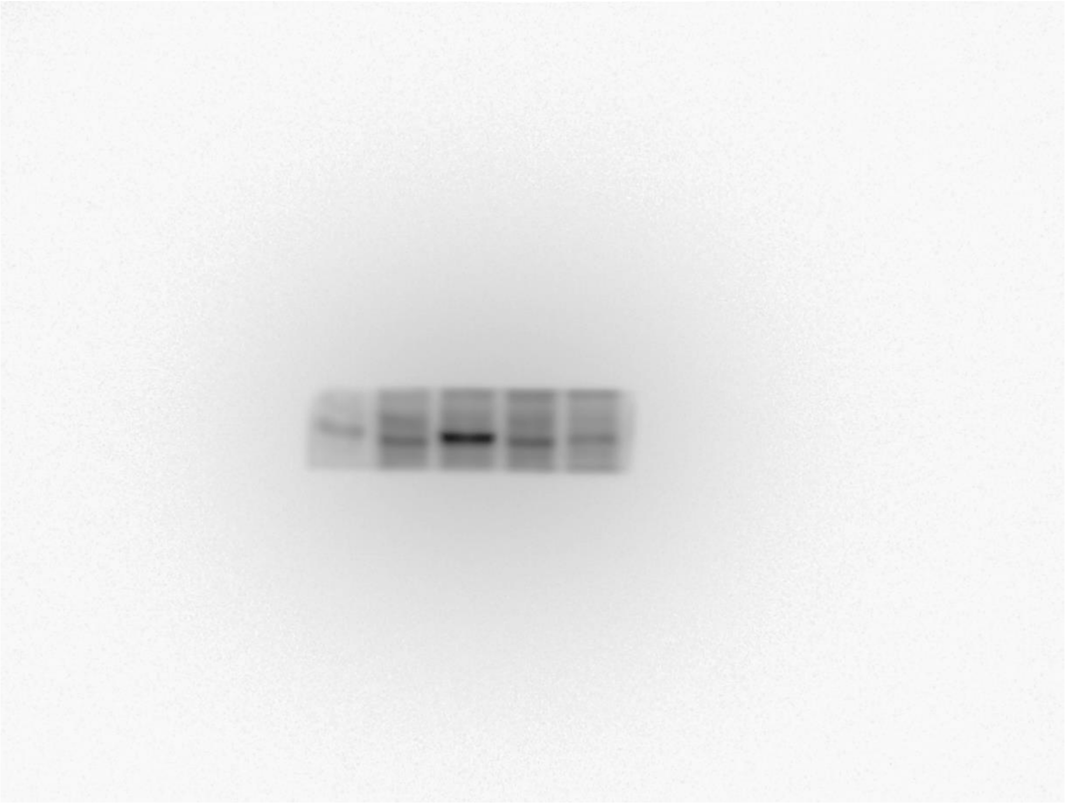

A2780 Vimentin_pub

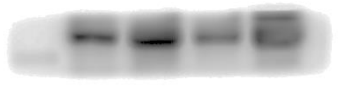

OVCAR-3 E-CAD_pub

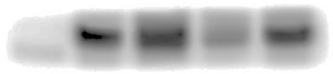

OVCAR-3 GAPDH_pub

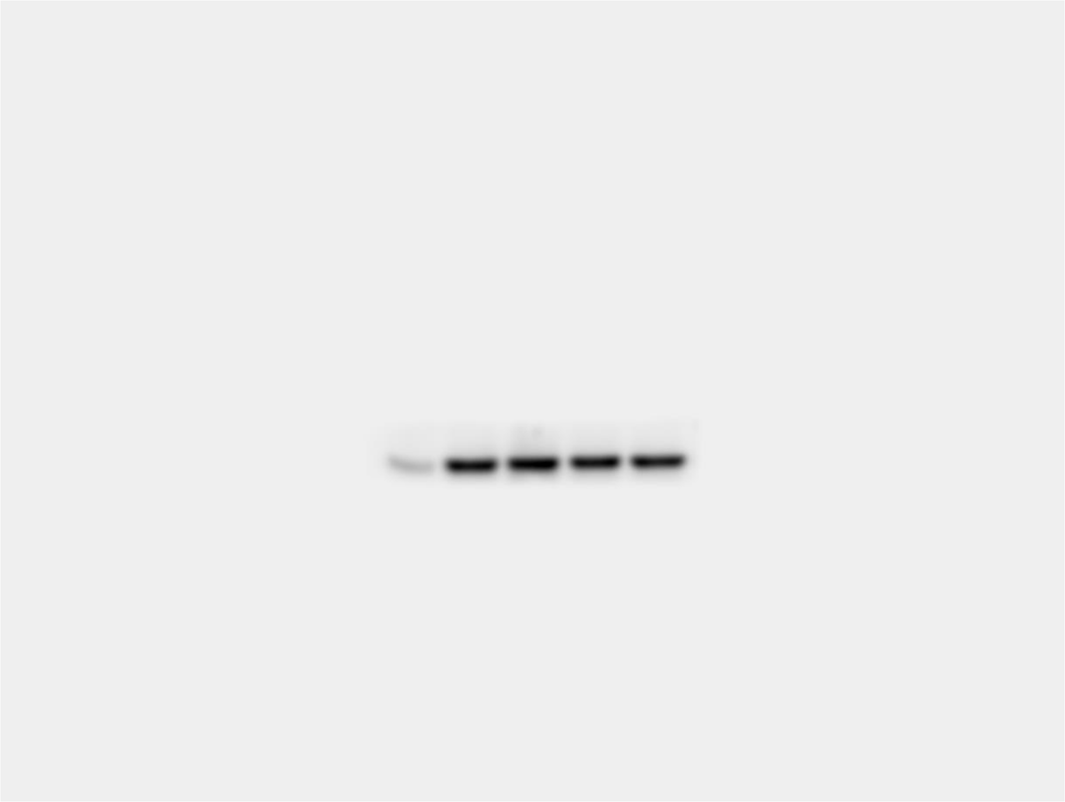

OVCAR-3 N-cad_pub

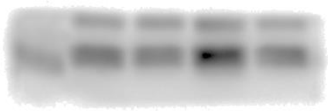

OVCAR-3 slug_pub

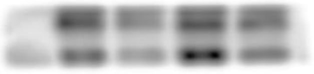

OVCAR-3 snail_pub

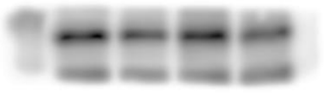

OVCAR-3 twist_pub

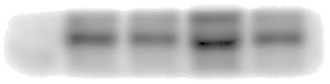

OVCAR-3 vimentin_pub

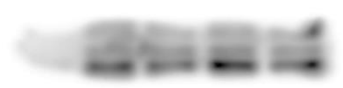

SKOV3 E-cad_pub

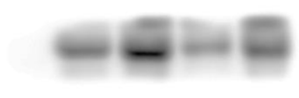

SKOV3 GAPDH_pub

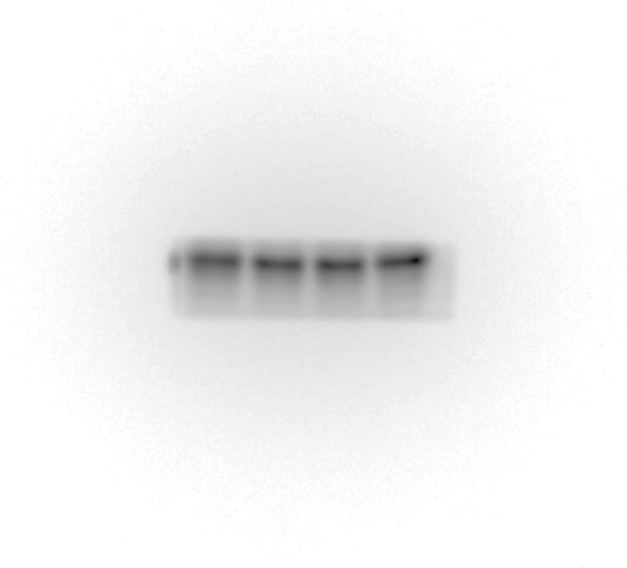

SKOV3 N-cad_pub

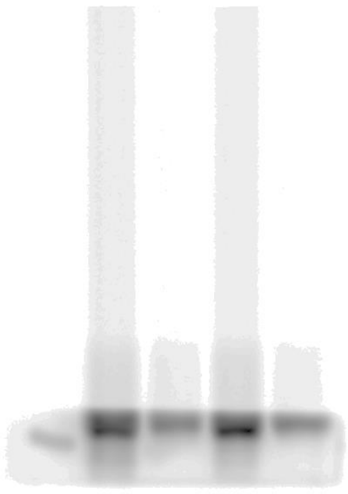

SKOV3 slug_pub

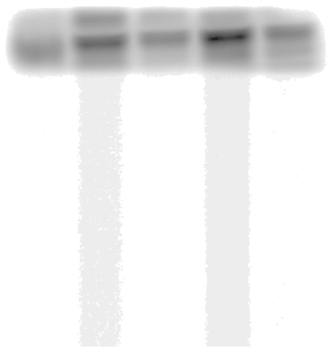

SKOV3 snail_pub

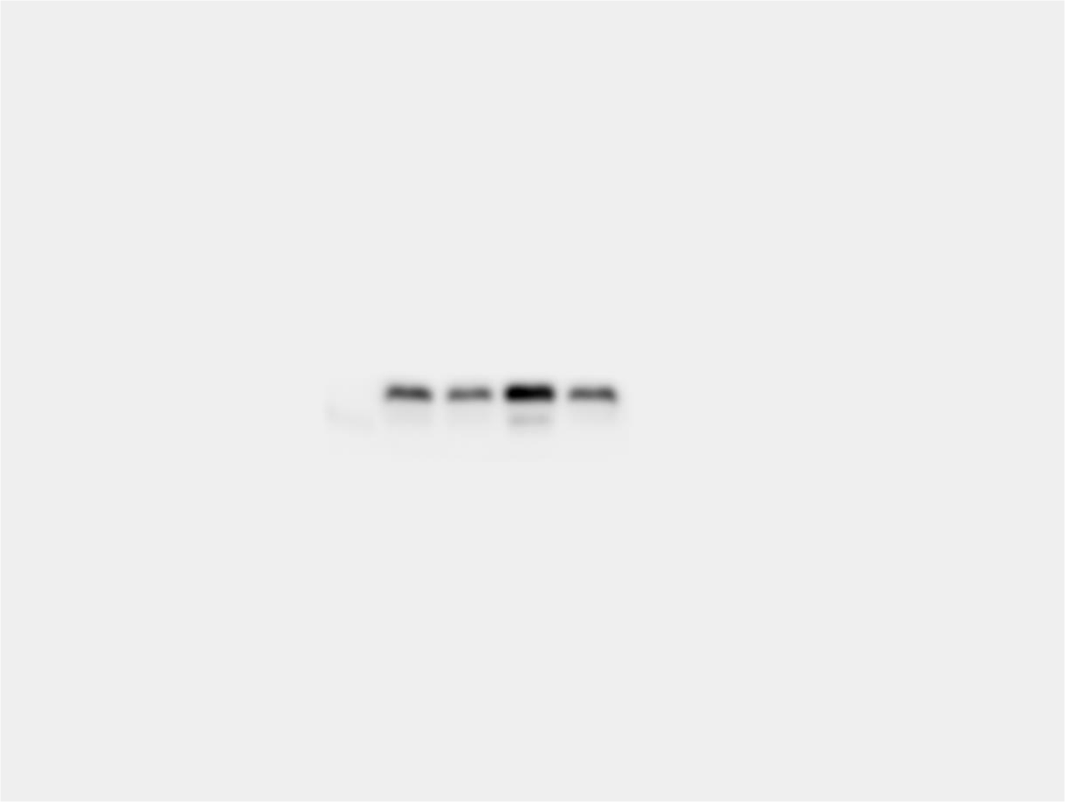

SKOV3 twist_pub

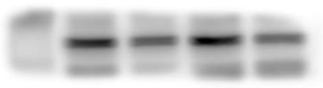

SKOV3 vimentin_pub

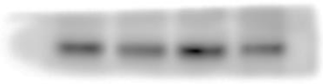

Figure S2A

Fam172A A2780 SKOV3 OVCARE-3_pub

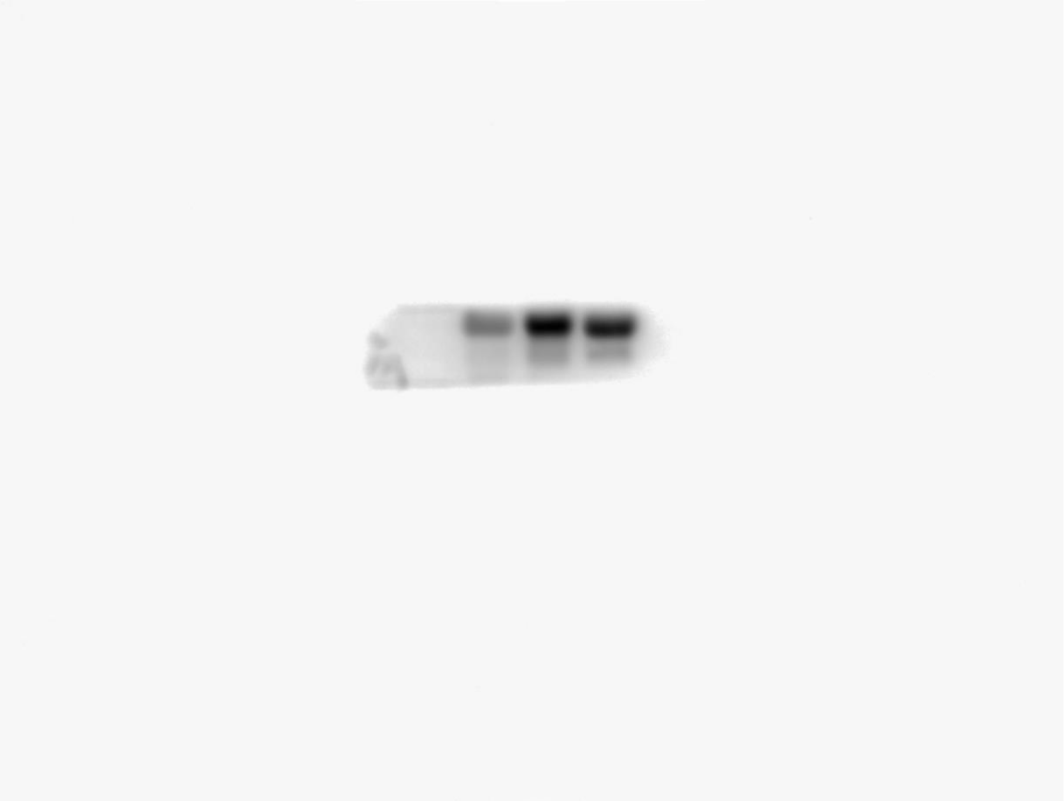

gapdh_pub

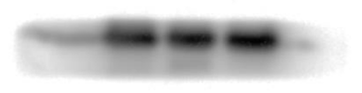

Figure S2B

Fam172A _pub

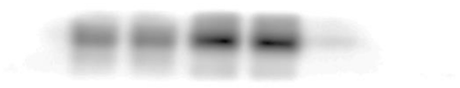

Gapdh

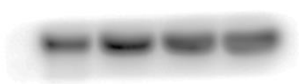

FamOVsi_pub

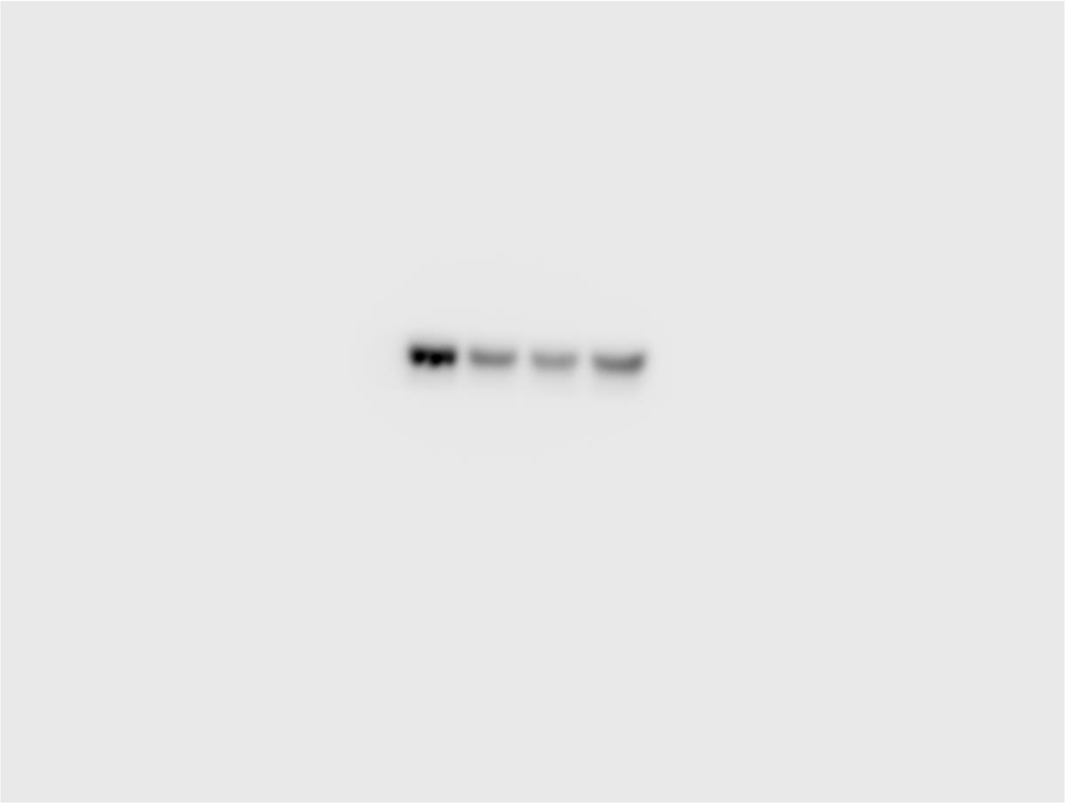

gapdh OVsi_pub

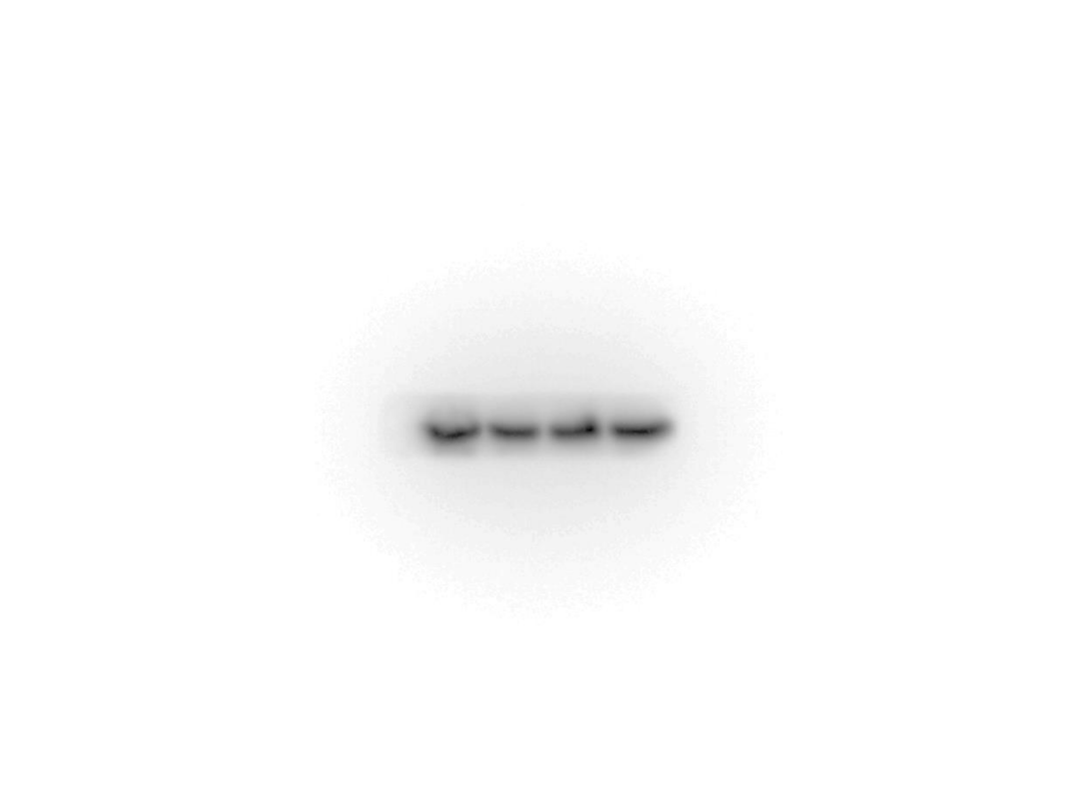

FAM SKO si_pub

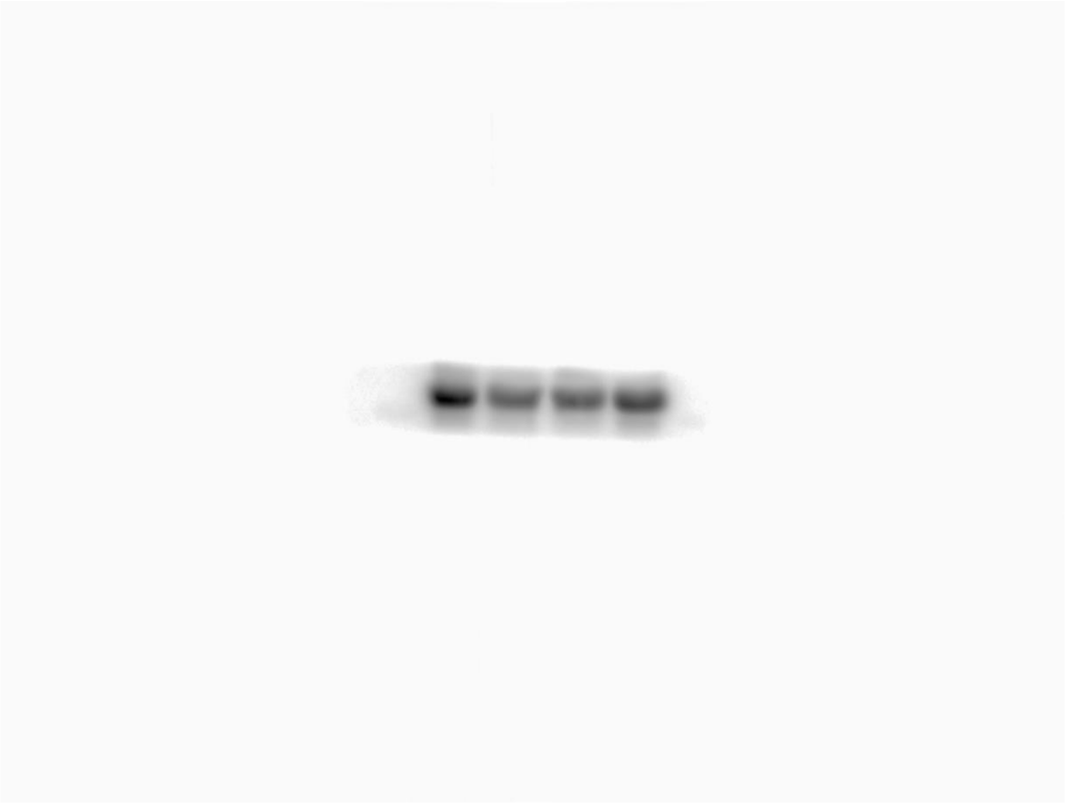

gapdhSKOsi_pub

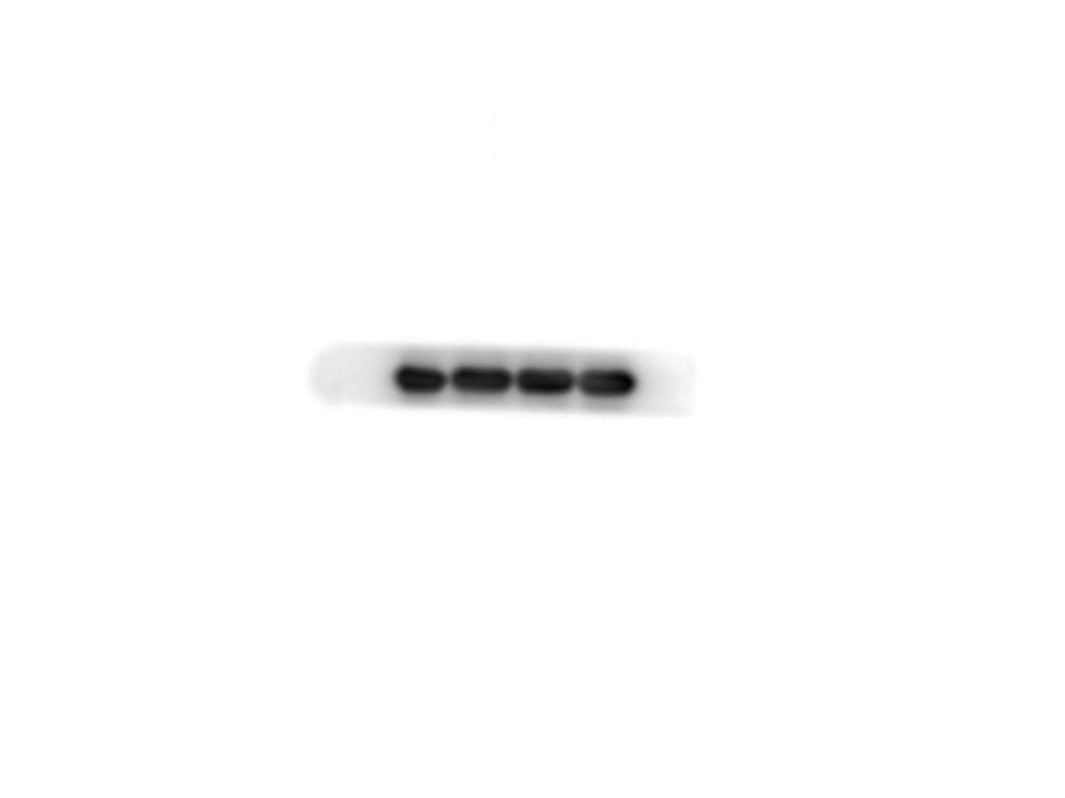

Figure S2D

FAM172A OE_pub

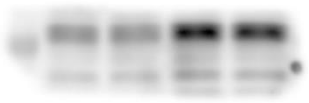

gapdh_pub

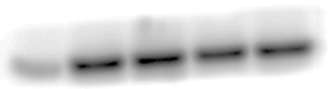

OV sh fam_pub

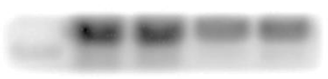

OV sh gapdh_pub

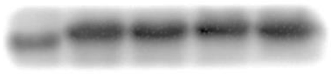

FAM sko sh_pub

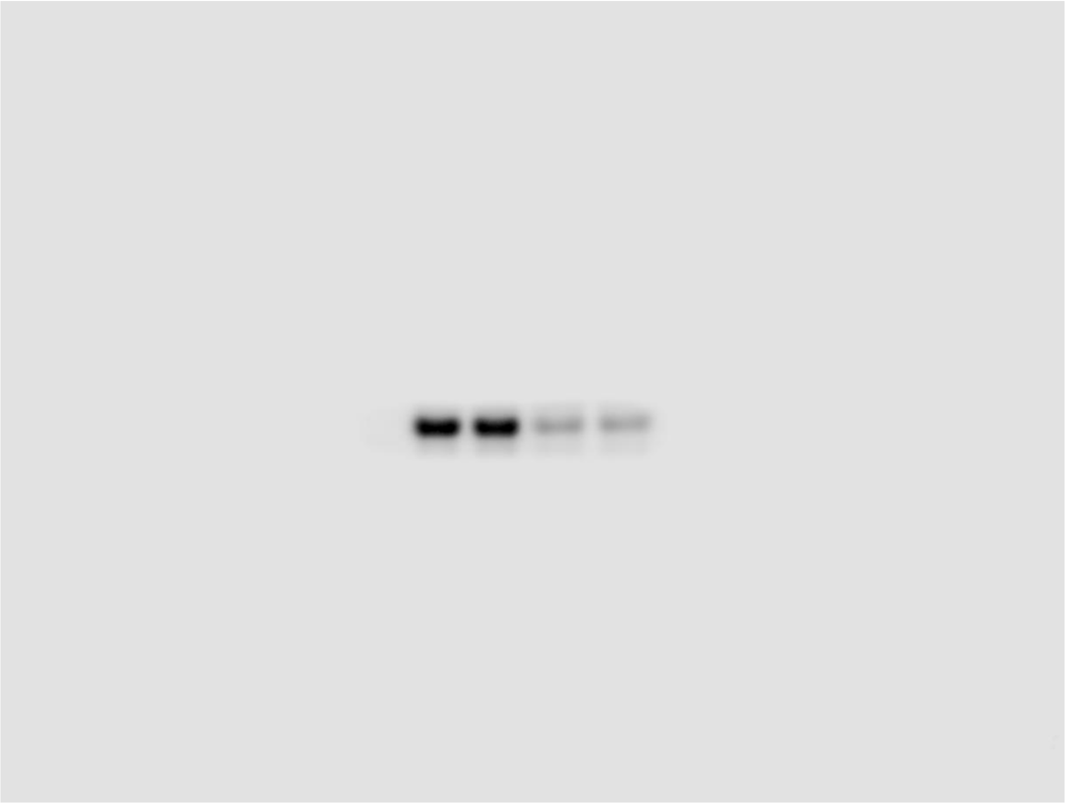

gapdh skosh_pub

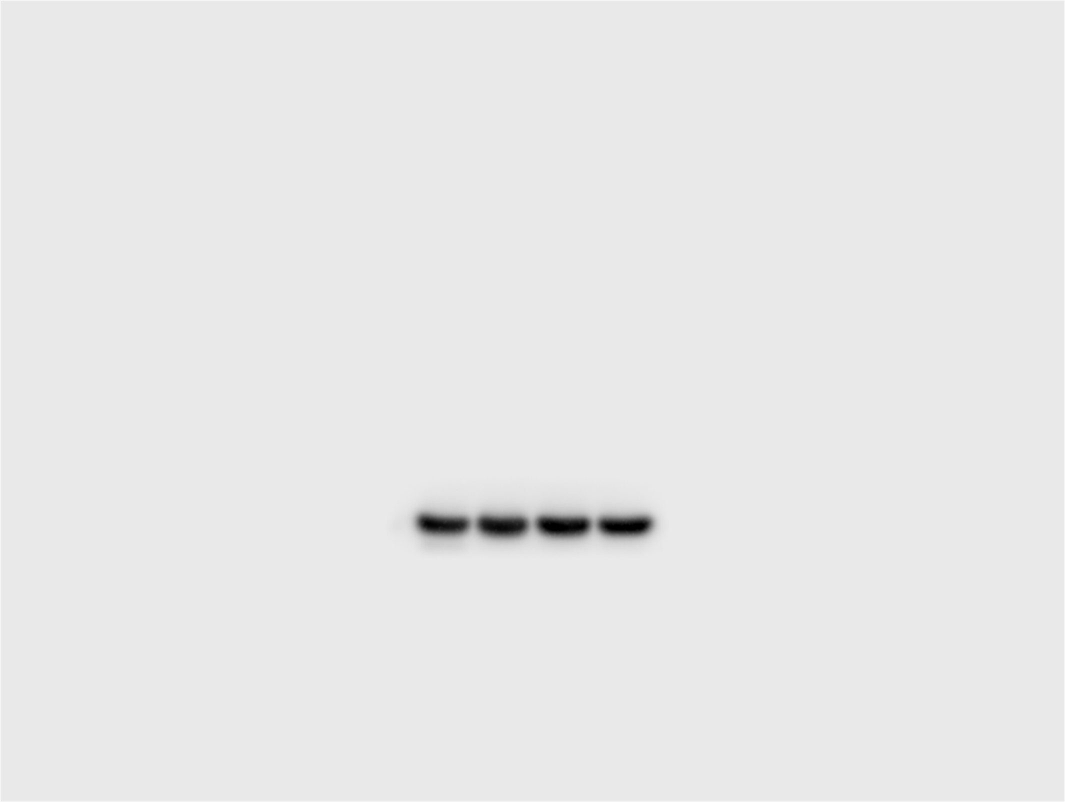

Figure S3

A2780 P53_pub

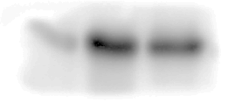

A2780 CDK4_pub

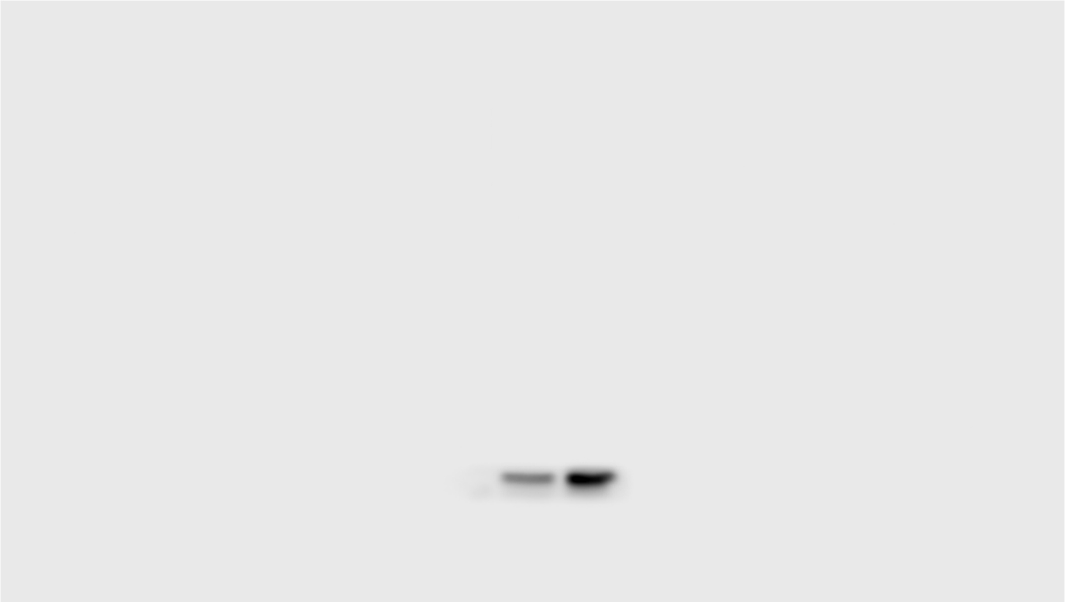

A2780 CDK6_pub

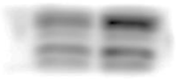

A2780 cyclinD1_pub

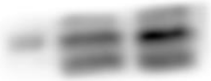

A2780 cyclinE1_pub

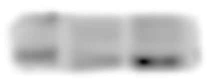

A2780 fam_pub

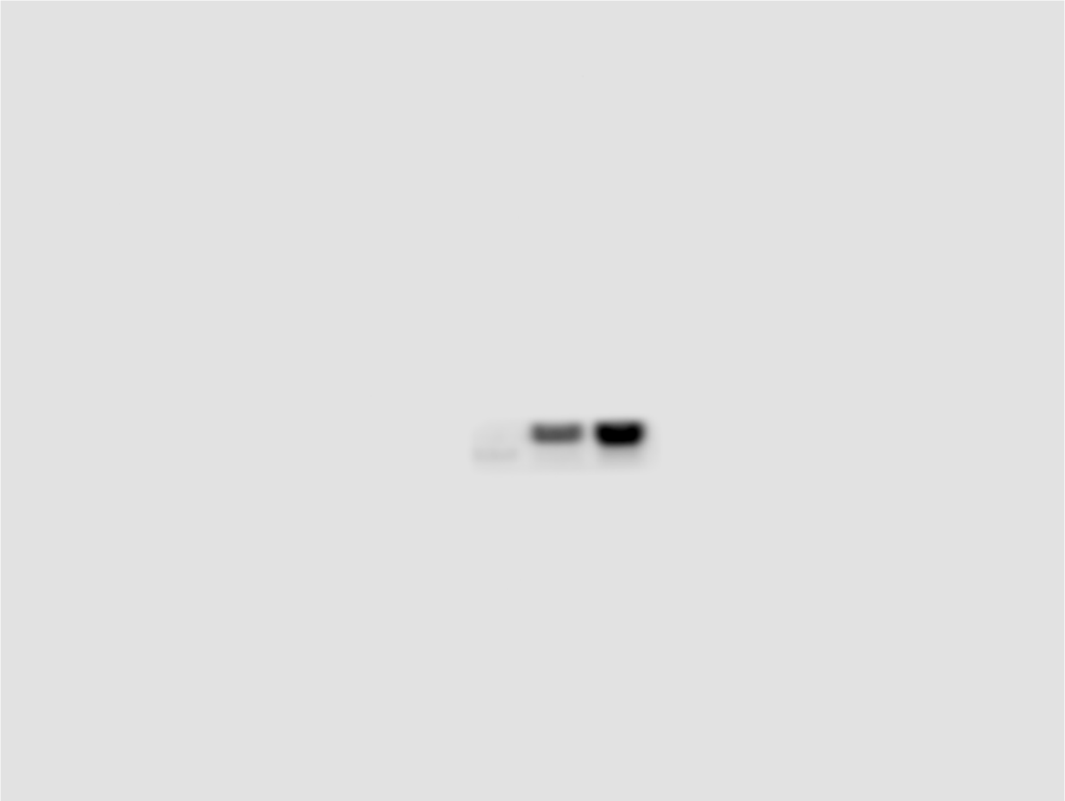

A2780 gapdh_pub

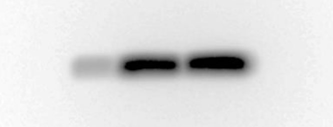

A2780 P21_pub

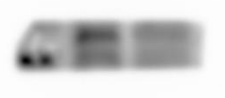

A2780 P27_pub

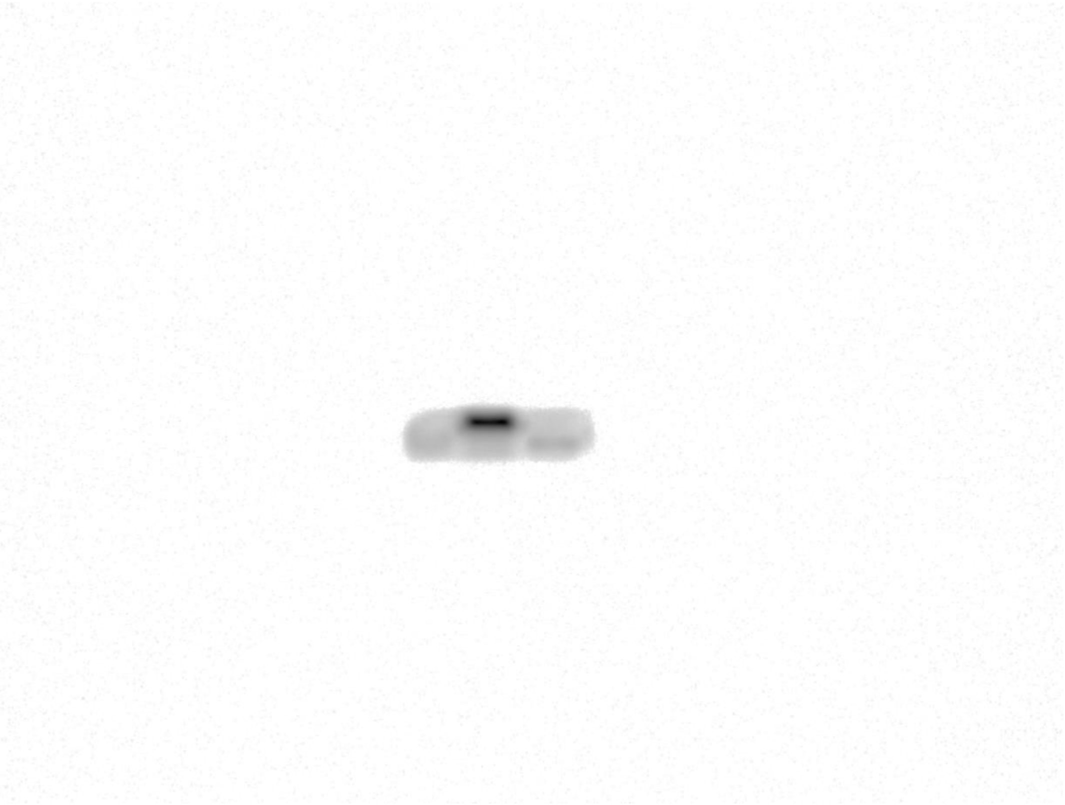

OV CDK4_pub

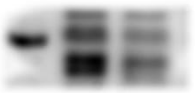

OV CDK6_pub

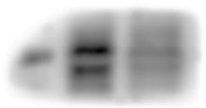

OV cyclinD1_pub

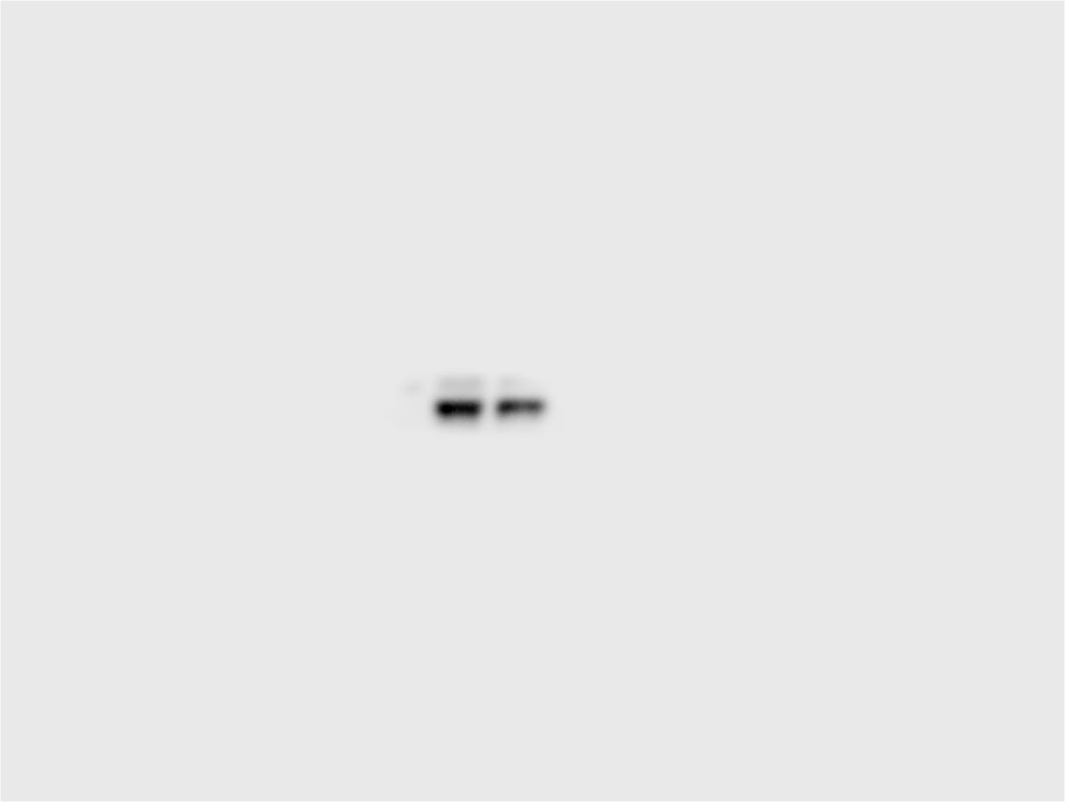

OV cyclinE1_pub

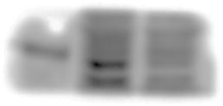

OV fam_pub

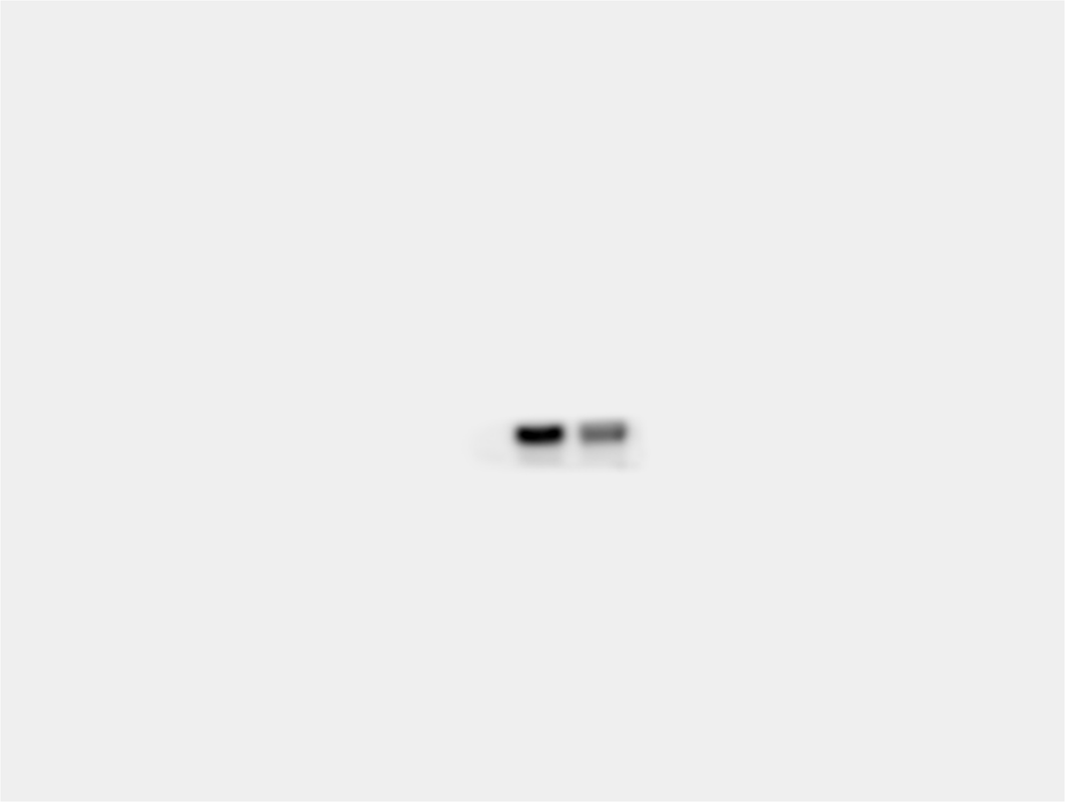

OV gapdh_pub

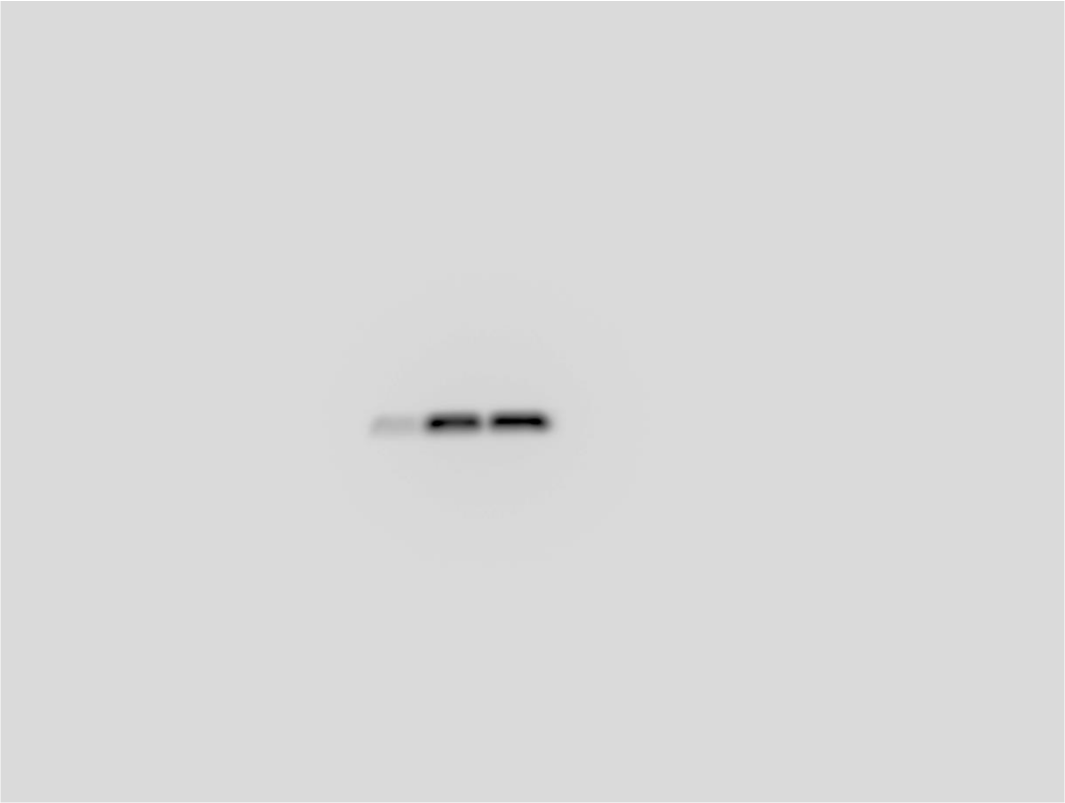

OV P21_pub

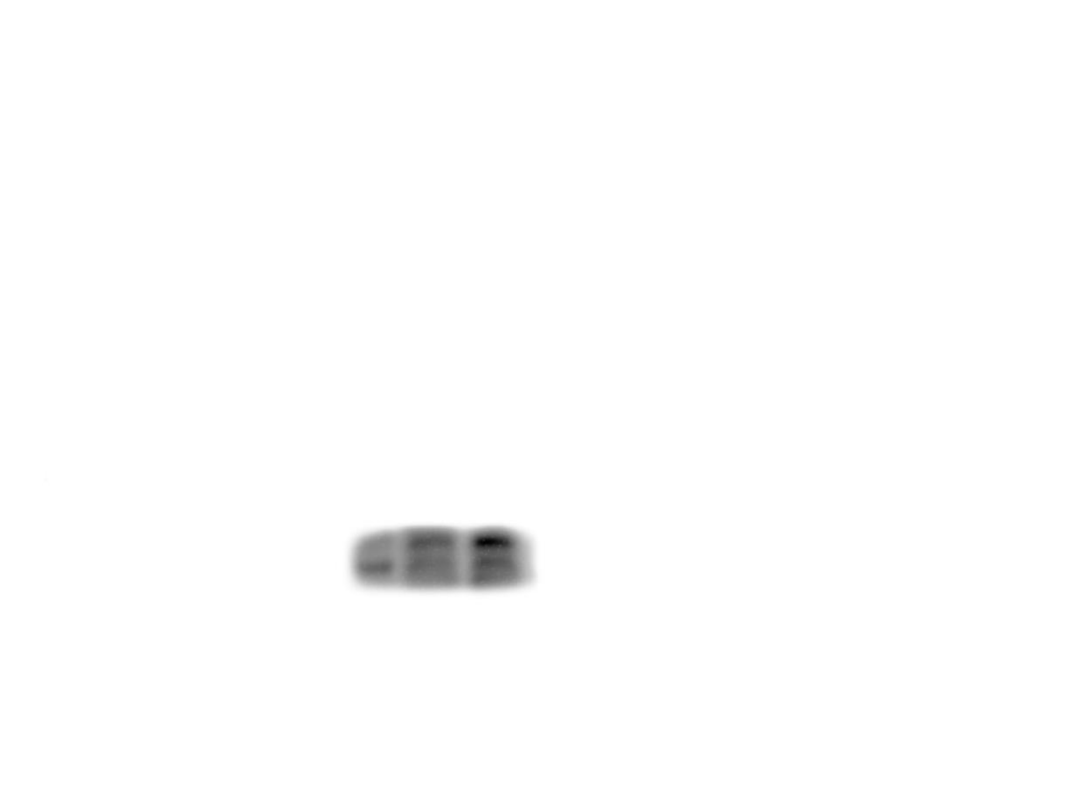

OV P27_pub

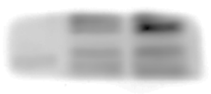

OV P53_pub

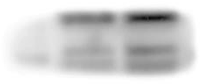

SKOV3 cdk4_pub

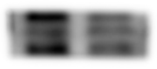

SKOV3 CDK6_pub

SKOV3 cyclinD1_pub

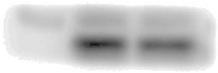

SKOV3 cyclinE1_pub

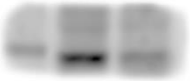

SKOV3 fam_pub

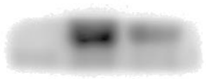

SKOV3 gapdh_pub

SKOV3 P21_pub

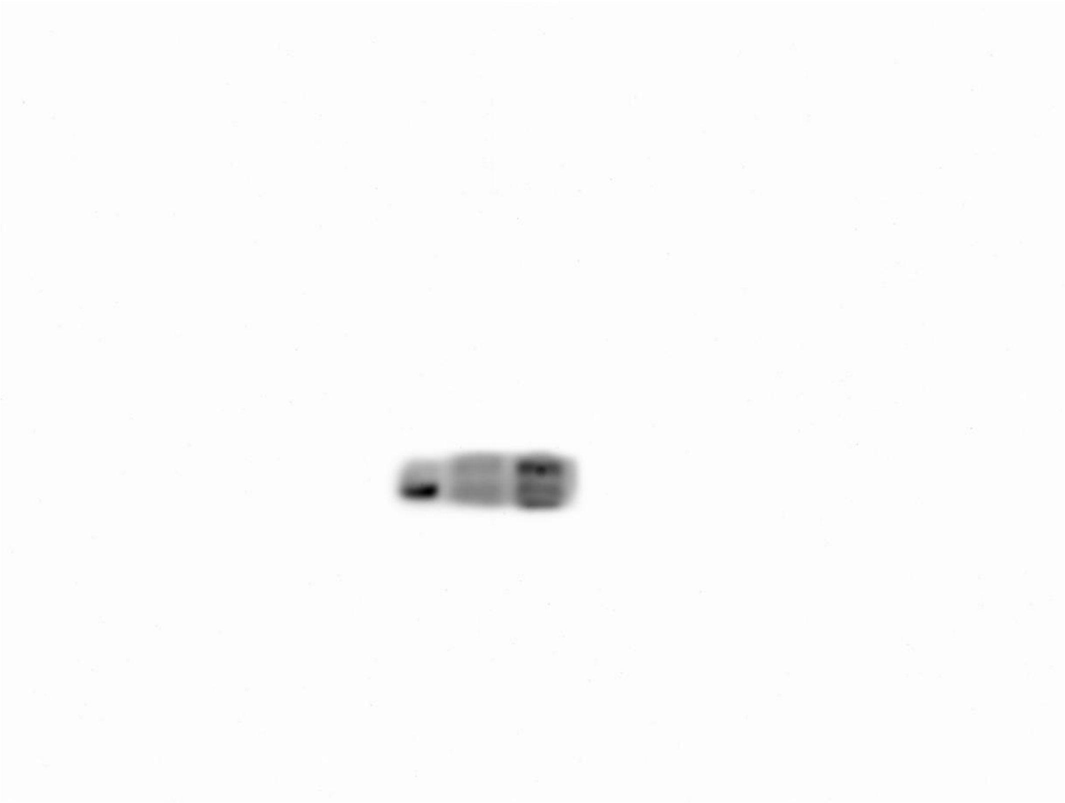

SKOV3 P27_pub

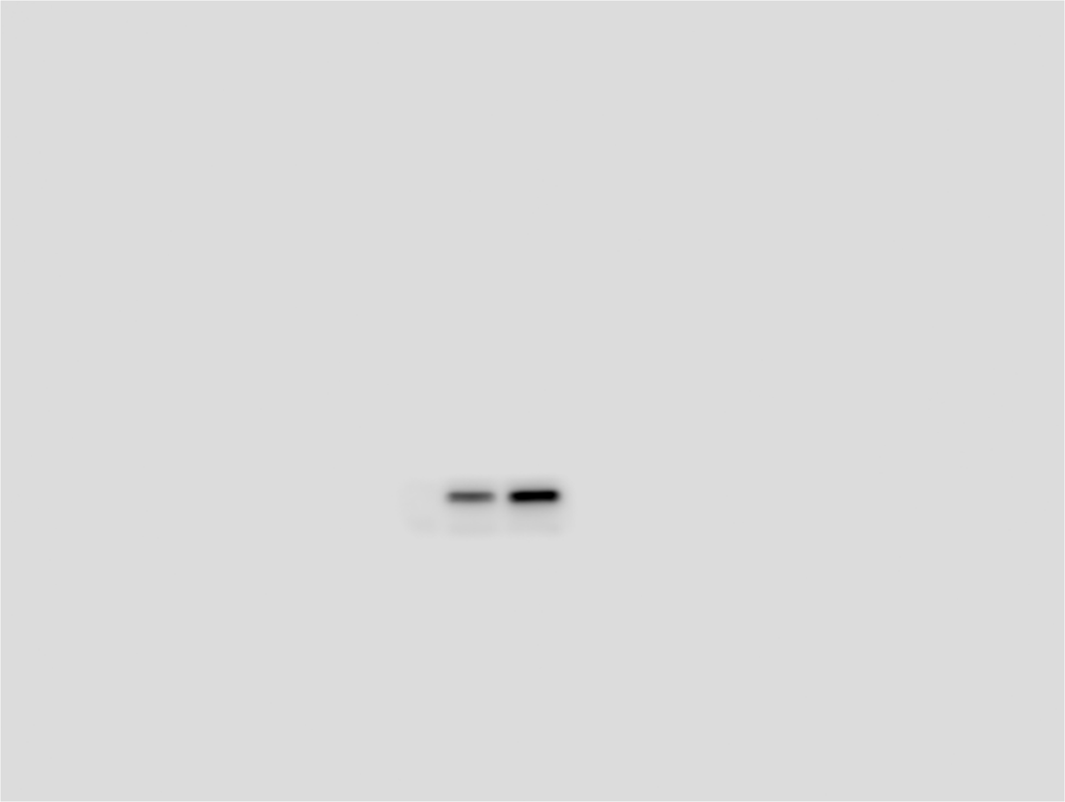

SKOV3 p53_pub

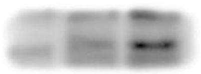

Supplement: Supplementary file 6 — Supplementary Material 6 [file 41598_2025_26676_MOESM6_ESM.pdf]
