# Supplementary material for: FAM172A promotes epithelial ovarian cancer progression and induces platinum resistance via the PI3K/AKT pathway
Source: Sci Rep. 2025 Dec 3;15:43128. doi: 10.1038/s41598-025-26676-9 (PMC12678607; doi:10.1038/s41598-025-26676-9)
Supplement: Supplementary file 7 — Supplementary Material 7 [file 41598_2025_26676_MOESM7_ESM.docx]

**Table S1. Correlation between clinical and pathological features, FAM172A expression and platinum resistance in patients.**

|  |  | **n** | **FAM172A expression** | | ***χ²*** | ***p*** |
| --- | --- | --- | --- | --- | --- | --- |
|  |  |  | **Low** | **High** |  |  |
| Age(years) |  | 150 |  |  | 0.193 | 0.659 |
|  | ≤50 | 42 | 17 (40.5%) | 25 (59.5%) |  |  |
|  | ＞50 | 108 | 48 (44.4%) | 60 (55.6%) |  |  |
| Stage(FIGO) |  | 150 |  |  | 17.408 | **＜0.001** |
|  | Ⅰ/Ⅱ | 28 | 22 (78.6%) | 6 (21.4%) |  |  |
|  | Ⅲ Ⅳ | 122 | 43 (35.2%) | 79 (64.8%) |  |  |
| Histological pattern | | 147 |  |  | 4.427 | **0.035** |
|  | Non-serous | 42 | 24 (57.1%) | 18 (42.9%) |  |  |
|  | Serous | 105 | 40 (38.1%) | 65 (61.9%) |  |  |
| Histological grade | | 145 |  |  | 2.032 | 0.154 |
|  | Ⅰ/Ⅱ | 15 | 9 (60%) | 6 (40%) |  |  |
|  | Ⅲ | 130 | 53 (40.8%) | 77 (59.2%) |  |  |
| Tumor size |  | 134 |  |  | 2.475 | 0.115 |
|  | ＜10cm | 74 | 27 (36.5%) | 47 (63.5%) |  |  |
|  | ≥10cm | 60 | 30 (50%) | 30 (50%) |  |  |
| Ki67 |  | 127 |  |  | 0.013 | 0.908 |
|  | ≤20% | 21 | 9 (42.9%) | 12 (57.1%) |  |  |
|  | ＞20% | 106 | 44 (41.5%) | 62 (58.5%) |  |  |
| Platinum sensitivity | | 150 |  |  | 8.236 | **0.004** |
|  | sensitive | 136 | 64 (47.1%) | 72 (52.9%) |  |  |
|  | resistance | 14 | 1 (7.1%) | 13 (92.9%) |  |  |
| CA125 |  | 150 |  |  | 4.848 | **0.027** |
|  | ≤500U/mL | 70 | 37 (52.9%) | 33 (47.1%) |  |  |
|  | ＞500U/mL | 80 | 28 (35%) | 52 (65%) |  |  |

**Table S2. Univariate and multivariate COX regression analysis of survival factors in patients with ovarian epithelial cancer.**

| **Characteristics** | **Total(N)** | **Univariate analysis** | | **Multivariate analysis** | |
| --- | --- | --- | --- | --- | --- |
|  |  | **Hazard ratio (95% CI)** | **P value** | **Hazard ratio (95% CI)** | **P value** |
| Age(years) | 150 |  |  |  |  |
| ≤50 | 42 | Reference |  |  |  |
| ＞50 | 108 | 1.432 (0.712 - 2.883) | 0.314 |  |  |
| Stage(FIGO) | 150 |  |  |  |  |
| Ⅰ/Ⅱ | 28 | Reference |  | Reference |  |
| Ⅲ/Ⅳ | 122 | 2.165 (0.918 - 5.107) | 0.078 | 1.173 (0.469 - 2.934) | 0.734 |
| Histological pattern | 147 |  |  |  |  |
| Non-serous | 42 | Reference |  |  |  |
| Serous | 105 | 0.657 (0.358 - 1.204) | 0.174 |  |  |
| Histological grade | 145 |  |  |  |  |
| Ⅰ/Ⅱ | 15 | Reference |  |  |  |
| Ⅲ | 130 | 0.681 (0.266 - 1.747) | 0.424 |  |  |
| Tumor size | 134 |  |  |  |  |
| ＜10cm | 74 | Reference |  |  |  |
| ≥10cm | 60 | 0.898 (0.484 - 1.665) | 0.732 |  |  |
| Ki67 | 127 |  |  |  |  |
| ≤20% | 21 | Reference |  |  |  |
| ＞20% | 106 | 0.918 (0.381 - 2.209) | 0.848 |  |  |
| Platinum sensitivity | 150 |  |  |  |  |
| sensitive | 136 | Reference |  | Reference |  |
| resistance | 14 | 7.761 (3.496 - 17.230) | **< 0.001** | 6.873 (2.942 - 16.057) | **< 0.001** |
| CA125 | 150 |  |  |  |  |
| ≤500U/mL | 70 | Reference |  | Reference |  |
| ＞500U/mL | 80 | 2.423 (1.297 - 4.530) | **0.006** | 2.372 (1.223 - 4.598) | **0.011** |
| FAM172A | 150 |  |  |  |  |
| Low | 65 | Reference |  | Reference |  |
| High | 85 | 3.415 (1.749 - 6.669) | **< 0.001** | 2.231 (1.086 - 4.584) | **0.029** |
|  |  |  |  |  |  |
|  |  |  |  |  |  |
|  |  |  |  |  |  |
| Table3 |  |  |  |  |  |

**Table S3. Forest plot of factors affecting survival of patients with ovarian epithelial cancer.**

| **Age(year)** | 150 |  |  |  | |
| --- | --- | --- | --- | --- | --- |
| ≤50 | 42 | 0.698 (0.347 - 1.405) |  | 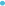 | 0.314 |
| ＞50 | 108 | Reference |  |  | |
| **Stage(FIGO)** | 150 |  |  |  | |
| Ⅰ/Ⅱ | 28 | Reference |  |  | |
| Ⅲ/Ⅳ  **Histological pattern** | 122  147 | 2.165 (0.918 - 5. 107) |  | 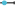 | 0.078 |
| Non-serous | 42 | Reference |  |  |  |
| Serous | 105 | 0.657 (0.358 - 1.204) |  | 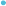 | 0.174 |
| **Histological grade** | 145 |  |  |  |  |
| Ⅰ/Ⅱ | 15 | Reference |  |  |  |
| Ⅲ | 130 | 0.681 (0.266 - 1. 747) |  |  | 0.424 |
| **Tumor size** | 134 |  |  |  |  |
| ＜10cm | 74 | 1.114 (0.601 - 2.066) |  |  | 0.732 |
| ≥10cm | 60 | Reference |  |  |  |
| **Ki67**  ≤20% | 127  21 | 1.090 (0.453 - 2.622) |  | 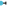 | 0.848 |
| ＞20% | 106 | Reference |  |  |  |
| **Platinum sensitivity** | 150 |  |  |  |  |
| sensitive | 136 | Reference |  |  |  |
| Resistance  **CA125** | 14  150 | 7.761 (3.496 - 17.230) |  | 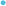 | < 0.001 |
| ≤500U/mL  ＞500U/mL | 70  80 | Reference 2.423 (1.297 - 4.530) |  | 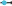 | 0.006 |
| **FAM172A** | 150 |  |  |  |  |
| Low | 65 | Reference |  |  |  |
| High | 85 | 3.415 (1. 749 - 6.669) |  | 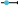 | < 0.001 |
